# Systematic meta-analyses of gene-specific genetic association studies in prostate cancer

**DOI:** 10.18632/oncotarget.7926

**Published:** 2016-03-05

**Authors:** Qiang Hao, Dong Wei, Yaoguang Zhang, Xin Chen, Fan Yang, Ze Yang, Xiaoquan Zhu, Jianye Wang

**Affiliations:** ^1^ Graduate School of Peking Union Medical College, Beijing, 100730, China; ^2^ Department of Urology, Beijing Hospital, Beijing, 100730, China; ^3^ Key Laboratory of Geriatrics, Beijing Hospital and Beijing Institute of Geriatrics, Ministry of Health, Beijing, 100730, China

**Keywords:** prostate cancer, susceptibility, single nucleotide variant, meta-analyses

## Abstract

In the past twenty-five years, over 700 case-control association studies on the risk of prostate cancer have been published worldwide, but their results were largely inconsistent. To facilitate following and explaining these findings, we performed a systematic meta-analysis using allelic contrasts for gene-specific SNVs from at least three independent population-based case-control studies, which were published in the field of prostate cancer between August 1, 1990 and August 1, 2015. Across 66 meta-analyses, a total of 20 genetic variants involving 584,100 subjects in 19 different genes (*KLK3*, *IGFBP3*, *ESR1*, *SOD2*, *CAT*, *CYP1B1*, *VDR*, *RFX6*, *HNF1B*, *SRD5A2*, *FGFR4*, *LEP*, *HOXB13*, *FAS*, *FOXP4*, *SLC22A3*, *LMTK2*, *EHBP1* and *MSMB*) exhibited significant association with prostate cancer. The average summary OR was 1.33 (ranging from: 1.016–3.788) for risk alleles and 0.838 (ranging from: 0.757–0.896) for protective alleles. Of these positive variants, *FOXP4* rs1983891, *LMTK2* rs6465657 and *RFX6* rs339331 had not been previously meta-analyzed. Further analyses with sufficient power design and investigations of the potential biological roles of these genetic variants in prostate cancer should be conducted.

## INTRODUCTION

Prostate cancer (PCa) is the second most common type of solid cancer and the sixth leading cause of cancer death in men worldwide [1]. In the United States in 2015, there were an estimated 220,800 new cases of PCa and 27,540 deaths from this disease (American Cancer Society: Cancer Facts and Figures 2015. Atlanta, GA: American Cancer Society, 2015). Prostate cancer has a clear familial clustering feature [2–4]. The risk of developing PCa rises two to five fold with increasing numbers of affected relatives [5]. Although data from twin studies showed that the genetic contribution to PCa risk is approximately 40% [6, 7], only a few mutations in genes such as *BRCA1*, *BRCA2*, and *HOXB13* definitely account for a small proportion of cases of hereditary prostate cancer [8–10]. Based on a large number of family studies, linkage analyses have identified approximately twenty chromosomal regions harboring potential PCa susceptible loci. However, only a few overlap across studies suggests that PCa exhibits locus heterogeneity [11–13]. Population-based case-control association studies using single nucleotide variants (SNVs) powerfully fine mapped susceptible variants for common diseases [14, 15]. To fine map and identify risk loci of PCa in the population, over 700 candidate gene association studies and 19 GWAS have been published in the past 25 years (between 1990 and 2015). However, the statistical association results claimed by these studies are not consistent. Meta-analysis is a helpful procedure to increase statistical power and to estimate whether the genetic association of a disease is true or not through combining data from the initial reports and subsequent replication studies [16].

To better assess and explain the association of genetic variants with PCa, we have collected and catalogued all population-based case-control genetic association studies, which were published in the field of PCa between August 1, 1990 and August 1, 2015. Subsequently, we systematically meta-analyzed gene-specific SNVs using genotyping data available from at least three independent case-control studies.

## RESULTS

### Publication searches

After screening over 40,000 titles and abstracts, we obtained 728 publications reporting on 217 genetic SNVs in 136 different genes (Supplementary Table S1). Based on our inclusion criteria: 1) at least three independent case-control studies for a gene-specific SNV; 2) each SNV with not more than two alleles distribution, 560 papers containing 19 GWAS publications which reported 66 SNVs in 51 different genes were finally included for the meta-analyses. Next, we searched publications in EMBASE and Cochrane database with the keywords “prostate cancer AND 7 random chosen SNVs or 7 matched genes”, respectively. This process obtained 453 publications from the EMBASE and 3 publications from the Cochrane. 13 of 453 and 2 of 3 publications fulfilled our inclusion criteria. In parallel, PubMed yielded 37 fulfilled publications using the same keywords above. No additional eligible publications from the EMBASE and the Cochrane database were included compared with that from PubMed.

### Meta-analyses

Among the 217 SNVs, 66 variants in 51 different genes with sufficient data were finally included for our meta-analysis (Supplementary Table S1 and Supplementary Figure S1). Combining studies of all ethnic populations, we calculated the summary OR and 95% c.i. values across the 66 included variants using an allelic contrast model. Of these 66 SNVs, 20 in 19 different genes (*KLK3*, *IGFBP3*, *ESR1*, *SOD2*, *CAT*, *CYP1B1*, *VDR*, *RFX6*, *HNF1B*, *SRD5A2*, *FGFR4*, *LEP*, *HOXB13*, *FAS*, *FOXP4*, *SLC22A3*, *LMTK2*, *EHBP1* and *MSMB*) had significant summary ORs (referred to as “positive” association) (Tables 1 and 2; Supplementary Figures S2 and S3). These results suggested an increased or a decreased risk for prostate cancer. The total of sample size involved in these positive meta-analyses was 584,100. The average sample size combining cases with controls across these 20 positive meta-analyses was 29,205 subjects (ranging from 2,581 to 84,391). On average, ∼8 independent studies were included for each SNV (ranging from 3 to 18) (Tables 1 and 2). Fourteen of twenty positive SNVs (summary ORs >1, ranging from 1.039–3.788) increased the risk for prostate cancer by an average of 1.34-fold. Six SNVs (in *VDR*, *FAS*, *KLK3*, *RFX6* and *HNF1B*) with an average protective summary OR of 0.838 (ranging from 0.757–0.896) decreased the risk for PCa by approximately 14%. Apart from 17 SNVs that have been previously meta-analyzed using published data (Tables 1 and 2), 3 additional positive associations were identified by this studies (including *FOXP4* rs1983891, *LMTK2* rs6465657 and *RFX6* rs339331).

**Table 1:** Fixed-effects meta-analyses using allelic contrasts for SNVs showing significant summary ORs (as of August 1, 2015)

Gene	SNV	Putative function	Model	OR (95% c.i.) ^a^ *P-*value	*Q*-value ^b^	Heterogeneity ^c^ *P*-value	Cases versus controls (Number of independent samples)
*SRD5A2*	rs9282858	Exon (p.Ala49Thr)	A vs. G, all ethnicities	1.323 (1.111–1.575) *P* = 0.002	12.337	0.419	4241 vs. 4015 (13)
			A vs. G, all excl. initial study	1.315 (1.104–1.566) *P* = 0.002	11.021	0.442	4135 vs. 3903 (12)
			A vs. G, all excl. HWE study	1.191 (0.969–1.464) *P* = 0.097	7.594	0.474	3785 vs. 3298 (9)
*FGFR4*	rs351855	Exon (p.Gly388Arg)	T vs. C, all ethnicities	1.156 (1.057–1.263) *P* = 0.002	5.358	0.374	2618 vs.2157 (6)
			T vs. C, all excl. initial study	1.129 (1.029–1.238) *P* = 0.010	1.329	0.722	2289 vs. 1966 (4)
*VDR*	rs731236	Promoter	C vs. T, all ethnicities	0.757 (0.645–0.889) *P* = 0.001	7.005	0.637	1198 vs. 1753 (10)
			C vs. T, all excl. initial study	0.743 (0.628–0.879) *P* = 0.001	6.468	0.595	1098 vs. 1551 (9)
			C vs. T, all excl. HWE study	0.760 (0.636–0.908) *P* = 0.003	5.941	0.547	988 vs. 1378 (8)
*LEP*	rs2167270	5′UTR	A vs. G, all ethnicities	1.163 (1.042–1.299) *P* = 0.007	3.723	0.155	1343 vs. 1238 (3)
			A vs. G, all excl. initial study	1.124 (1.001–1.262) *P* = 0.049	0.189	0.663	1200 vs. 1120 (2)
*HOXB13*	rs138213197	Exon (p.Gly84Glu)	A vs. G, all ethnicities	3.788 (2.450–5.855) *P* = 0.000	8.599	0.197	11524 vs. 63753 (7)
			A vs. G, all excl. initial study	3.649 (2.316–5.749) *P* = 0.000	8.284	0.141	9989 vs. 61994 (6)
			A vs. G, all excl. HWE study	3.743 (2.412–5.810) *P* = 0.000	8.450	0.133	10807 vs. 62061 (6)
*FAS*	rs1800682	Promoter	G vs. A, all ethnicities	0.866 (0.773–0.971) *P* = 0.014	3.625	0.163	1451 vs. 1174 (3)
			G vs. A, all excl. initial study	0.928 (0.692–1.245) *P* = 0.619	3.496	0.062	794 vs. 927 (2)
*FOXP4*	rs1983891	Intron	T vs. C, all ethnicities	1.107 (1.065–1.150) *P* = 0.000	1.380	0.502	11128 vs. 13738 (3)
			T vs. C, all excl. initial study	1.104 (1.052–1.157) *P* = 0.000	1.343	0.246	8127 vs. 8323 (2)
*SLC22A3*	rs9364554	Intron	T vs. C, all ethnicities	1.039 (1.005–1.076) *P* = 0.026	3.840	0.428	15763 vs. 50197 (5)
			T vs. C, all excl. initial study	1.033 (0.994–1.074) *P* = 0.097	3.412	0.332	14038 vs. 14798 (4)
*LMTK2*	rs6465657	Intron	T vs. C, all ethnicities	1.060 (1.027–1.094) *P* = 0.000	7.623	0.106	16376 vs. 50128 (5)
			T vs. C, all excl. initial study	1.016 (0.922–1.118) *P* = 0.755	7.457	0.059	14652 vs. 14770 (4)
*EHBP1*	rs721048	Intron	A vs. G, all ethnicities	1.100 (1.057–1.146) *P* = 0.000	6.405	0.269	16385 vs. 50290 (6)
			A vs. G, all excl. initial study	1.098 (1.054–1.144) *P* = 0.000	5.665	0.226	15517 vs. 49412 (5)
*MSMB*	rs10993994	Promoter	T vs. C, all ethnicities	1.210 (1.170–1.250) *P* = 0.000	5.648	0.342	14714 vs. 14463 (6)
			T vs. C, all excl. initial study	1.220 (1.177–1.265) *P* = 0.000	4.130	0.389	11851 vs. 12762 (5)

Note that ‘All ethnicities’ represented that all studies across all ethnic groups were meta-analyzed; ‘all excl. initial study’ represented that the first publication reporting on statistic association for any variant was excluded; ‘all excl. HWE study’ represented that the studies deviating from HWE were excluded.

a the summary OR and 95% c.i. values.

b Q statistic across crude ORs was calculated for each independent study.

c *P* < 0.1 was usually considered as a significant evidence for between-study heterogeneity.

**Table 2:** Random-effects meta-analyses using allelic contrasts for SNVs showing significant summary ORs (as of August 1, 2015)

Gene	SNV	Putative function	Model	OR (95% c.i.) ^a^, *P*-value	*Q*-value ^b^	Heterogeneity ^c^ *P*-value	Cases versus controls (Number of independent samples)
*KLK3*	rs2735839	Intron	A vs. G, all ethnicities	0.795 (0.694–0.911) *P* = 0.001	71.124	0.000	17964 vs. 19099 (9)
			A vs. G, excl. initial study	0.845 (0.731–0.975) *P* = 0.021	27.005	0.000	12842 vs. 13839 (6)
*IGFBP3*	rs2854744	Promoter	C vs. A, all ethnicities	1.169 (1.047–1.304) *P* = 0.005	13.721	0.089	2788 vs. 3020 (9)
			C vs. A, excl. initial study	1.172 (1.035–1.327) *P* = 0.012	13.717	0.056	2481 vs. 2748 (8)
*ESR1*	rs9340799	Intron	G vs. A, all ethnicities	1.151 (1.028–1.288) *P* = 0.014	39.394	0.001	3666 vs. 5066 (16)
			G vs. A, excl. initial study	1.150 (1.022–1.294) *P* = 0.020	39.125	0.000	3584 vs. 4829 (15)
			G vs. A, excl. HWE study	1.150 (1.015–1.303) *P* = 0.028	33.101	0.001	3208 vs. 4257 (13)
*SOD2*	rs4880	Exon (p.Val16Ala)	C vs. T, all ethnicities	1.121 (1.024–1.227) *P* = 0.013	27.234	0.018	4210 vs. 6907 (15)
			C vs. T, excl. initial study	1.111 (1.012–1.221) *P* = 0.027	25.796	0.018	4011 vs. 6716 (14)
			C vs. T, excl. HWE study	1.108 (1.013–1.213) *P* = 0.026	25.138	0.022	4159 vs. 6752 (14)
*CAT*	rs1001179	Promoter	T vs. C, all ethnicities	1.211(1.045–1.404) *P* = 0.011	10.505	0.033	3867 vs.28224 (5)
			T vs. C, excl. initial study	1.270 (1.052–1.534) *P* = 0.013	8.285	0.040	3359 vs. 26821 (4)
			T vs. C, excl. HWE study	1.234 (0.971–1.570) *P* = 0.086	10.454	0.015	2338 vs.3040 (4)
*CYP1B1*	rs1056836	Exon (p.Leu432Val)	G vs. C, all ethnicities	1.129 (1.004–1.270) *P* = 0.042	36.379	0.000	5999 vs. 5438 (11)
			G vs. C, excl. initial study	1.091 (0.986–1.207) *P* = 0.091	25.300	0.003	5949 vs. 5388 (10)
			G vs. C, excl. HWE study	1.132 (0.958–1.337) *P* = 0.144	20.424	0.001	3341 vs. 3220 (6)
*VDR*	rs1544410	Promoter	A vs. G, all ethnicities	0.896 (0.823–0.975) *P* = 0.011	41.674	0.001	7270 vs. 8009 (18)
			A vs. G, excl. initial study	0.888 (0.815–0.969) *P* = 0.007	40.454	0.001	7119 vs. 7835 (17)
			A vs. G, excl. HWE study	0.909 (0.827–0.999) *P* = 0.048	35.691	0.001	6273 vs. 6840 (14)
*RFX6*	rs339331	Intron	T vs. C, all ethnicities	0.854 (0.787–0.927) *P* = 0.000	10.733	0.013	12638 vs. 15897 (4)
			T vs. C, excl. initial study	0.846 (0.743–0.963) *P* = 0.011	8.758	0.013	9637 vs. 10482 (3)
*HNF1B*	rs4430796	Intron	G vs. A, all ethnicities	0.859 (0.793–0.930) *P* = 0.000	55.853	0.000	26822 vs. 57569 (11)
			G vs. A, excl. initial study	0.866 (0.784–0.956) *P* = 0.004	48.460	0.000	17488 vs. 50505 (10)

Note that ‘All ethnicities’ represented that all studies across all ethnic groups were meta-analyzed; ‘all excl. initial study’ represented that the first publication reporting on statistic association for any variant was excluded; ‘all excl. HWE study’ represented that the studies deviating from HWE were excluded.

a the summary OR and 95% c.i. values.

b Q statistic across crude ORs was calculated for each independent study.

c *P* < 0.1 was usually considered as a significant evidence for between-study heterogeneity.

After the initial publications were removed, twelve (60%) of these twenty positive meta-analyses showed notable changes in statistical significance. The *P* values of eleven variants clearly decreased, and three positive variants (rs1800682 in *FAS*, rs9364554 in *SLC22A3* and rs6465657 in *LMTK2*) became insignificant (*P* >0.05) (Tables 1 and 2), due to a substantial decrease in sample size (range: 34-56%). By contrast, one variant rs1544410 in *VDR* became more significant (*P* value changed from 0.011 to 0.007). Of twenty positive SNVs, eleven variants showed study homogeneity (*P* >0.1 in Q statistic) in the meta-analyses combining all ethnic groups, and their summary ORs and 95% c.i. values were calculated using the fixed-effect model (Table 1). Nine other positive associations showed large between-study heterogeneity (*P* < 0.1 in Q statistic), and the summary ORs of them were calculated using the random-effect model (Table 2).

Forty-six SNVs in thirty-five different genes did not show significant summary ORs (referred to as negative SNVs) utilizing random-effect models, after all published population-based case-control studies were allelic-specifically meta-analyzed in all ethnic groups (Supplementary Table S2). The combined sample sizes of these negative meta-analyses ranged from 1,070-32,738 (mean of 9,096 individuals), and the average case-control studies were 15 (range from 3 to 33). Thirty-eight of these 46 variants were close to statistical significance. The average of OR was 1.05 (ranging from 0.921-1.249).

Eight of the twenty positive variants in the control subjects showed deviation from Hardy-Weinberg equilibrium (HWE). While four variants (rs9282858 in *SRD5A2*, rs1001179 in *CAT*, rs1056836 in *CYP1B1* and rs1544410 in *VDR*) became insignificant after the exclusion of HWE-deviation studies (Tables 1 and 2). For positive variants that still showed between-study heterogeneity after excluding the HWE-deviation studies, we then stepwise removed outlier studies until homogeneity was obtained. Eight (∼90%) of nine variants with heterogeneity were corrected (Heterogeneity *P*-value >0.1), except *RFX6* rs339331. Although the overwhelming majority of significant results only showed slight changes after the process, one positive variant (rs9340799 in *ESR1*) lost significant effect size (Supplementary Table S4).

**Figure 1:**
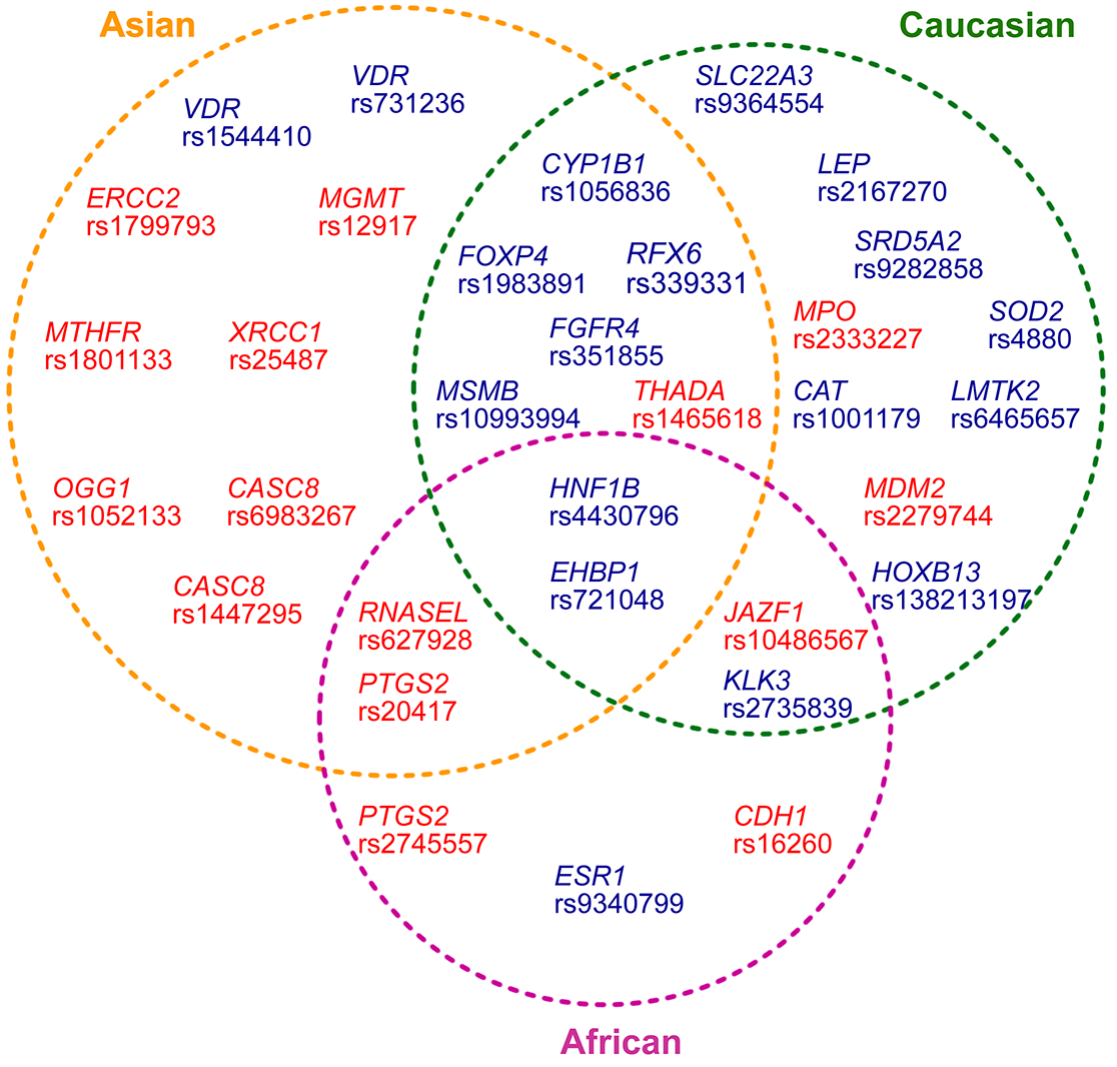
Distribution of thirty-three prostate cancer-associated variants in different ethnic ancestries The variants showing statistical significance in both the initial meta-analyses combining all ethnic ancestries and in the analyses based on classified racial groups were indicated in dark blue text. The positive variants that only showed statistic significance in the analyses based on classified racial groups were indicated in red.

Apart from two positive variants (*HOXB13* rs138213197 and *LEP* rs2167270) for which all published case-control studies were of Caucasian-ancestry, we stratified other eighteen positive variants into racial subgroups and re-meta-analyzed summary ORs and 95% c.i. values. Two variants (in *EHBP1* and *HNF1B*) consistently showed significant association (*P* values: 0.036 to 0.000) with PCa across Asian-ancestry, Caucasian-ancestry and African-ancestry; Five variants (*RFX6* rs339331, *FOXP4* rs1983891, *CYP1B1* rs1056836, *MSMB* rs10993994 and *FGFR4* rs351855) showed positive results in both Asian-ancestry and Caucasian-ancestry; Six variants in *KLK3*, *SRD5A2*, *SOD2*, *CAT*, *SLC22A3* and *LMTK2* only maintained statistical significance in Caucasian-ancestry; Two variants in the *VDR* gene showed positive results only in Asian ancestry; While the variant rs9340799 in *ESR1* was significantly associated with PCa only in African-ancestry patients. Of note, no positive results were seen in *IGFBP3* rs2854744 and in *FAS* rs1800682 in all ethnic subgroups (Table 3, Figure 1 and Supplementary Figure S5).

**Table 3:** Meta-analyses based on ethnic subgroups using allelic contrasts for20 positive SNVs (as of August 1, 2015)

Gene	SNV	Model	OR (95% c.i.) *P*-value ^b^	*Q*-value ^c^	Heterogeneity ^d^ *P*-value	Cases versus controls (Number of independent samples)
*KLK3 ^a^*	rs2735839	African	0.783 (0.625–0.981) *P* = 0.033	0.000	1.000	454 vs. 301 (1)
		Asian	0.895 (0.649–1.234) *P* = 0.498	13.238	0.001	1501 vs. 2047 (3)
		Caucasian	0.736 (0.571–0.947) *P* = 0.017	38.416	0.000	13676 vs. 14364 (3)
		Mixed	0.767 (0.678–0.867) *P* = 0.000	1.094	0.296	2333 vs.2387 (2)
*SRD5A2*	rs9282858	African	0.778 (0.042–14.236) *P* = 0.865	0.000	1.000	30 vs. 261 (1)
		Caucasian	1.325 (1.113–1.578) *P* = 0.002	12.209	0.348	4211 vs. 3754 (12)
*ESR1 ^a^*	rs9340799	African	1.531 (1.132–2.071) *P* = 0.006	0.064	0.800	129 vs. 422 (2)
		Asian	1.157 (0.928–1.442) *P* = 0.194	4.574	0.206	537 vs. 850 (4)
		Caucasian	1.113 (0.974–1.271) *P* = 0.116	27.623	0.001	3000 vs. 3794 (10)
*SOD2 ^a^*	rs4880	African	1.100 (0.803–1.509) *P* = 0.552	3.231	0.199	182 vs. 672 (3)
		Caucasian	1.153 (1.030–1.291) *P* = 0.013	20.386	0.016	3372 vs. 4781 (10)
		Mixed	1.055 (0.825–1.351) *P* = 0.668	2.398	0.122	656 vs.1454 (2)
*CAT ^a^*	rs1001179	Asian	1.064 (0.780–1.452) *P* = 0.694	0.000	1.000	1417 vs. 1008 (1)
		Caucasian	1.338 (1.053–1.700) *P* = 0.017	7.504	0.023	1942 vs. 25813 (3)
		Mixed	1.054 (0.884–1.256) *P* = 0.559	0.000	1.000	508 vs.1403 (1)
*FGFR4*	rs351855	African	1.154 (0.731–1.823) *P* = 0.538	0.004	0.948	191 vs. 174 (2)
		Asian	1.244 (1.022–1.514) *P* = 0.030	0.000	1.000	492 vs. 344 (1)
		Caucasian	1.133 (1.022–1.255) *P* = 0.018	4.666	0.097	1935 vs. 1639 (3)
*VDR*	rs731236	Asian	0.767 (0.640–0.920) *P* = 0.004	6.912	0.546	1065 vs.1596 (9)
		Caucasian	0.722 (0.513–1.017) *P* = 0.062	0.000	1.000	133 vs. 157 (1)
*CYP1B1 ^a^*	rs1056836	Asian	1.621 (1.206–2.179) *P* = 0.001	0.991	0.320	236 vs.355 (2)
		Caucasian	1.080 (1.022–1.141) *P* = 0.006	28.384	0.000	5763 vs. 5083 (9)
*VDR ^a^*	rs1544410	African	0.886 (0.590–1.330) *P* = 0.559	5.187	0.075	375 vs. 361 (3)
		Asian	0.533 (0.326–0.871) *P* = 0.012	9.268	0.026	665 vs. 760 (4)
		Caucasian	0.959 (0.910–1.009) *P* = 0.108	7.876	0.547	5791 vs. 6411 (10)
		Mixed	0.873 (0.723–1.053) *P* = 0.155	0.000	1.000	439 vs.477 (1)
*IGFBP3 ^a^*	rs2854744	African	1.166 (0.965–1.410) *P* = 0.111	0.036	0.849	451 vs. 414 (2)
		Asian	1.123 (0.969–1.302) *P* = 0.124	2.276	0.320	1196 vs. 1199 (3)
		Caucasian	1.294 (0.755–2.217) *P* = 0.349	2.987	0.084	218 vs. 380 (2)
		Mixed	1.107 (0.861–1.424) *P* = 0.427	3.983	0.046	923 vs.1027 (2)
*LEP*	rs2167270	Caucasian only ^e^	1.163 (1.042–1.299) *P* = 0.007	3.723	0.155	1343 vs. 1238 (3)
*HOXB13*	rs138213197	Caucasian only ^e^	3.788 (2.450–5.855) *P* = 0.000	8.599	0.197	11524 vs. 63753 (7)
*FAS*	rs1800682	Asian	0.878 (0.766–1.007) *P* = 0.063	3.496	0.062	794 vs. 927 (2)
		Caucasian	0.839 (0.682–1.003) *P* = 0.099	0.000	1.000	657 vs. 247 (1)
*RFX6 ^a^*	rs339331	Asian	0.831 (0.787–0.877) *P* = 0.000	1.666	0.435	4814 vs. 7867 (3)
		Caucasian	0.929 (0.885–0.976) *P* = 0.003	0.000	1.000	7824 vs. 8030 (1)
*FOXP4*	rs1983891	Asian	1.122 (1.054–1.193) *P* = 0.000	1.084	0.298	3290 vs. 5702 (2)
		Caucasian	1.097 (1.045–1.152) *P* = 0.000	0.000	1.000	7838 vs. 8036 (1)
*SLC22A3*	rs9364554	African	1.000 (0.861–1.161) *P* = 0.999	0.000	1.000	2652 vs. 2659 (1)
		Asian	0.945 (0.847–1.054) *P* = 0.312	0.109	0.741	1392 vs. 1675 (2)
		Caucasian	1.053 (1.015–1.093) *P* = 0.006	0.066	0.797	11719 vs. 45863 (2)
*LMTK2*	rs6465657	African	1.000 (0.898–1.114) *P* = 0.998	0.000	1.000	3266 vs. 2631 (1)
		Asian	0.911 (0.785–1.057) *P* = 0.221	1.177	0.278	1392 vs. 1674 (2)
		Caucasian	1.075 (1.039–1.112) *P* = 0.000	0.736	0.391	11718 vs. 45823 (2)
*EHBP1*	rs721048	African	1.264 (1.062–1.503) *P* = 0.008	0.000	0.998	2951 vs. 2994 (2)
		Asian	1.338 (1.020–1.757) *P* = 0.036	0.113	0.736	1392 vs. 1677 (2)
		Caucasian	1.087 (1.042–1.133) *P* = 0.000	1.522	0.217	12042 vs. 45619 (2)
*HNF1B ^a^*	rs4430796	African	1.093 (1.014–1.179) *P* = 0.021	0.000	1.000	3112 vs. 2911 (1)
		Asian	0.849 (0.776–0.929) *P* = 0.000	3.013	0.698	2500 vs. 2379 (6)
		Caucasian	0.816 (0.794–0.838) *P* = 0.000	1.580	0.664	21210 vs. 52279 (4)
*MSMB*	rs10993994	Asian	1.155 (1.052–1.267) *P* = 0.002	1.951	0.377	1627 vs. 2005 (3)
		Caucasian	1.218 (1.175–1.262) *P* = 0.000	2.598	0.273	13087 vs. 12458 (3)

Note that these 20 variants showing positive association in all ‘ethnic groups' were re-meta-analyzed based on different ethnic ancestries.

a positive variants with between-study heterogeneity calculated in all ethnic groups;

b the summary OR and 95% c.i. values.

c Q statistic across crude ORs was calculated for each included study.

d *P* >0.1 is usually considered as an evidence for no between-study heterogeneity; *P* <0.1 as an evidence for significant between-study heterogeneity.

e *HOXB13* rs138213197 and *LEP* rs2167270 only were reported in Caucasian-ancestry. ‘Q statistic=0 and *P=*1’ indicated that a SNV was only investigated once in one ethnic group.

Fifteen of forty-six negative variants (calculation using random-effects model combining all subjects in all ethnic populations) became positive results (*P* value range: 0.04-0.000) in one or two ethnic groups, when re-analyzed them based on different ethnic ancestries. Of these fifteen positive variants, seven (∼47%) were seen only in Asian-ancestry population. Except for one protective variant (*MTHFR* rs1801133) for prostate cancer (OR = 0.684, 95% c.i.=0.565–0.828), six of the seven variant (in *MGMT*, *XRCC1*, *OGG1*, *ERCC2* and *CASC8*) showed risks for prostate cancer. The average risk OR was 1.417 (range: 1.148-1.911), and the mean sample size combining case-control individuals in all Asian-ancestry studies was 2091 (range: 407-3430). Six of the fifteen significant variants were observed in African and Caucasian ancestries, respectively (that is, *RNASEL* rs627928, *CDH1* rs16260 and *PTGS2* rs2745557 in African-ancestry; *MPO* rs2333227, *MDM2* rs2279744 and *THADA* rs1465618 in Caucasian-ancestry). Among the fifteen variants, only one (rs10486567 in *JAZF1*) shared association with prostate cancer in 5,747 African (OR=0.855, 95% c.i.=0.787–0.929) and in 19,461 Caucasian (OR=0.847, 95% c.i.=0.808–0.889) (Figure 1, Supplementary Table S3 and Supplementary Figure S6).

We estimated the potential publication bias of all twenty positive variants in combining all sample sizes across all ethnic groups using Egger's linear regression test. The results suggested that five (*SOD2* rs4880, *ESR1* rs9340799, *VDR* rs1544410, *FOXP4* rs1983891 and *EHBP1* rs721048) showed evidence of significant publication bias (*P* value from 0.048 to 0.01) (Supplementary Figure S7). The results produced by trim and fill algorithm showed that sixteen of the twenty positive variants had a adjusted effect size. The imputed missing studies ranged from 1 to 5 (Supplementary Figure S7).

The statistical power for detection of these twenty significant summary ORs (meta-analyzed in all ethnic groups) ranged from 0.28 to 1, and the average power was 0.77. The power of nine meta-analyses with OR<1.2 was below 80%. The genetic power of five variants (*VDR* rs731236 and *KLK3* rs2735839 with OR <0.8; *SRD5A2* rs9282858, *HOXB13* rs138213197 and *MSMB* rs10993994 with OR > 1.2) approached 80% (Supplementary Table S5).

To estimate the sufficiency and stability of the positive meta-analyses, we performed one-study-removed tests and cumulative meta-analyses (see methods). The results from the one-study-removed test revealed that most of positive meta-analyses (85%) had a slight change in the summary ORs and 95% c.i. values in one direction, but the summary ORs of three variants (*LEP* rs2167270, *LMTK2* rs6465657 and *FAS* rs1800682) became negative when the studies with a large sample size were removed (Supplementary Figure S8). Cumulatively meta-analyzing all twenty positive variants, we found that all meta-analyses achieved consistent and significant effect sizes when an average of two-thirds of the independent studies were accumulated (mean of cumulative OR = 0.83 for protective variants, range:0.72-0.91; mean of cumulative OR=1.29 for risk variants, range:1.04-3.03) (Supplementary Figure S9 and Supplementary Table S6). Four variants (*ESR1* rs9340799, *CYP1B1* rs1056836, *SLC22A3* rs9364554 and *LMTK2* rs6465657) reached significant cumulative ORs until their all samples were included.

## DISCUSSION

As of 1 August, 2015, we collected and extracted data from 728 publications reporting on 217 genetic SNVs in 136 different genes. Based on our inclusion criteria,we systematically assessed the summary ORs of 66 SNVs across 51 different genes using meta-analysis. 35 meta-analyzed SNVs in 32 genes showed statistical significance. 20 of these positive results were derived from the meta-analyses combining all ethnic groups. The other 15 significant SNVs resulted from the analysis procedure in different ethnic ancestries. The average allelic risk summary OR was 1.338, and the average protective summary OR was 0.791.

∼70% (46/66) of SNVs in 371 independent studies involving 418,393 subjects showed no significant association with PCa when combining all samples across all ancestral groups. As we detected the sample size using the medium genotype relative risk of 1.16 that was observed in the present study, α defined as 0.05 and a disease prevalence of 0.1%, the sample size of case subjects ranged from ∼3,500 to 14,500 with minor allelic frequencies between 0.1 and 0.5, when a statistical power approached 80% (estimated using genetic power calculator, see method). According to this estimation, 29 and 44 of 46 negative results could not meet these sample size requirements, respectively. Therefore, the small effect sizes across the current negative meta-analyses might account for the insufficient power.

Meta-analysis is a powerful way not only to measure heterogeneity of associations across independent studies, but also to quantify the extent of between-study heterogeneity [17]. In total, 43 (∼65%) of 66 current meta-analyses showed evidence of significant heterogeneity (*P*<0.1 in Q statistic) across 560 published studies (range of heterogeneity *P*-value: 0.093-0.000). Of them, nine were positive associations and thirty-four were negative associations.

The initial positive study (the first association study) often exhibits an underlying statistical inflation [18]. In our current analyses, we observed the same phenomenon. After excluding the initial study from each of the twenty positive meta-analyses, we found that over half (11/20) of positive meta-analyses showed a reduction in statistical significance. The *P* value decreased, on average, by 9.6-fold (range: 1.429 to 44.214) (Tables 1 and 2, Supplementary Table S2). Four meta-analyses with weak significance became negative results, supported by the evidence from cumulative meta-analysis plots (Supplementary Figure S9). These results suggested that the actual genetic effect of these positive association might be overestimated by the “winner's curse” bias [19].

After removing 37 HWE-deviated studies (including 9 positive associations and 26 negative associations), the heterogeneity values of only nine negative analyses were corrected (heterogeneity *P*-value >0.1). We further excluded the outlier studies from nine positive meta-analyses with significant between-study heterogeneity until reaching homogeneity. Eight of nine could be reduced to display no between-study heterogeneity. We also stratified all studies into the same racial groups and re-performed the meta-analyses. Thirty-three of sixty-six meta-analyses showed positive associations in one or more racial groups. Fifteen negative SNVs identified in the initial meta-analyses became positive (Supplementary Table S3). Eight of forty-three meta-analyses with significant heterogeneity across all ethnic ancestries showed no between-study heterogeneity in ethnic sub-groups (Table 3 and Supplementary Table S3). Notably, thirty-six of sixty-six meta-analyses existed significant between-study heterogeneity (heterogeneity *P*-value <0.1) within a single-ancestry population, suggesting that an evidence of bias for positive results might partly be attributed to undetected population stratification in Asian-, African- and Caucasian-ancestry. Finally, ten initially “negative variants” (in *CASC8*, *CYP1A1*, *CYP3A4*, *GPX1*, *IL18*, *JAZF1*, *MDM2*, *MPO*, *THADA* and *TP53*) under the random-effects model became significant when analyzed using the fixed-effects model (Supplementary Table S7). However, Cochran's Q test suggested that all ten variants have a large between-study heterogeneity (the average *P* value=0.021).

Publication bias tends to occur when small studies with insignificant results are not published. We observed the presence of significant publication bias in five of the current 20 positive meta-analyses using Egger's regression. The bias was further shown by the trim and fill procedure (an average of imputed missing studies was three) (Supplementary Figure S7). These bias evidence suggested that small studies led to larger effects than larger studies for these five positive variants (*FOXP4* rs1983891, *EHBP1* rs721048, *SOD2* rs4880, *ESR1* rs9340799 and *VDR* rs1544410). Although the trim and fill analyses also showed that thirteen positive variants need to impute average missing studies of 2, only small shift of adjusted ORs was observed (shift range: 0.1%-0.7%) (Supplementary Figure S7). Statistical methods used to test publication bias are generally underpowered [20, 21], thus the conclusion of the genetic association of these variants with PCa should be treated cautiously.

Of the twenty positive associations identified here, three significant SNVs (two in *FOXP4* and *LMTK2* with risk effects and one in *RFX6* with protective effects), to our current knowledge, were not reported in previous publications. The vast majority of differences between the results of previously published meta-analyses and the results reported here arose from the different inclusion- and exclusion criteria. For example, five previous meta-analyses included family-based publications. In addition, one difference likely arose from miscalculation in one previous meta-analysis (Supplementary Table S8).

In this meta-analyses, five protein-coding variants (*SRD5A2* p.Ala49Thr, *FGFR4* p.Gly388Arg, *HOXB13* p.Gly84Glu, *CYP1B1* p.Leu432Val and *SOD2* p.Val16Ala) were significantly associated with PCa risk, but the last two variants with small summary ORs (an average of 1.12) had significant between-study heterogeneity (heterogeneity *P*-value <0.1). The rs351855 C>T resides in exon 9 of the *FGFR4* gene leads to a glycine to arginine substitution at codon 388 (p.Gly388Arg). The risk allele (T) of the variant was significantly associated with a poor cancer prognosis [22, 23]. The rs138213197 C>T in *HOXB13*, yielding a rare G84E missense variant, significantly increases the risk of prostate cancer, and the biological function of this 84E remains unclear.

In summary, we have performed a comprehensive estimation of the genetic association between population-based gene-specific SNVs and PCa using currently available data. Our meta-analyses yielded twenty significant associations, but the bulk of their genetic effects were small or modest. Thus, the interpretation of these positive results should be cautious. Further analyses with sufficient power and investigations of the potential biological roles of these genetic variants are needed.

## MATERIALS AND METHODS

### Inclusion criteria

To be considered for inclusion in this meta-analysis, a study must be satisfy four criteria. (i) It must be an association study between a SNV and PCa. Note that in this study, as we exclusively focused on population-based case-control designs to investigate the genetic risk loci of disease, studies based on family designs or quantitative trait analyses were not included in any of the statistical analyses. Studies on microsatellite variants or on genetic markers with complex allelic architecture (for example, SNVs with more than two alleles for which it is difficult to obtain a consistent distribution of alleles and genotypes across different studies) were also not included. As SNVs occurring in known gene sequences can exert severe effects on biological functions, we thus exclusively meta-analyzed gene-specific SNVs here. (ii) The study must be a research published in a peer reviewed journal. (iii) Only studies published in English were included. Because non-English-publishing genetic association papers in PubMed and EMBASE represent 5.6% of all population-based case-control association studies of PCa, the exclusion of these papers is not expected to produce a drastic effect on any of the overall conclusions. (iv) The SNVs were reported on at least three independent case-control studies. For studies with underlying duplicate publications, we contacted the corresponding authors by e-mail to ask for clarification. Those publications that could not be clarified by authors after twice e-mails were considered as duplication publications. We only included the initial publication in our study.

We first performed a search for all publications included in the PubMed database using the keywords “prostate cancer AND (polymorphism OR association OR variation OR variant OR risk OR susceptible OR susceptibility OR sequencing OR case-control OR gene)” between August 1, 1990 and August 1, 2015. A total of 41,955 articles were obtained. The titles and/or abstracts of these papers were scanned for the inclusion criteria above. This step yielded 560 studies containing 66 SNVs in 51 different genes that were eligible for the inclusion criteria in this study. Next, to test the completeness of our search strategies, we searched the EMBASE and Cochrane database using 7 random chosen SNVs or 7 matched genes combined with the keyword “prostate cancer”, respectively.

### Data extraction

First, full-text versions of all papers used for meta-analyses were obtained. Next, the first author, year of publication, ethnic group, genotype or allele data and PubMed ID were extracted from each paper. In this study, ethnic groups encompassed four general populations (African, Asian, Caucasian and Mixed population). All 66 SNVs for subsequent meta-analyses were represented using dbSNP identifiers (“rs” numbers). For the papers in which genotype or allele distributions were not provided, we requested genotype information by directly e-mailing the first or corresponding authors. For SNVs for which we could not obtain the genotype data after two e-mail, genotype distributions were deduced from their allelic data. All information extracted from the publications was summarized in Supplementary Table S1.

### Statistical analyses

SNVs with genotype and/or allele data in three or more independent studies were included for meta-analyses. We performed meta-analyses with i) all studies including general populations (all ethnic groups); ii) studies excluding the initial publication (because the initial publication often shows inflated evidence of association); iii) studies after the exclusion of samples deviating from HWE; iv) studies sorted by subgroups which have same ancestries. We calculated the crude ORs and 95% c.i. values using allele contrasts. Summary ORs and 95% c.i. values were calculated using the Mantel-Haenszel fixed-effects model [24] and the DerSimonian and Laird random-effects model [25]. Statistical heterogeneity across studies was assessed using Cochran's Q test, for which a *P* value< 0.1 showed the presence of significant heterogeneity. For meta-analyses that still showed evidence of between-study heterogeneity (Q statistic, *P* value< 0.1) after excluding deviations from HWE, we performed a heterogeneity correction by the stepwise removal of outlier studies until homogeneity was achieved. We performed one-study-removed analyses to show the effect of each study on the summary OR. In the one-study-removed analysis, a single study was removed from the meta-analysis and the summary ORs and 95% c.i. values of the remaining studies were re-calculated. We estimated publication bias for all statistically significant SNVs. Egger's regression procedure was used to test the extent of publication bias, with a funnel plot to show bias. Duval and Tweedie's trim and fill procedure to impute a shift of ORs and to impute the number of missing studies when a apparent bias was to be removed [26]. We also estimated sufficiency and stability of the positive meta-analyses using meta-cumulative method [27]. In this approach, studies are sorted by the time of publication and summary ORs and 95% c.i. values are calculated iteratively when each study was added. In this study, statistical analyses were conducted using the comprehensive meta-analysis version 2.0 software [Biostat, Englewood, NJ, USA]. Hardy-Weinberg equilibrium was estimated in the control samples and deviation from HWE was defined as a *P* value < 0.05. The statistical power was detected using the Genetic Power Calculator online tool (http://pngu.mgh.harvard.edu/purcell/gpc/cc2.html). The prevalence of PCa was 0.1%. The minor allele frequencies of each SNV in combined cases were used as high-risk allele frequencies, and the genotype relative risks were calculated utilizing the genotype distribution from the combined case-control genotype data and sample size. The type I error rate was 0.05.

## SUPPLEMENTARY FIGURES AND TABLES




























