# Supplementary material for: Systematic meta-analyses of gene-specific genetic association studies in prostate cancer
Source: Oncotarget. 2016 Mar 5;7(16):22271–84. doi: 10.18632/oncotarget.7926 (PMC5008361; doi:10.18632/oncotarget.7926)
Supplement: Supplementary file 1 [file oncotarget-07-22271-s001.pdf]

## SUPPLEMENTARY FIGURES AND TABLES

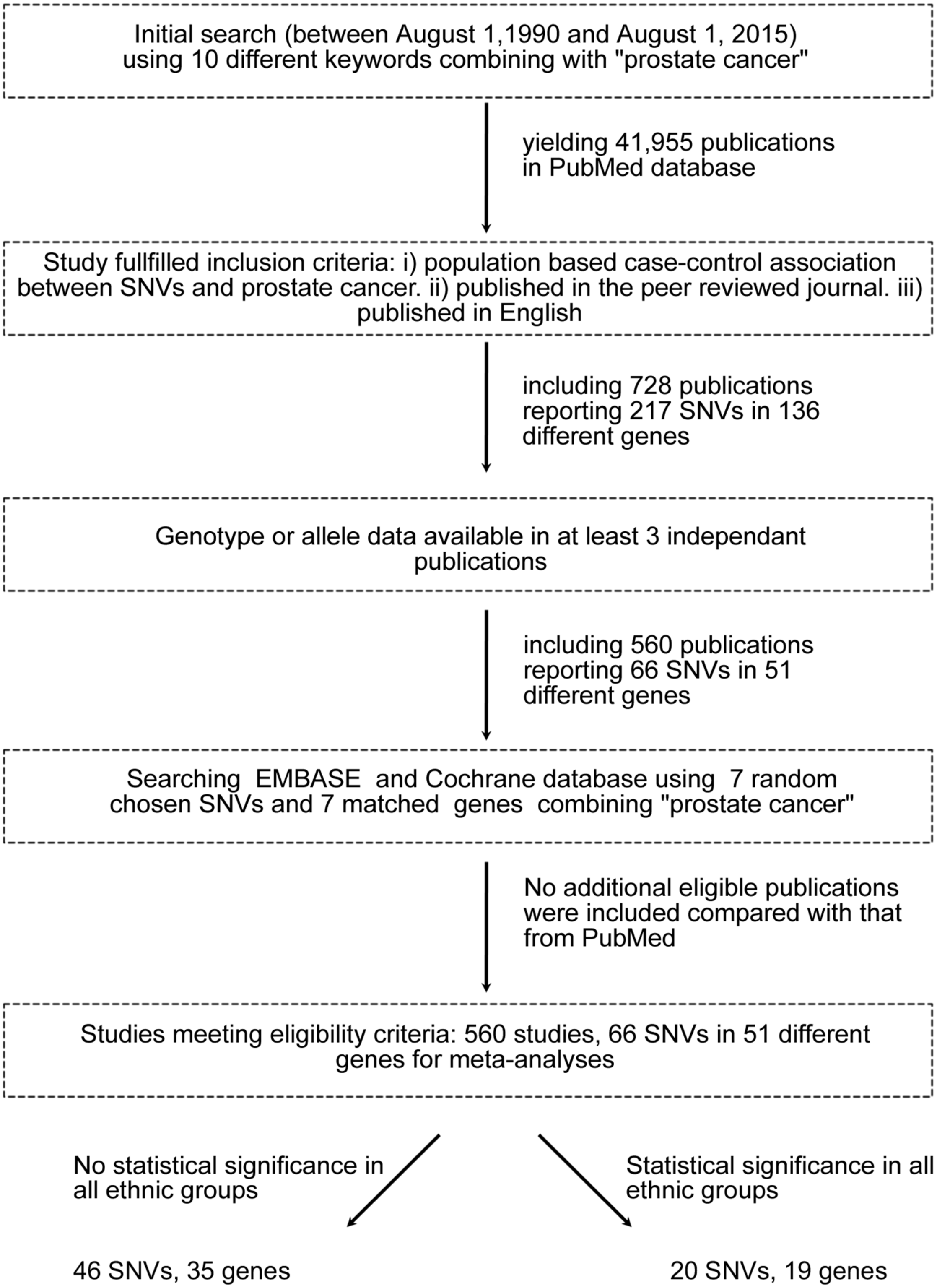


**Supplementary Figure S1: Publications search and flowchart for meta-analyses in this study.**

**Supplementary Figure S2: Graphical display of fixed-effects meta-analyses using allelic contrasts for single nucleotide variants showing significant summary ORs (as of August 1, 2015).** Author's name followed by (a) or (b) et al. represented the same author performed different studies. Summary ORs and 95% c.i. values were calculated using all ethnic populations.

**See Supplementary File 1**

**Supplementary Figure S3: Graphical display of random-effects meta-analyses using allelic contrasts for single nucleotide variants showing significant summary ORs (as of August 1, 2015).** Author's name followed by (a) or (b) or (c) et al. represented the same author performed different studies. Summary ORs and 95% c.i. values were calculated using all ethnic populations.

**See Supplementary File 2**

**Supplementary Figure S4: Graphical display of random-effects meta-analyses using allelic contrasts for single nucleotide variants showing no significant summary ORs (as of August 1, 2015).** Author's name followed by (a) or (b) or (c) et al. represented the same author performed different studies. Summary ORs and 95% c.i. values were calculated with all ethnic populations.

**See Supplementary File 3**

**Supplementary Figure S5: Graphical display of meta-analyses based on ethnic subgroups using allelic contrasts for single nucleotide variants showing significant summary ORs (as of August 1, 2015).** Author's name followed by (a) or (b) or (c) et al. represented the same author performed different studies. Summary ORs and 95% c.i. values were calculated across ethnic subgroups. The Q statistic and *P* values for detection of between-study heterogeneity of each variant see Table 3. *HOXB13* rs138213197 and *LEP* rs2167270 only were reported in Caucasian-ancestry, thus they was not analyzed here.

**See Supplementary File 4**

**Supplementary Figure S6: Graphical display of meta-analyses based on different ethnic populations using allelic contrasts for single nucleotide variants showing no significant summary ORs in all ethnic groups (as of August 1, 2015).** Author's name followed by (a) or (b) or (c) et al. represented the same author performed different studies. Summary ORs and 95% c.i. values were calculated using sorted ethnic subgroups. The Q statistic and *P* values of each variant see Supplementary Table S3.

**See Supplementary File 5**

**Supplementary Figure S7: Funnel plots of the twenty positive meta-analyses in all ethnic groups.** The funnel plots were represented using log odds ratio against precision (the inverse of standard error). Blue cycles and blue filled diamond represented individual studies and summary ORs in the meta-analyses. In the presence of publication bias, the studies will be distributed asymmetrically about the summary OR. Egger's linear regression test was used to quantify the bias with the effect sizes and their precision. The extent of weighted regression of the effect size on its standard error was used to assess bias. Two-tailed *P* value <0.05 was used to show statistic significant. As publication bias was taken into account, Trim and Fill method was used to impute the adjusted ORs and missing studies. Red cycles showed imputed missing studies. Red filled diamond was imputed OR.

**See Supplementary File 6**

**Supplementary Figure S8: The summary of stability of the twenty positive meta-analyses in all ethnic groups using one study removed procedure.** Author's name followed by (a) or (b) or (c) et al. represented the same author performed different studies. The summary OR and 95% c.i. were re-computed when a study is removed from the meta analysis.

**See Supplementary File 7**

**Supplementary Figure S9: The summary of cumulative analyses for the positive meta-analyses in all ethnic groups.** In cumulative analysis, the studies were chronologically sorted, and then the summary OR and 95% c.i. was calculated as a new study was added.

**See Supplementary File 8**

**Supplementary Table S1: A.** Overview of all 66 SNVs had substantial data at least three population-based case-control studies which were meta-analyzed in this study. **B.** 147 SNVs for which data had been reported less than three population based case-control samples did not be meta-analyzed here. **C.** Four polymorphisms could not be analyzed due to more than two alleles.

**See Supplementary File 9**

**Supplementary Table S2: Random-effects meta-analyses using allelic contrasts for SNVs showing non-significant summary ORs (as of August 1, 2015).**

**See Supplementary File 10**

**Supplementary Table S3: Meta-analyses based on sorted ethnic subgroups using allelic contrasts for negative SNVs (as of August 1, 2015).**

**See Supplementary File 11**

**Supplementary Table S4: Heterogeneity correction analyses.**

**See Supplementary File 12**

**Supplementary Table S5: The summary of genetic power tests for the 20 positive variants analyzed by combining all ethnic groups**

| Gene | SNV | Power | OR | Combined sample size [a] |
|---|---|---|---|---|
| *SRD5A2* | rs9282858 | 0.957 | 1.323 | 8256 |
| *FGFR4* | rs351855 | 0.588 | 1.156 | 4775 |
| *VDR* | rs731236 | 0.833 | 0.757 | 2951 |
| *HOXB13* | rs138213197 | 1 | 3.788 | 75277 |
| *FOXP4* | rs1983891 | 0.938 | 1.107 | 24866 |
| *EHBP1* | rs721048 | 0.997 | 1.1 | 66675 |
| *SLC22A3* | rs9364554 | 0.506 | 1.04 | 65960 |
| *MSMB* | rs10993994 | 1 | 1.210 | 29177 |
| *CAT* | rs1001179 | 1 | 1.21 | 32091 |
| *SOD2* | rs4880 | 0.53 | 1.12 | 11117 |
| *KLK3* | rs2735839 | 1 | 0.795 | 37063 |
| *ESR1* | rs9340799 | 0.78 | 1.15 | 8732 |
| *IGFBP3* | rs2854744 | 0.66 | 1.169 | 5808 |
| *CYP1B1* | rs1056836 | 0.71 | 1.129 | 11437 |
| *VDR* | rs1544410 | 0.85 | 0.896 | 15279 |
| *HNF1B* | rs4430796 | 1 | 0.859 | 84391 |
| *RFX6* | rs339331 | 1 | 0.854 | 28535 |
| *LEP* | rs2167270 | 0.32 | 1.163 | 2581 |
| *FAS* | rs1800682 | 0.28 | 0.866 | 2625 |
| *LMTK2* | rs6465657 | 0.45 | 1.060 | 66504 |

Note: a, the total sample sizes combining cases and controls across independent studies.

**Supplementary Table S6: The summary of cumulative meta-analyses of the positive SNVs**

| Gene | SNVs | Cumulative studies (%)[a] | Cumulative ORs | 95% c.i. |
|---|---|---|---|---|
| *CAT* | rs1001179 | 80 | 1.18 | 1.04-1.33 |
| *SOD2* | rs4880 | 93.33 | 1.11 | 1.01-1.22 |
| *KLK3* | rs2735839 | 44.44 | 0.72 | 0.58-0.89 |
| *ESR1* | rs9340799 | 100 | 1.15 | 1.03-1.29 |
| *SRD5A2* | rs9282858 | 76.92 | 1.3 | 1.09-1.56 |
| *FGFR4* | rs351855 | 50 | 1.16 | 1.05-1.28 |
| *IGFBP3* | rs2854744 | 33 | 1.14 | 1.04-1.25 |
| *LEP* | rs2167270 | 66.67 | 1.19 | 1.05-1.34 |
| *CYP1B1* | rs1056836 | 100 | 1.13 | 1.00-1.27 |
| *MSMB* | rs10993994 | 83.33 | 1.22 | 1.18-1.26 |
| *VDR* | rs731236 | 40 | 0.73 | 0.57-0.92 |
| *VDR* | rs1544410 | 66.67 | 0.91 | 0.84-0.99 |
| *HNF1B* | rs4430796 | 63.64 | 0.88 | 0.81-0.97 |
| *HOXB13* | rs138213197 | 42.86 | 3.03 | 1.83-5.02 |
| *FAS* | rs1800682 | 66.67 | 0.87 | 0.77-0.97 |
| *RFX6* | rs339331 | 50 | 0.8 | 0.73-0.87 |
| *FOXP4* | rs1983891 | 33 | 1.11 | 1.04-1.19 |
| *EHBP1* | rs721048 | 33 | 1.14 | 1.06-1.23 |
| *SLC22A3* | rs9364554 | 100 | 1.04 | 1.00-1.08 |
| *LMTK2* | rs6465657 | 100 | 1.06 | 1.03-1.09 |

Note: The cumulative meta-analyses were performed after the studies were chronologically sorted. a, the proportion of cumulative studies in which significant ORs were stably approached.

**Supplementary Table S7: Ten negative SNVs identified by random-effects model became positive results by fixed-effects model**

| Gene | SNV | Model | OR (95% c.i.) *P*-value[a] | *Q*-value | Heterogeneity *P*-value | Cases versus controls (Number of independent samples) |
|---|---|---|---|---|---|---|
| *CASC8* | rs1447295 | A vs. C, random | 1.204 (0.981–1.478) $P = 0.075$ | 100.558 | 0.000 | 6942 vs.5622 (14) |
| | | A vs. C, fixed | 1.254 (1.167–1.347) $P = 0.000$ | | | |
| *CYP1A1* | rs4646903 | C vs. T, random | 1.082 (0.922–1.269) $P = 0.336$ | 19.640 | 0.020 | 2604 vs.2675 (10) |
| | | C vs. T, fixed | 1.120 (1.018–1.233) $P = 0.020$ | | | |

(*Continued*)

| Gene | SNV | Model | OR (95% c.i.) *P*-value[a] | *Q*-value | Heterogeneity *P*-value | Cases versus controls (Number of independent samples) |
|---|---|---|---|---|---|---|
| *CYP3A4* | rs2740574 | G vs. A, random | 1.165 (0.955–1.422) *P*= 0.132 | 26.068 | 0.004 | 1653 vs.1760 (11) |
| | | G vs. A, fixed | 1.169 (1.037–1.319) *P* = 0.011 | | | |
| *GPX1* | rs1050450 | T vs. C, random | 1.165 (0.875–1.552) *P* = 0.296 | 30.429 | 0.000 | 1258 vs.2686 (6) |
| | | T vs. C, fixed | 1.123 (1.011–1.246) *P* = 0.030 | | | |
| *IL18* | rs187238 | G vs. C, random | 0.883 (0.452–1.726) *P* = 0.716 | 33.801 | 0.000 | 931 vs.971 (3) |
| | | G vs. C, fixed | 0.826 (0.704–0.970) *P* = 0.020 | | | |
| *JAZF1* | rs10486567 | A vs. G, random | 0.951(0.828–1.093) *P* = 0.477 | 21.972 | 0.000 | 14800 vs.13433 (5) |
| | | A vs. G, fixed | 0.872 (0.838–0.907) *P* = 0.000 | | | |
| *MDM2* | rs2279744 | G vs. T, random | 0.841 (0.680–1.040) P = 0.109 | 6.411 | 0.093 | 732 vs. 836 (4) |
| | | G vs. T, fixed | 0.851 (0.737–0.983) *P* = 0.028 | | | |
| *MPO* | rs2333227 | A vs. G, random | 0.790 (0.606–1.029) *P* = 0.080 | 4.819 | 0.090 | 762 vs.1678 (3) |
| | | A vs. G, fixed | 0.839 (0.725–0.971) *P* = 0.018 | | | |
| *THADA* | rs1465618 | A vs. G, random | 0.921(0.726–1.168) *P* = 0.498 | 22.730 | 0.000 | 11151 vs.11429 (3) |
| | | A vs. G, fixed | 1.074(1.027–1.122) *P* = 0.002 | | | |
| *TP53* | rs1042522 | G vs. C, random | 0.811 (0.605–1.087) *P* = 0.161 | 58.807 | 0.000 | 1274 vs.1543 (10) |
| | | G vs. C, fixed | 0.853 (0.763–0.953) *P* = 0.005 | | | |

Note: a, summary OR and 95% c.i. were calculated with all samples in all ethnic groups

**Supplementary Table S8: The comparison of results between previously published meta-analysis results and this meta-analyses.**

**See Supplementary File 13**
