# Supplementary material for: Systematic meta-analyses of gene-specific genetic association studies in prostate cancer
Source: Oncotarget. 2016 Mar 5;7(16):22271–84. doi: 10.18632/oncotarget.7926 (PMC5008361; doi:10.18632/oncotarget.7926)

**Supplementary Figure 2** Graphical display of fixed-effects meta-analyses using allelic contrasts for single nucleotide variants showing significant summary ORs (as of August 1, 2015). Author's name followed by (a) or (b) et al. represented the same author performed different studies. Summary ORs and 95% c.i. values were calculated using all ethnic populations.


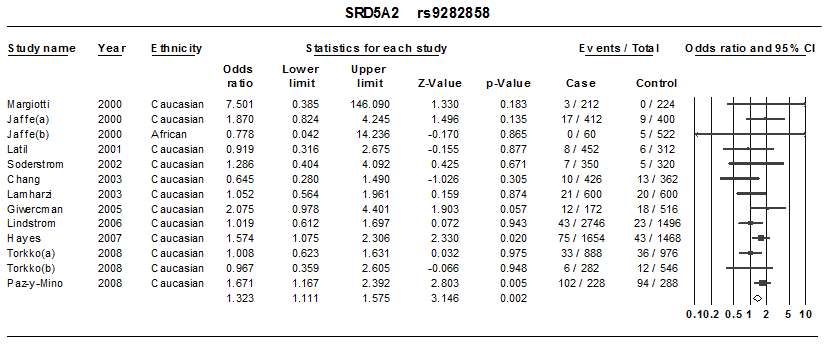

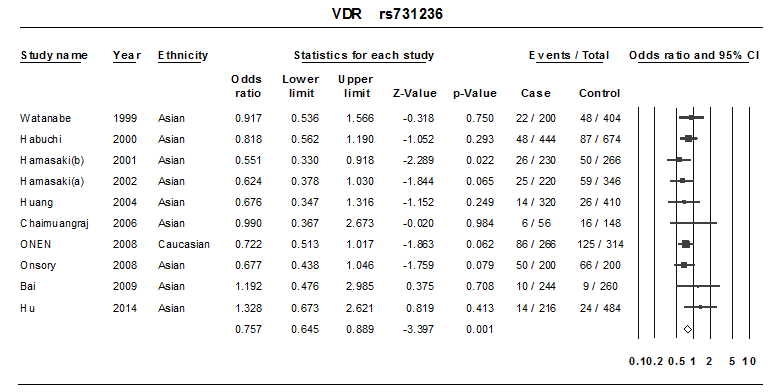


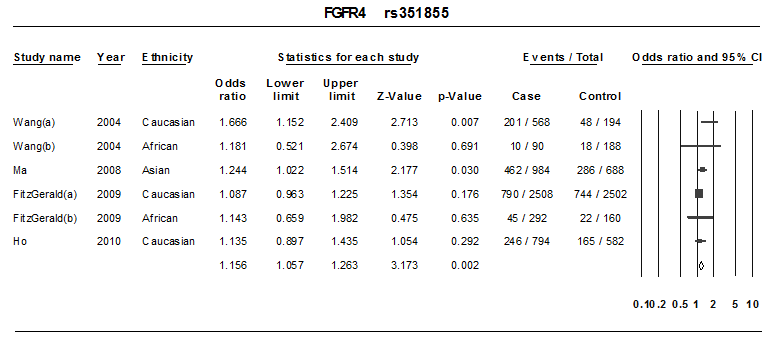


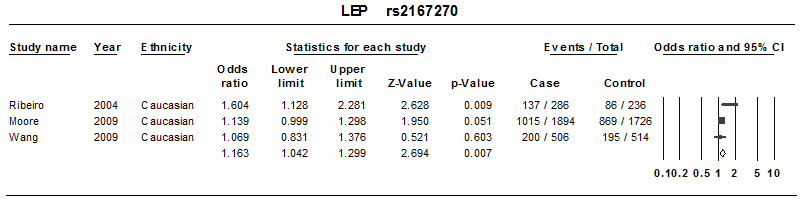


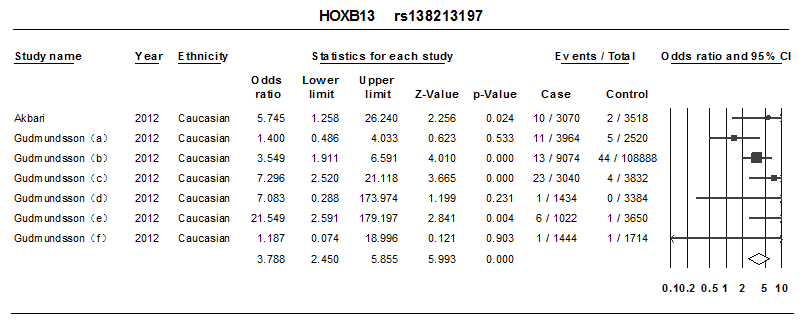


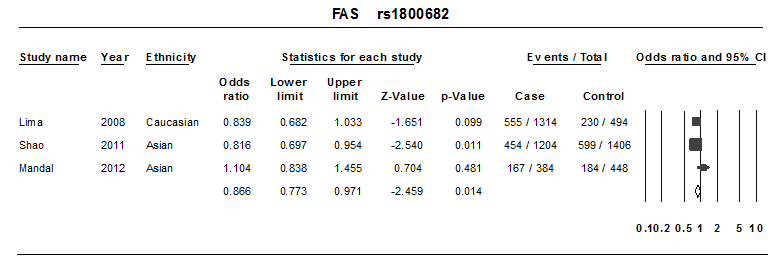


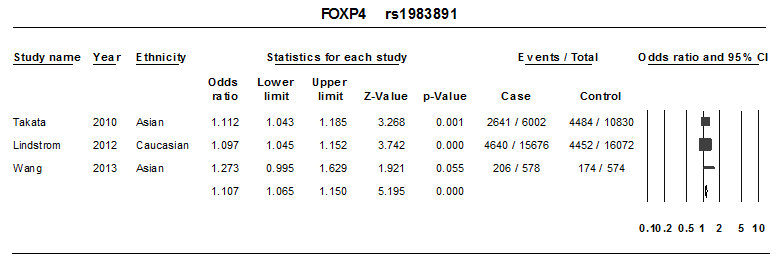


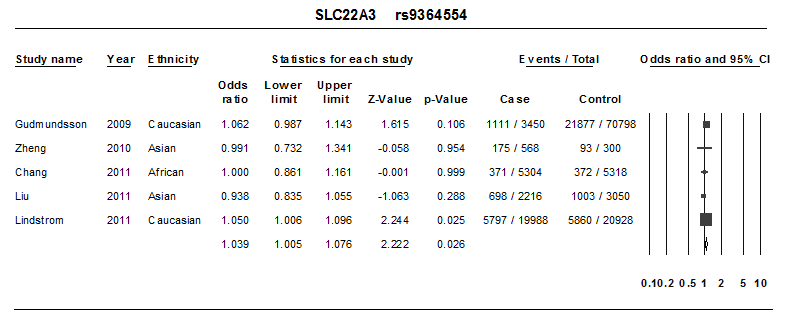


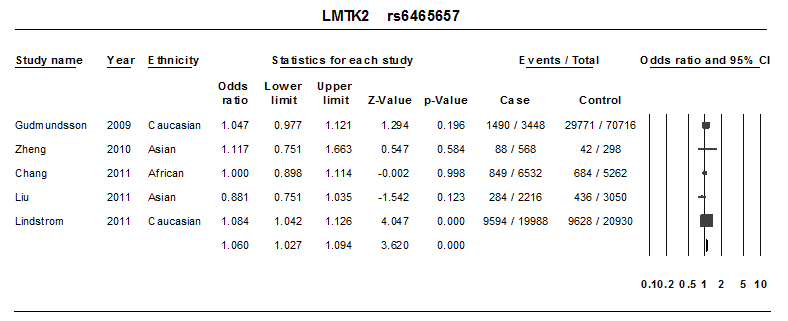


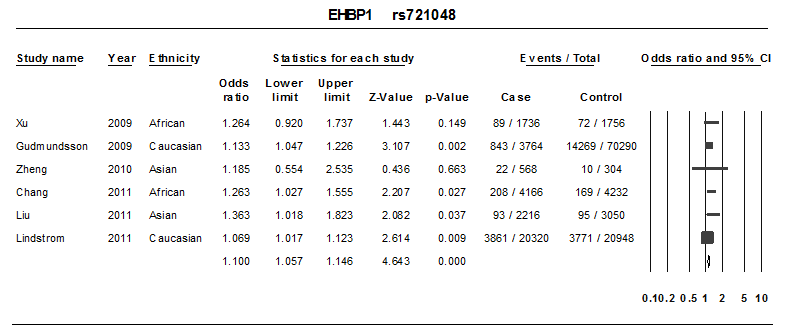


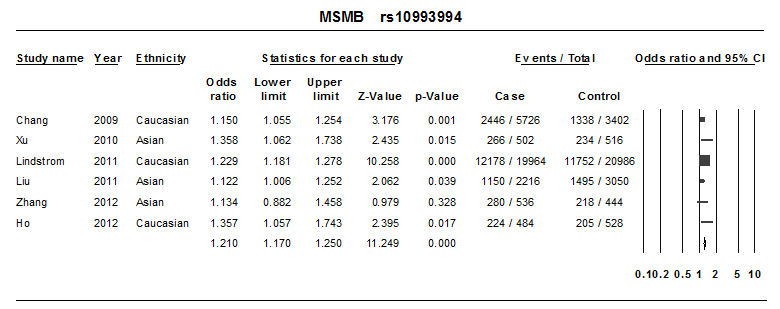

Supplement: Supplementary file 2 [file oncotarget-07-22271-s002.docx]
