# Supplementary material for: Systematic meta-analyses of gene-specific genetic association studies in prostate cancer
Source: Oncotarget. 2016 Mar 5;7(16):22271–84. doi: 10.18632/oncotarget.7926 (PMC5008361; doi:10.18632/oncotarget.7926)

**Supplementary Figure 5** Graphical display of meta-analyses based on ethnic subgroups using allelic contrasts for single nucleotide variants showing significant summary ORs (as of August 1, 2015). Author's name followed by (a) or (b) or (c) et al. represented the same author performed different studies. Summary ORs and 95% c.i. values were calculated across ethnic subgroups. The Q statistic and *P* values for detection of between-study heterogeneity of each variant see Table 3. *HOXB13* rs138213197 and *LEP* rs2167270 only were reported in Caucasian-ancestry, thus they was not analyzed here.


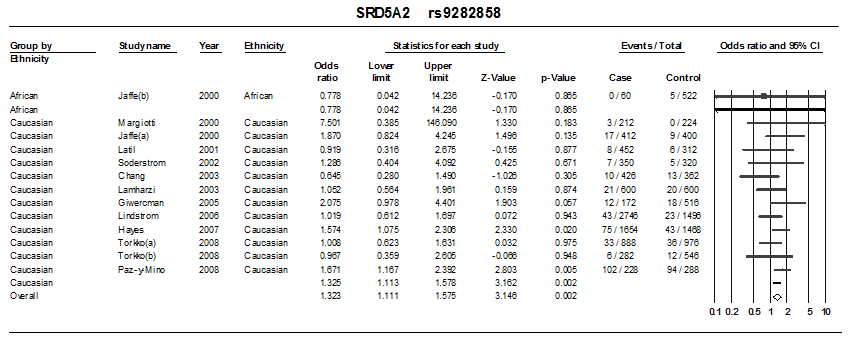


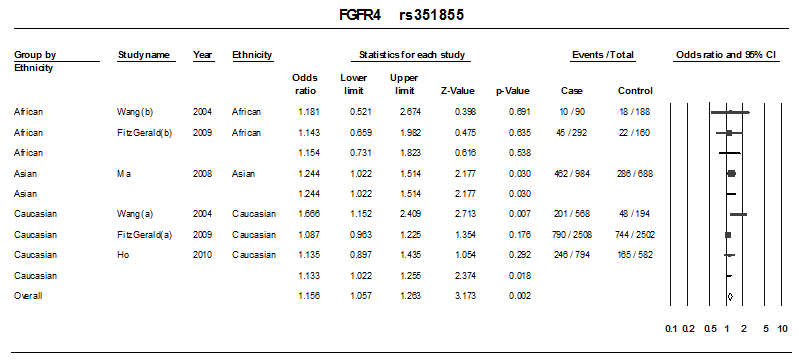


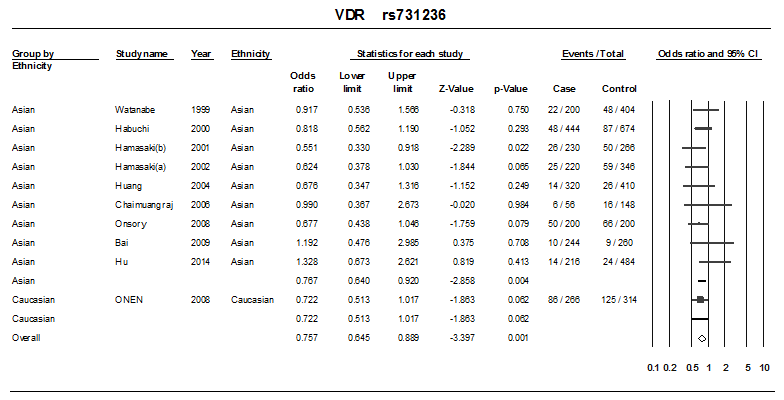


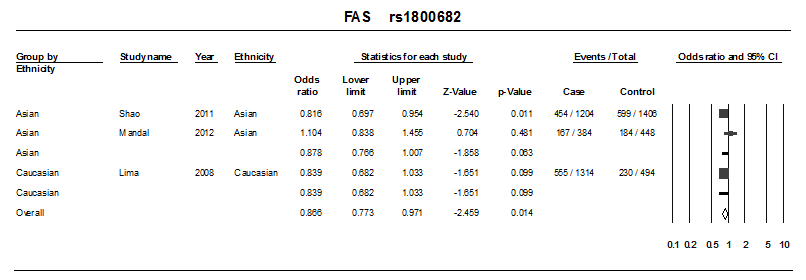


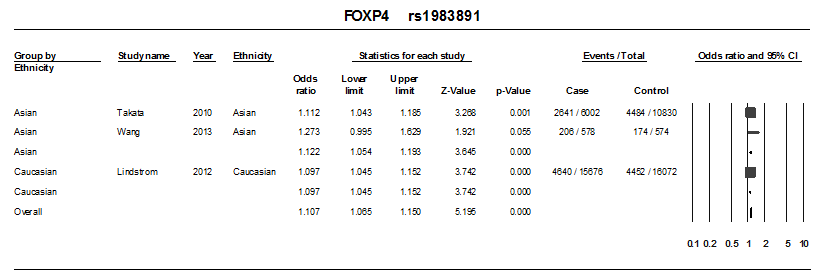


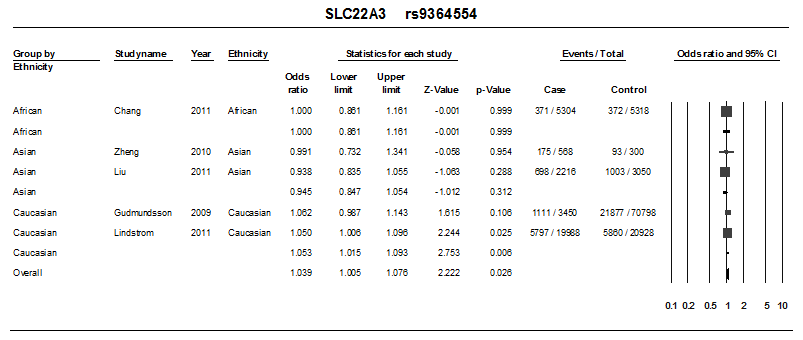


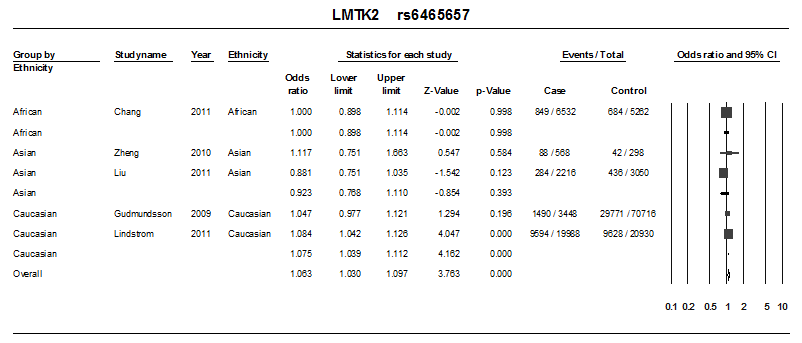


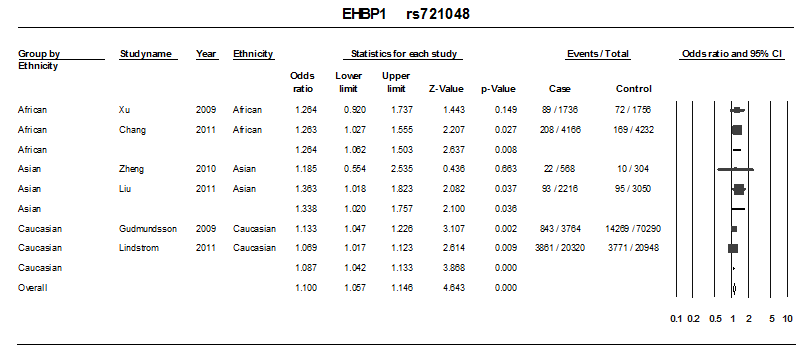


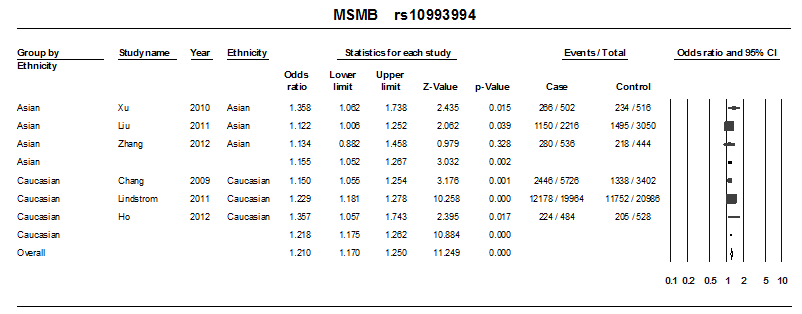


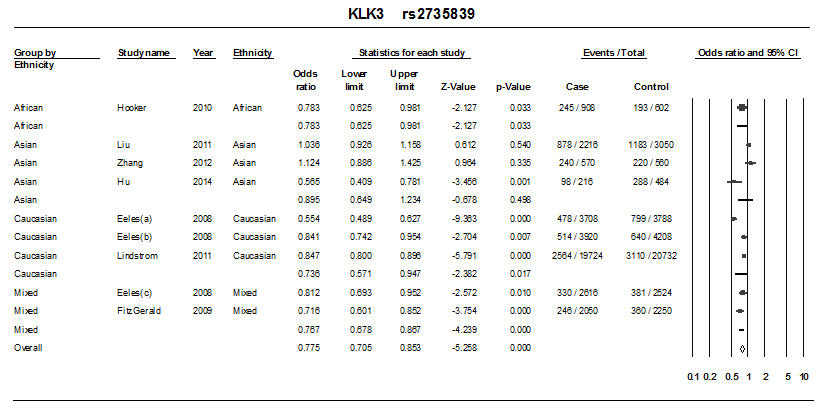


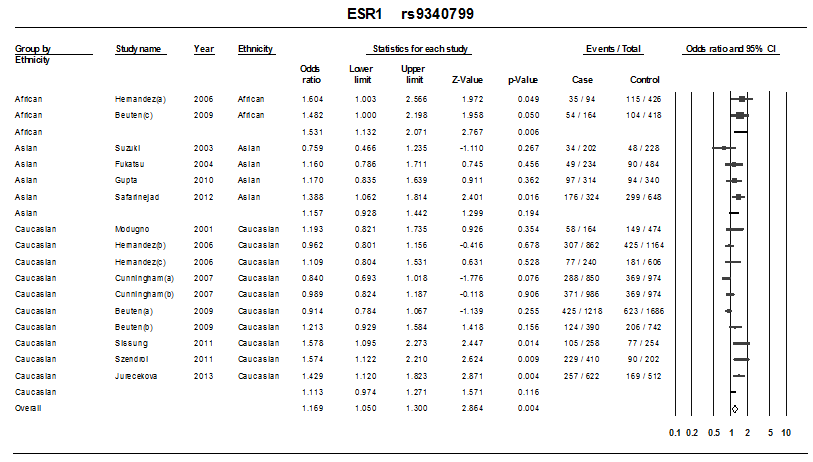


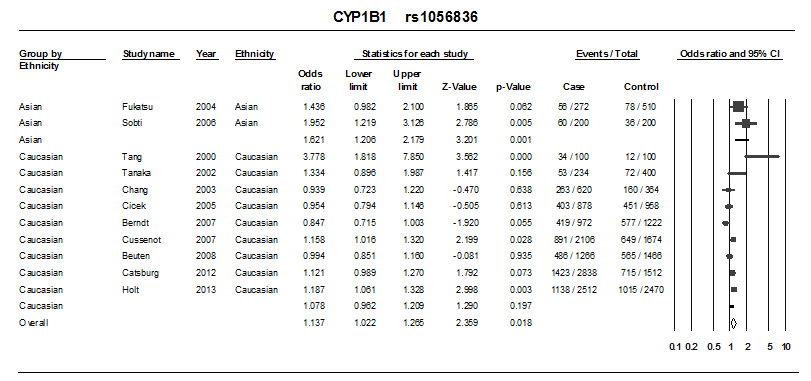


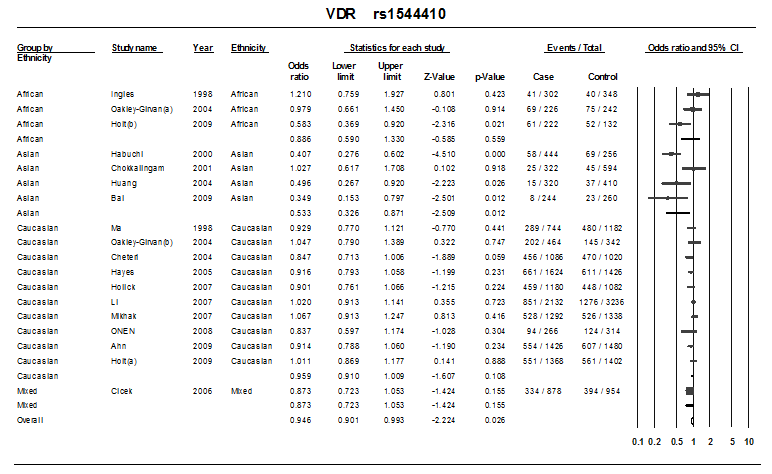


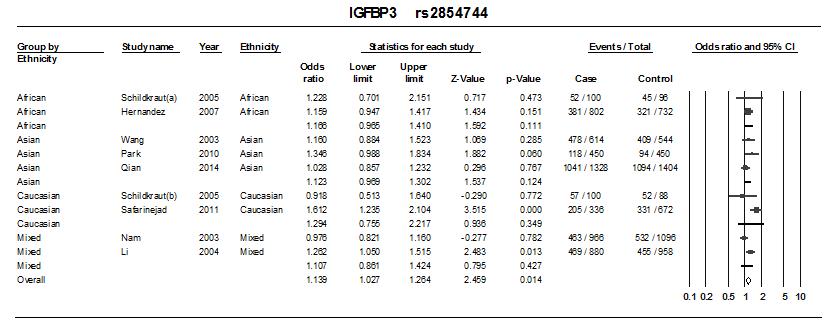


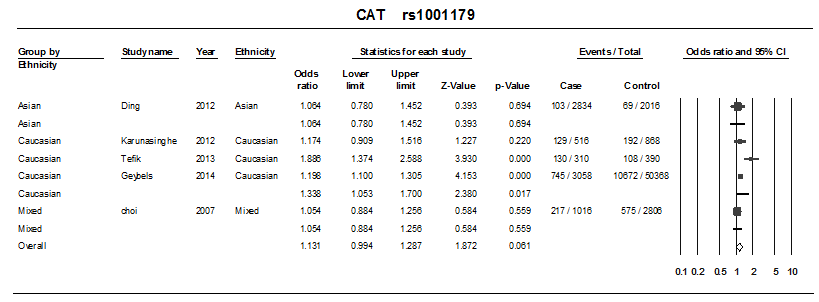


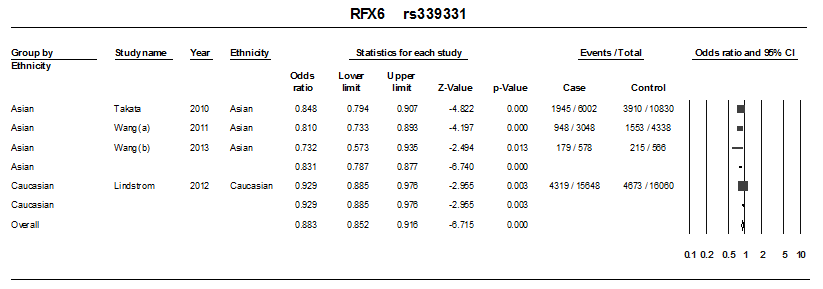


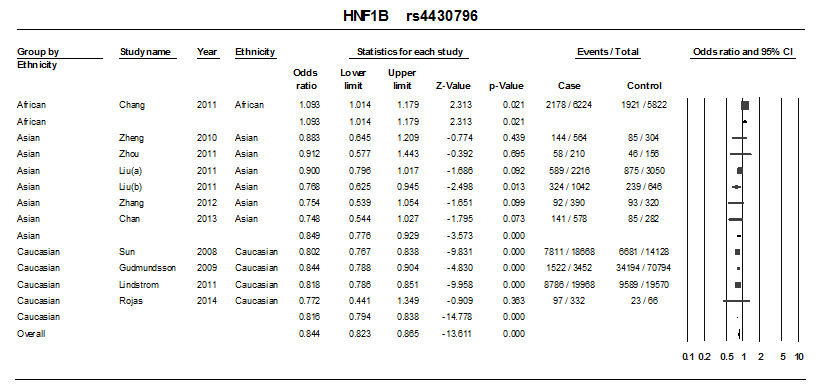


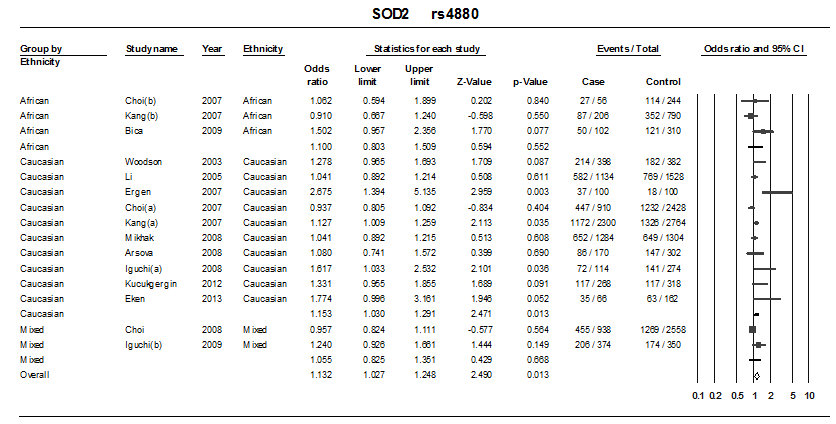

Supplement: Supplementary file 5 [file oncotarget-07-22271-s005.docx]
