# Supplementary material for: Systematic meta-analyses of gene-specific genetic association studies in prostate cancer
Source: Oncotarget. 2016 Mar 5;7(16):22271–84. doi: 10.18632/oncotarget.7926 (PMC5008361; doi:10.18632/oncotarget.7926)

**Supplementary Figure 6** Graphical display of meta-analyses based on different ethnic populations using allelic contrasts for single nucleotide variants showing no significant summary ORs in all ethnic groups (as of August 1, 2015). Author's name followed by (a) or (b) or (c) et al. represented the same author performed different studies. Summary ORs and 95% c.i. values were calculated using sorted ethnic subgroups. The Q statistic and *P* values of each variant see Supplementary Table 3.


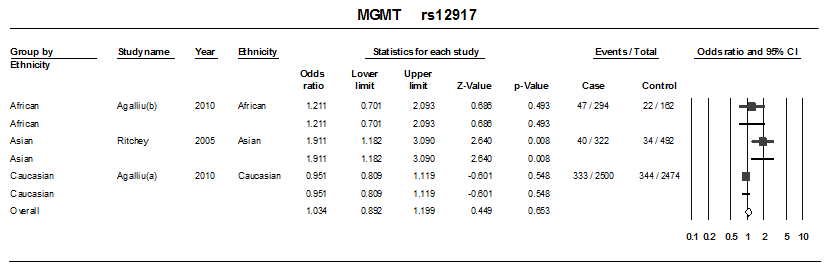


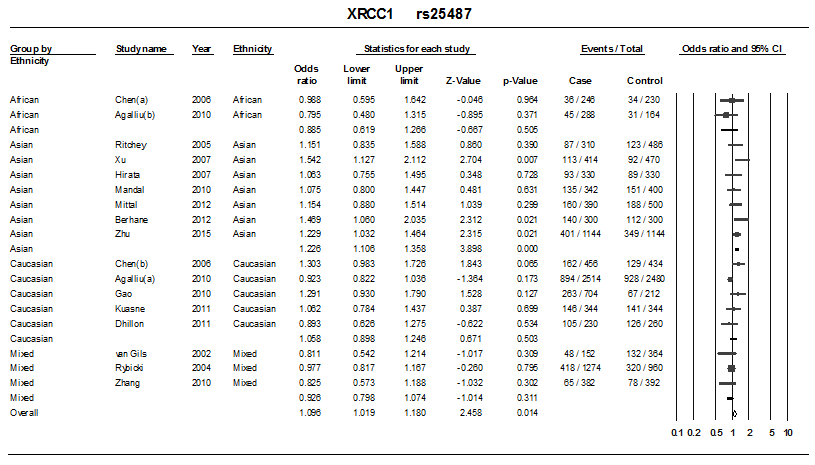


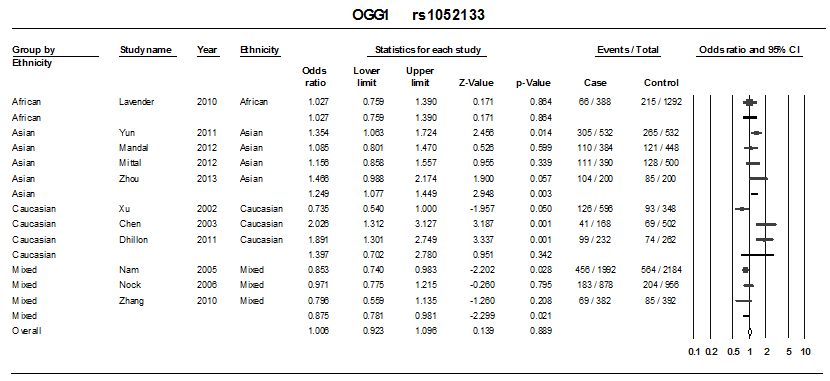


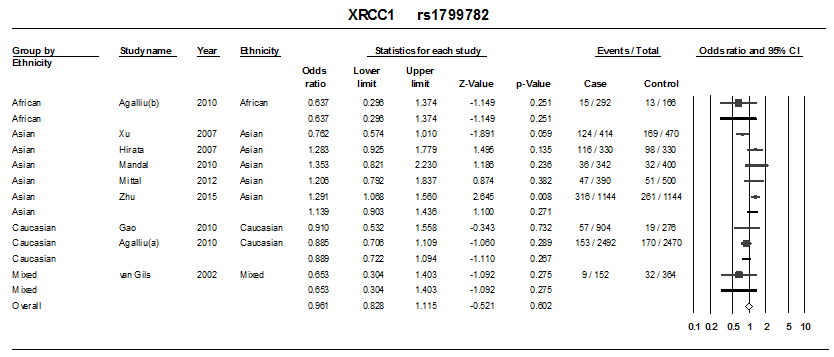


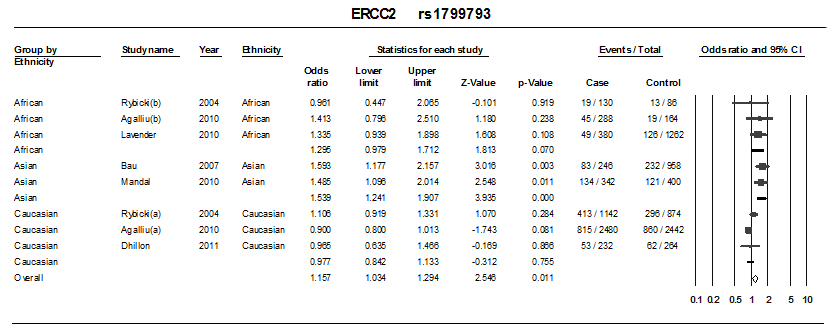


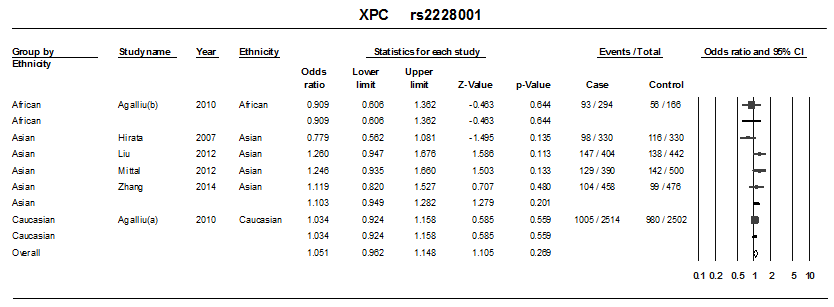


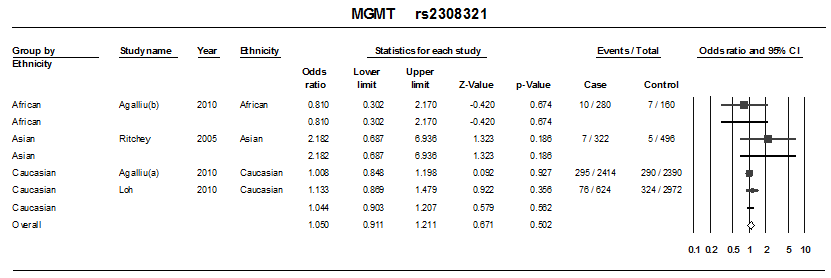


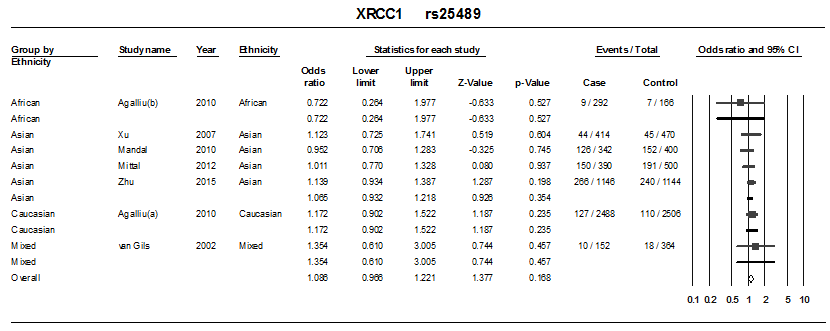


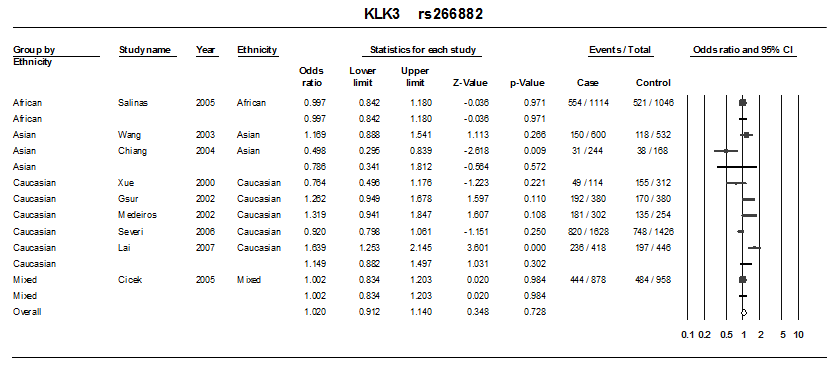


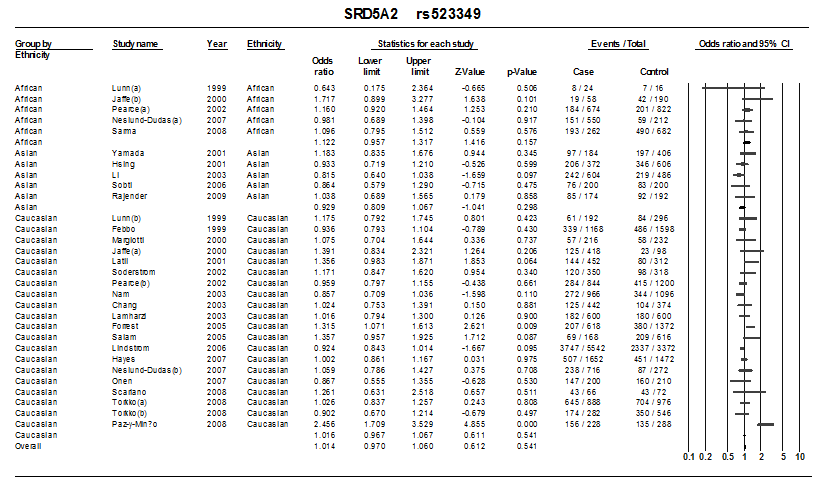


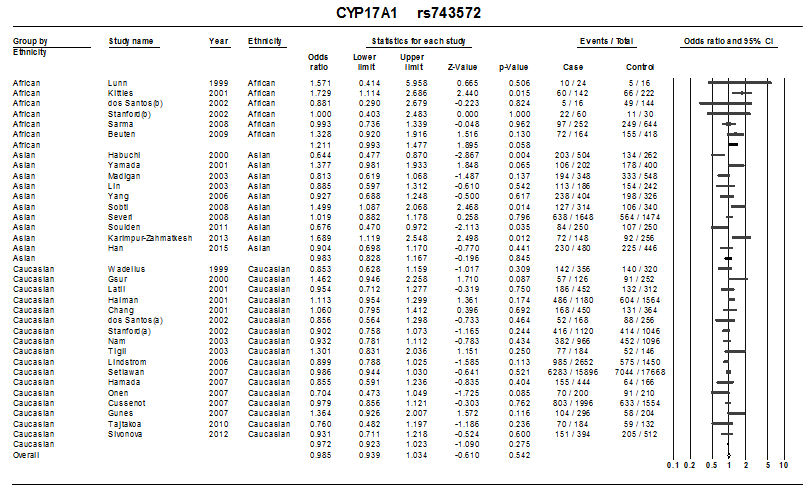


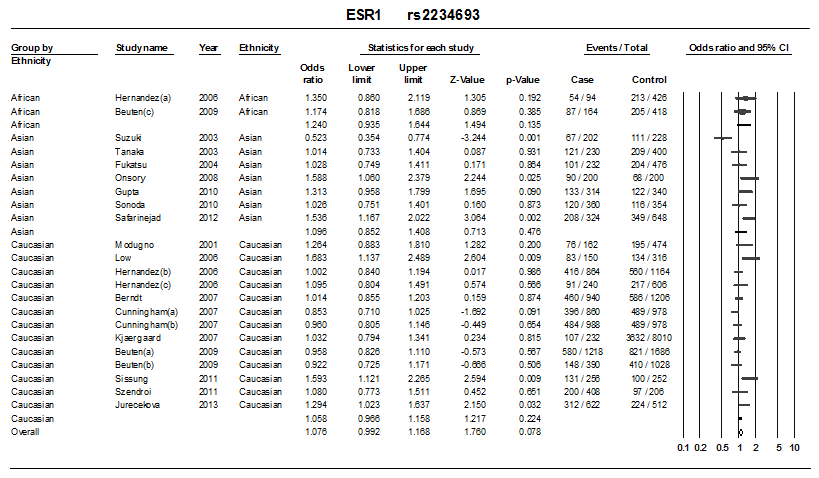


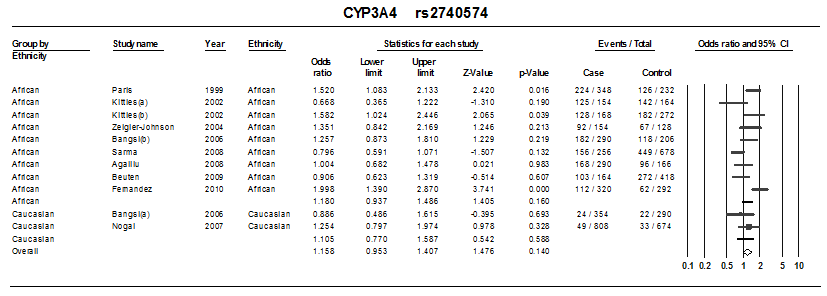


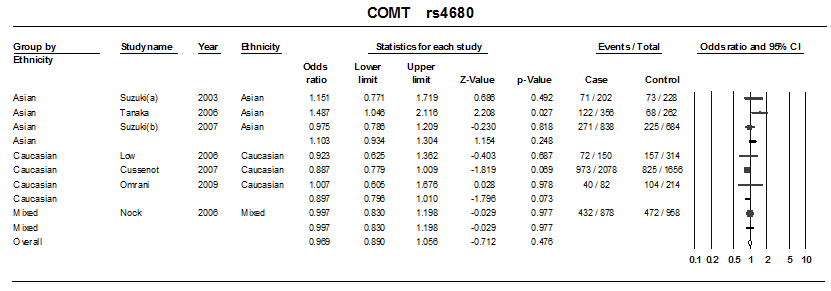


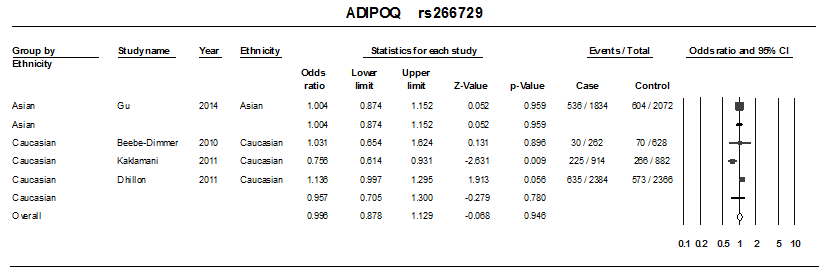


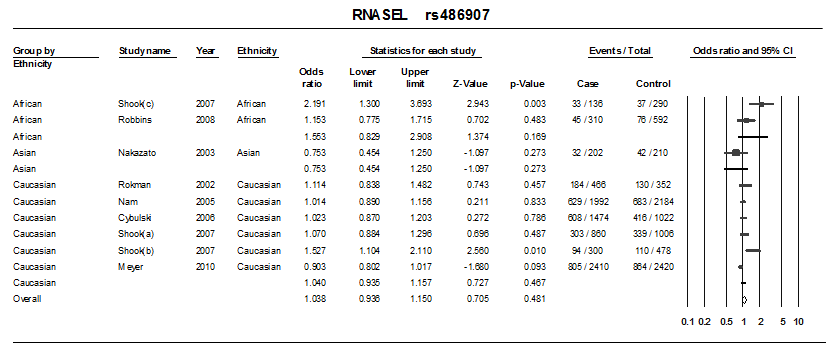


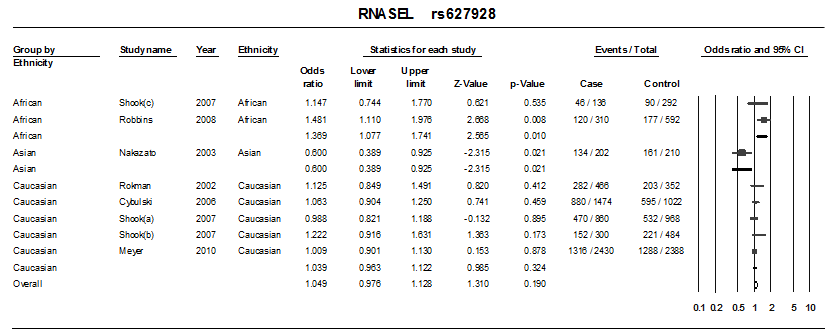


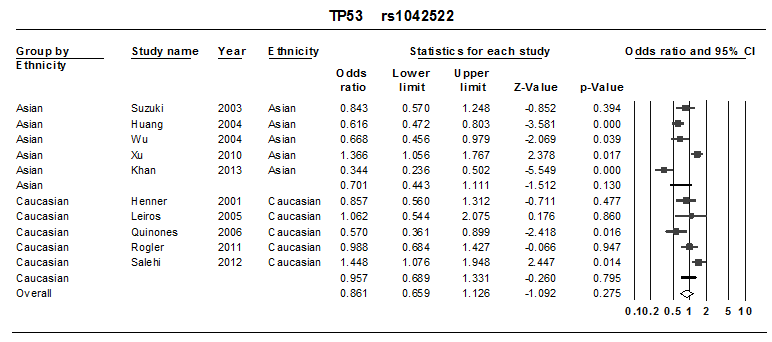


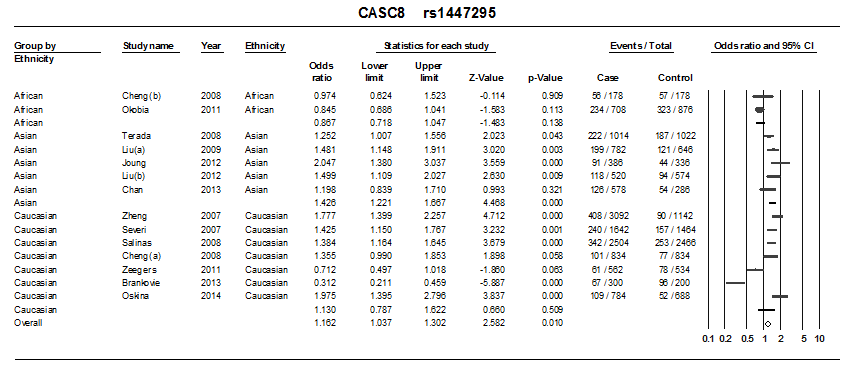


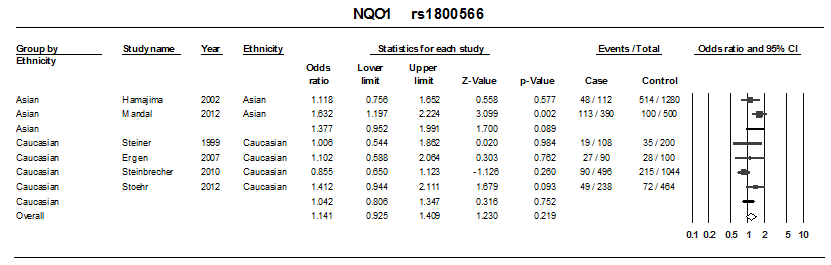


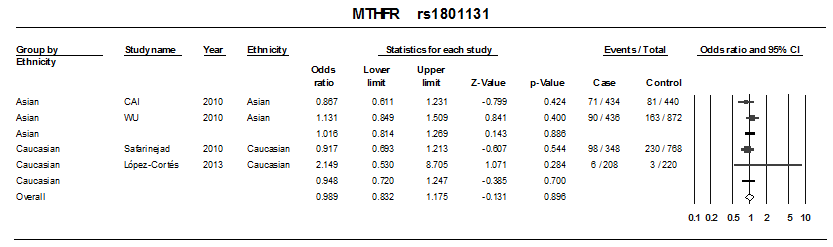


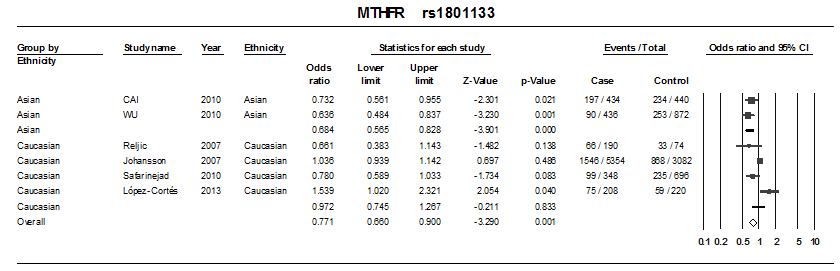


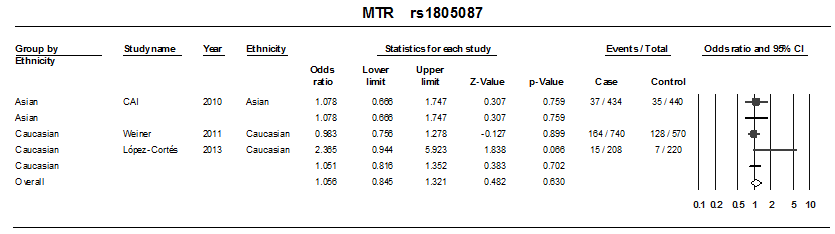


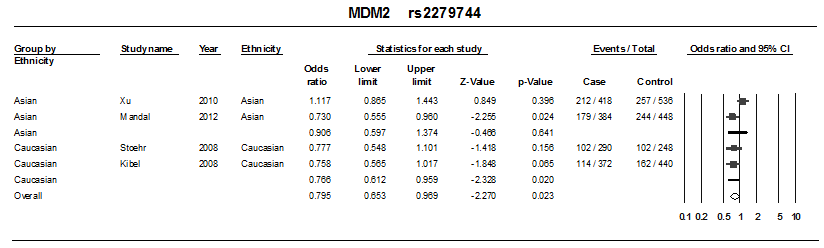


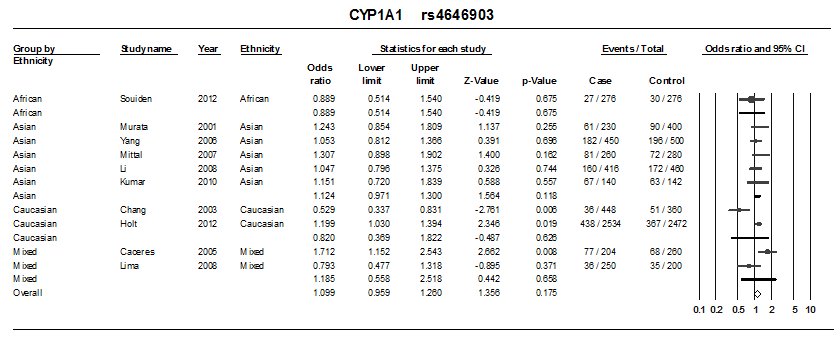


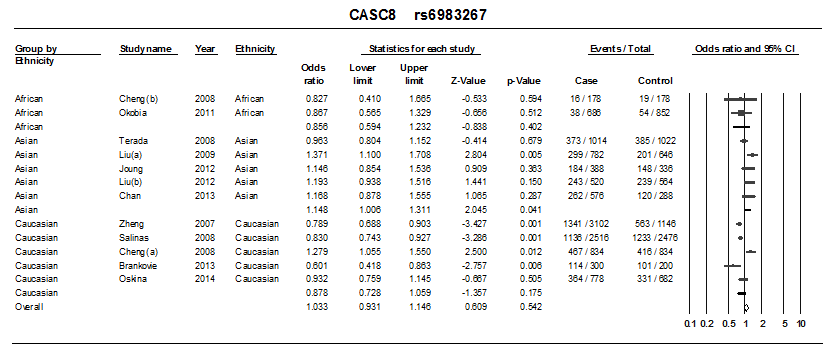


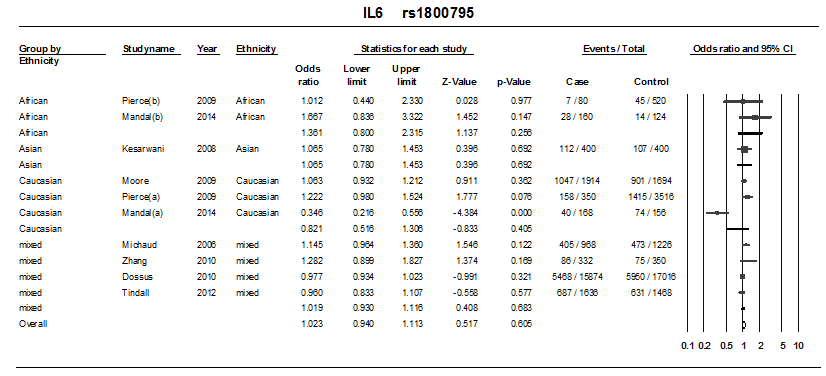


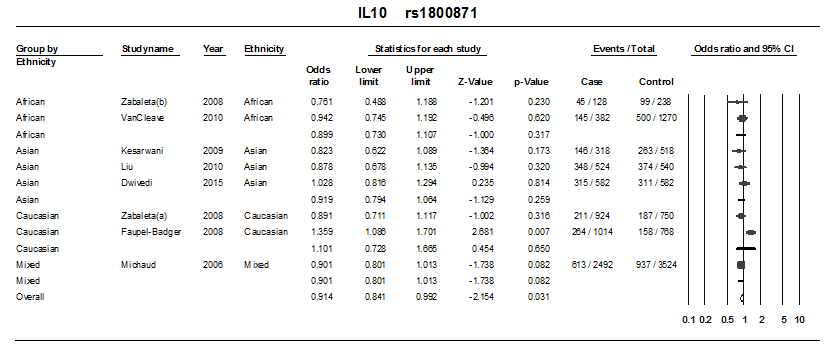


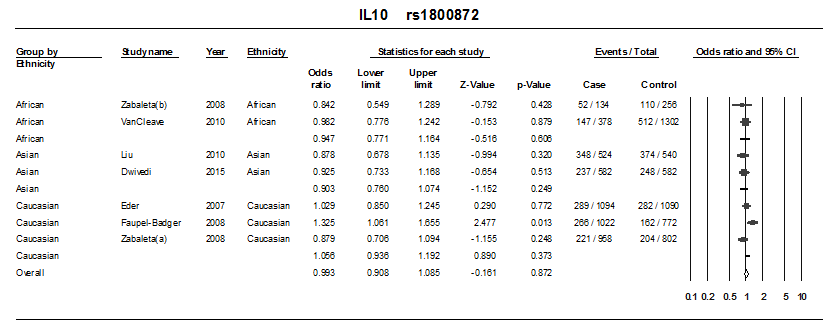


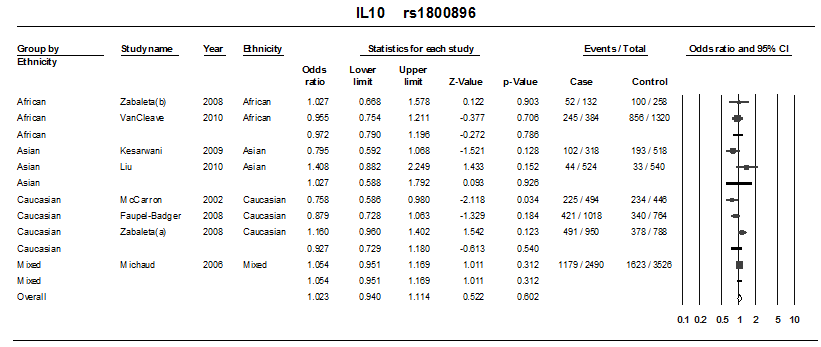


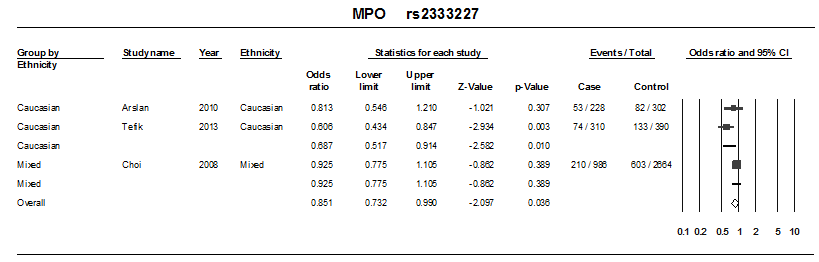


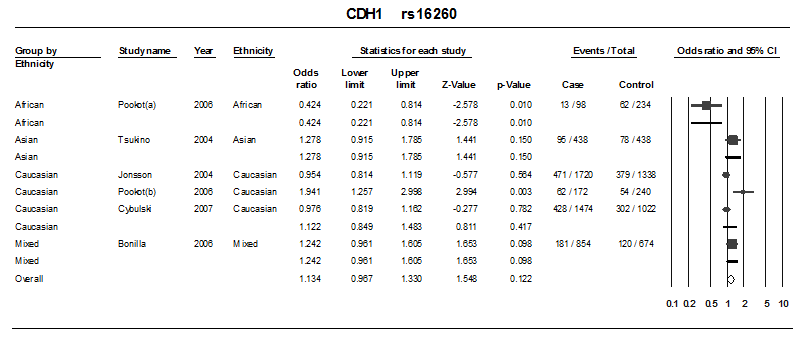


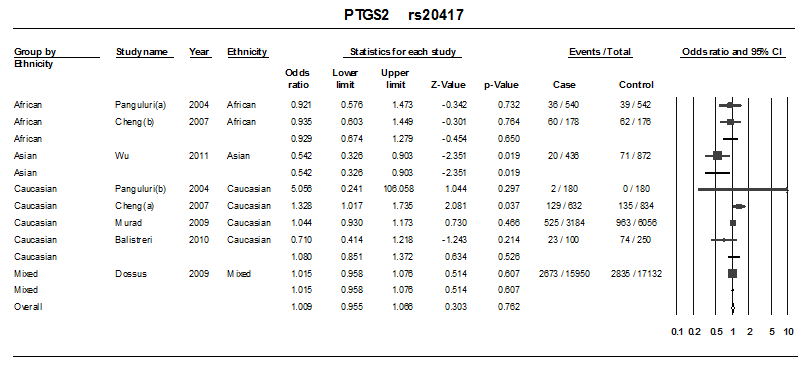


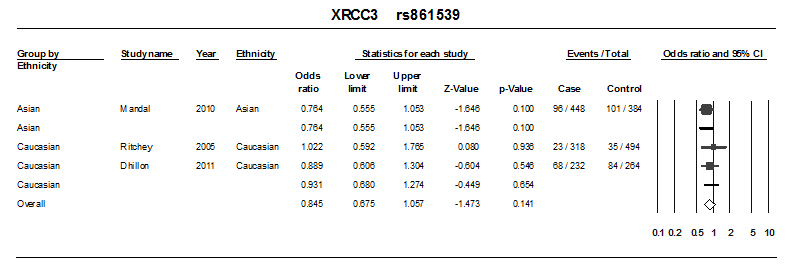


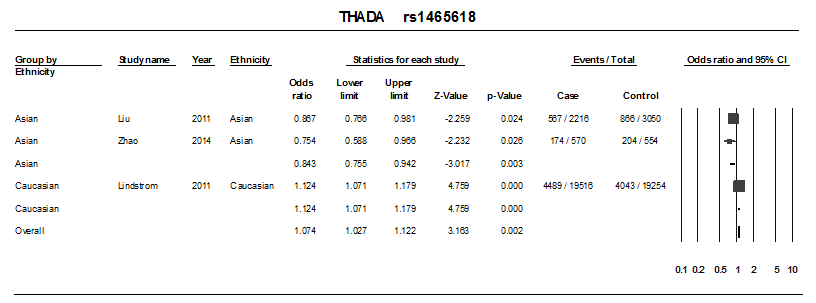


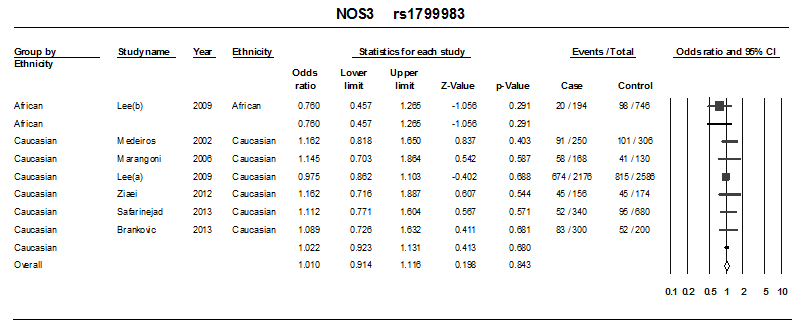


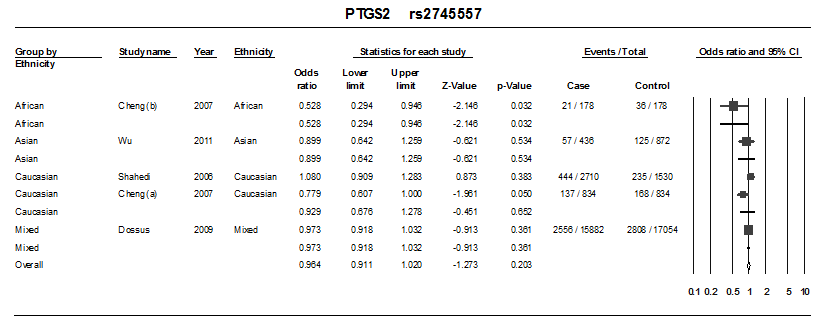


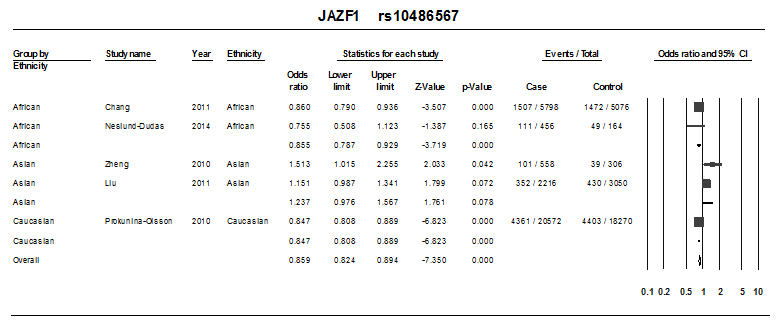


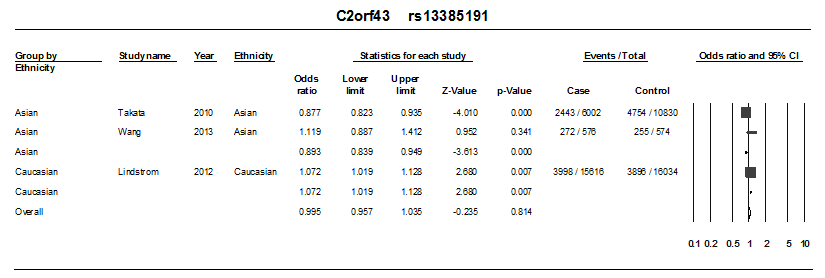


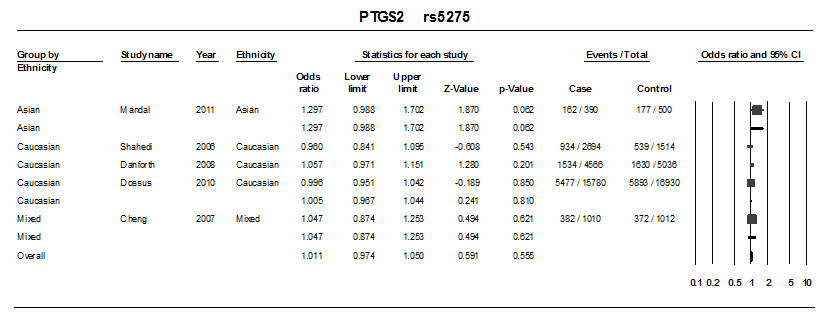

Supplement: Supplementary file 6 [file oncotarget-07-22271-s006.docx]
