# Supplementary material for: Systematic meta-analyses of gene-specific genetic association studies in prostate cancer
Source: Oncotarget. 2016 Mar 5;7(16):22271–84. doi: 10.18632/oncotarget.7926 (PMC5008361; doi:10.18632/oncotarget.7926)

**Supplementary Figure 7** funnel plots of the twenty positive meta-analyses in all ethnic groups. The funnel plots were represented using log odds ratio against precision (the inverse of standard error). Blue cycles and blue filled diamond represented individual studies and summary ORs in the meta-analyses. In the presence of publication bias, the studies will be distributed asymmetrically about the summary OR. Egger’s linear regression test was used to quantify the bias with the effect sizes and their precision. The extent of weighted regression of the effect size on its standard error was used to assess bias. Two-tailed *P* value <0.05 was used to show statistic significant. As publication bias was taken into account, Trim and Fill method was used to impute the adjusted ORs and missing studies. Red cycles showed imputed missing studies. Red filled diamond was imputed OR.


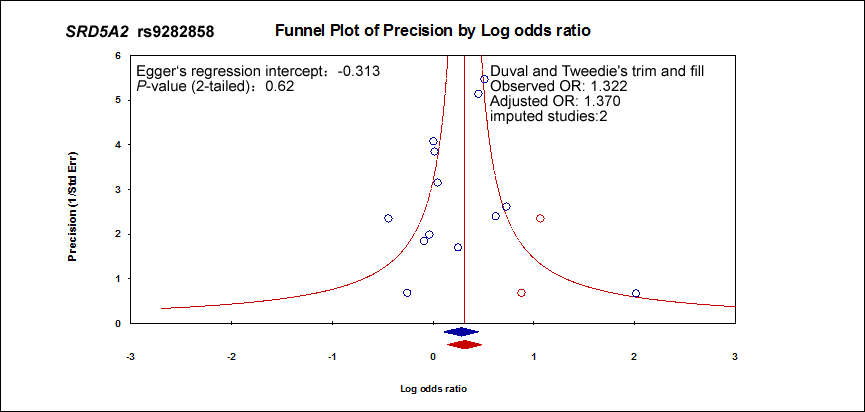


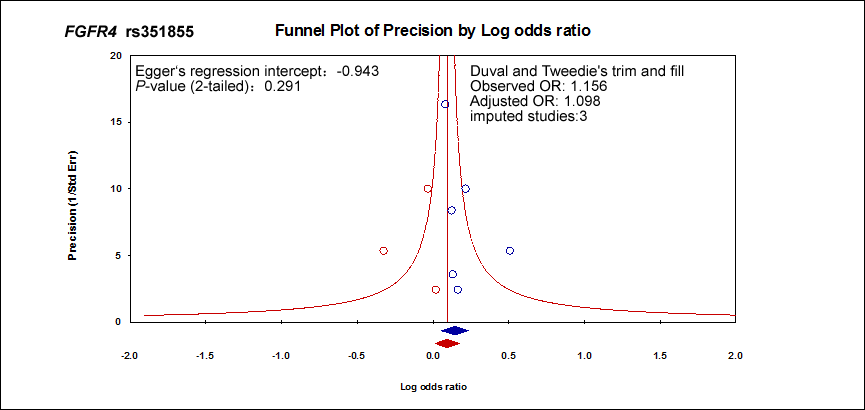


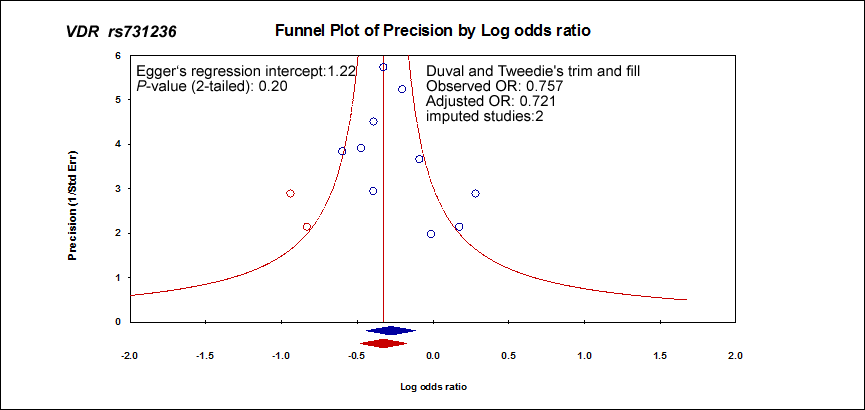


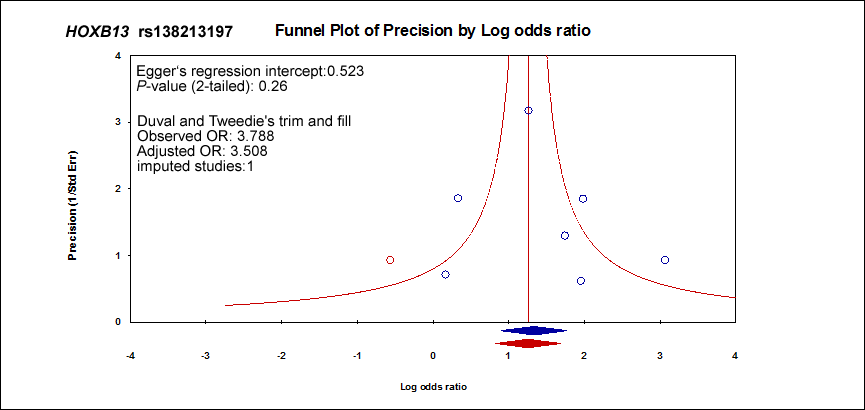


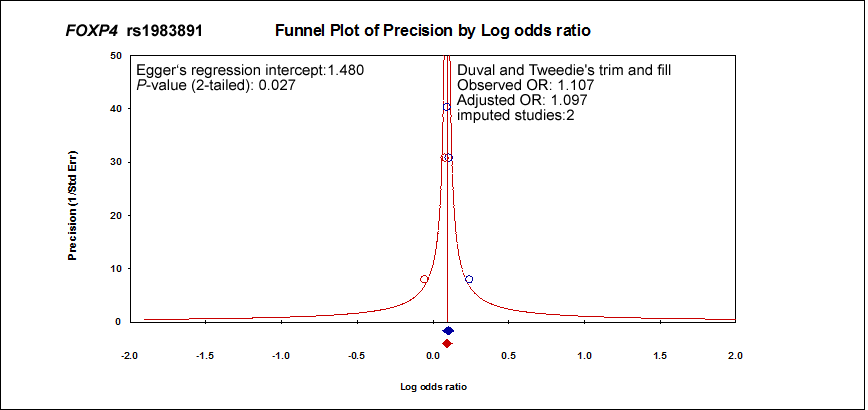


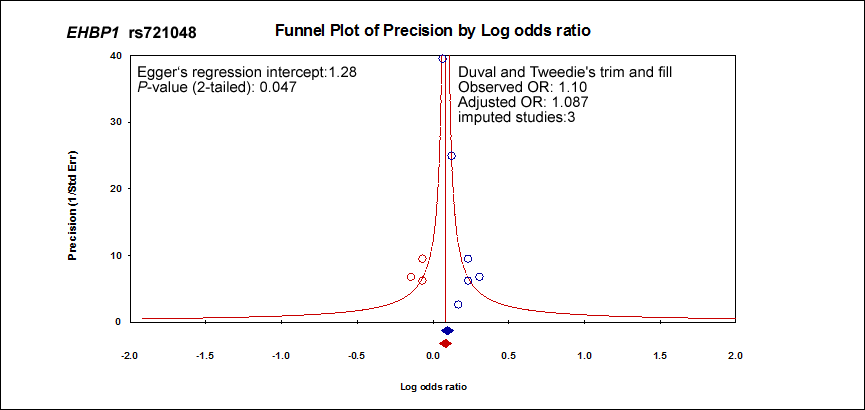


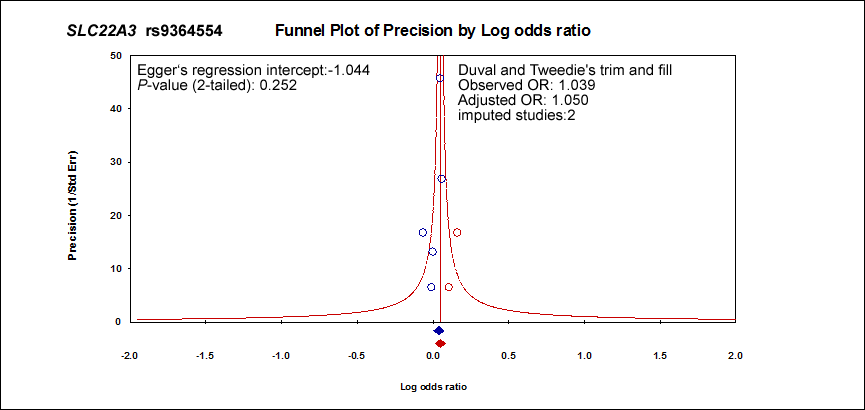


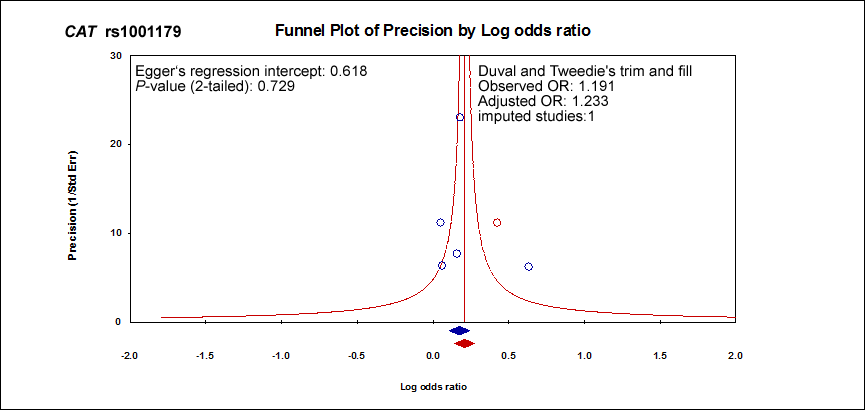


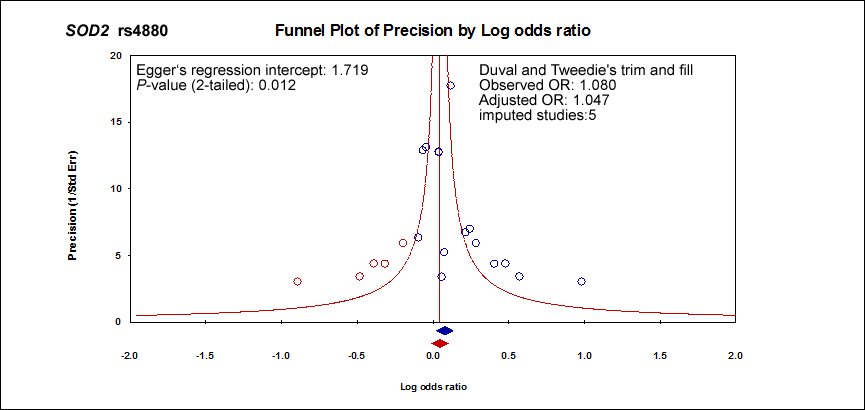


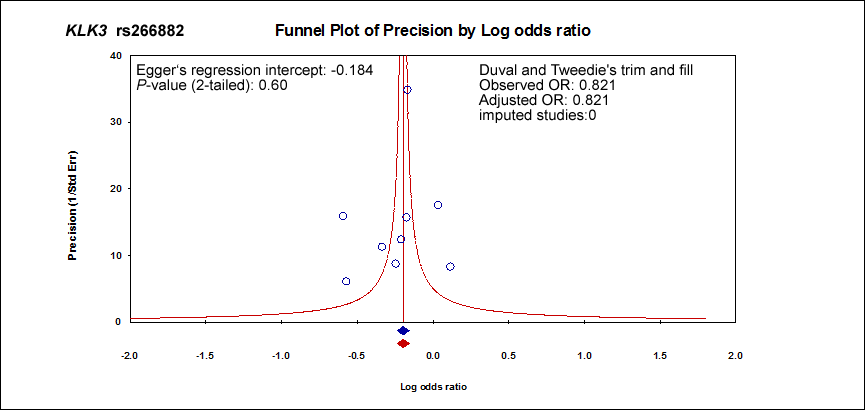


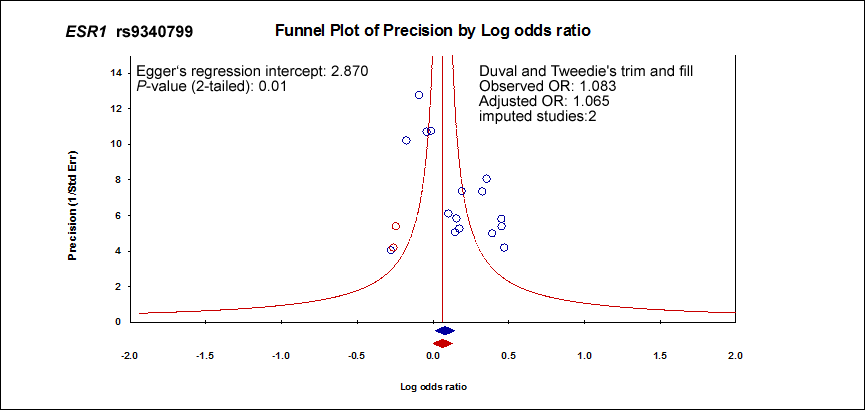


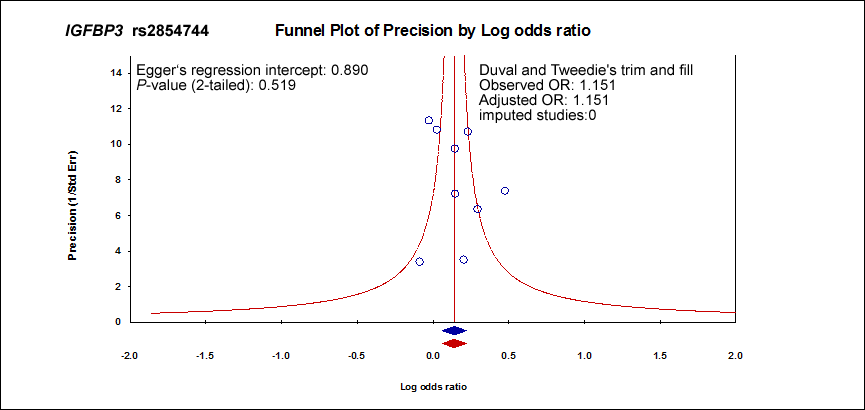


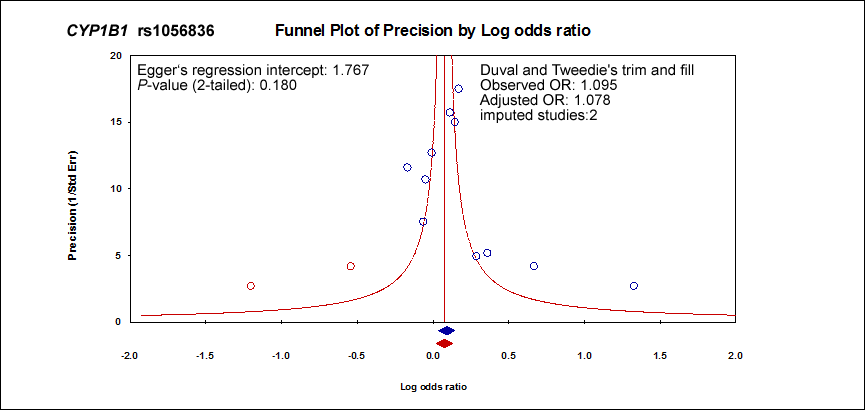


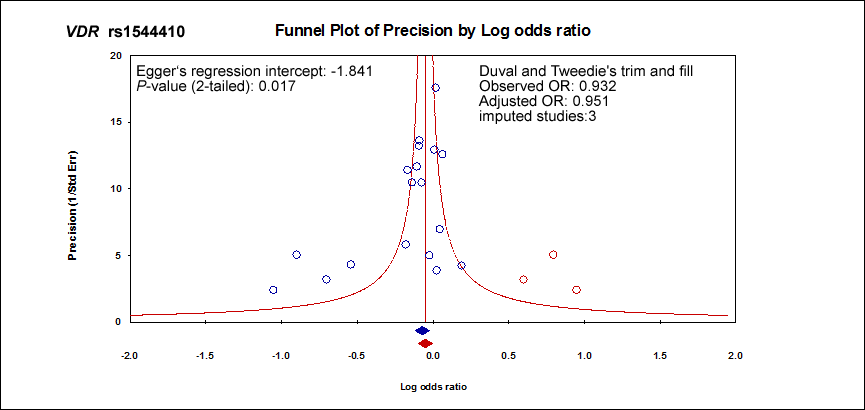


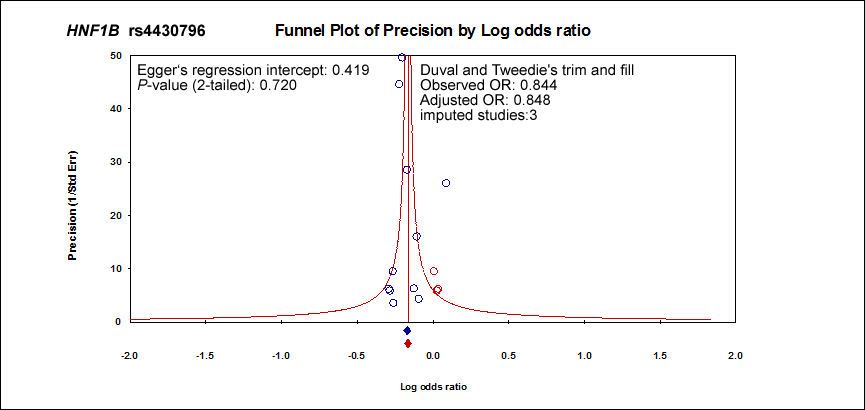


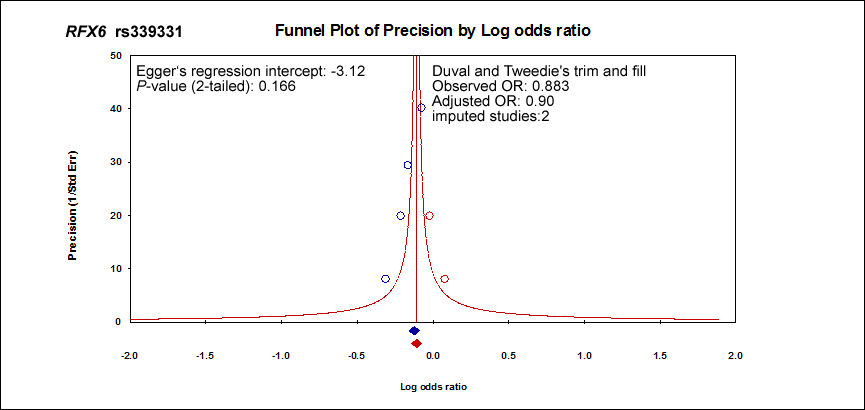


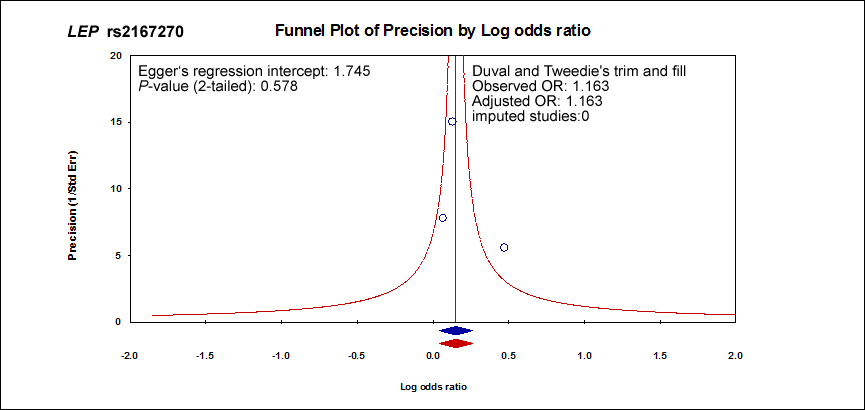


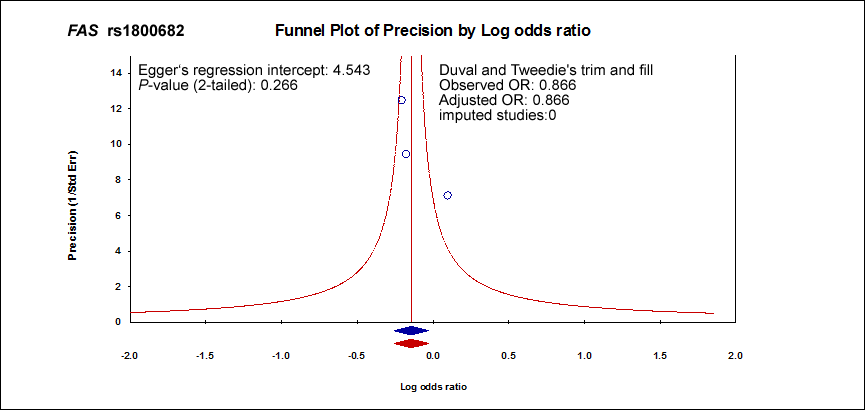


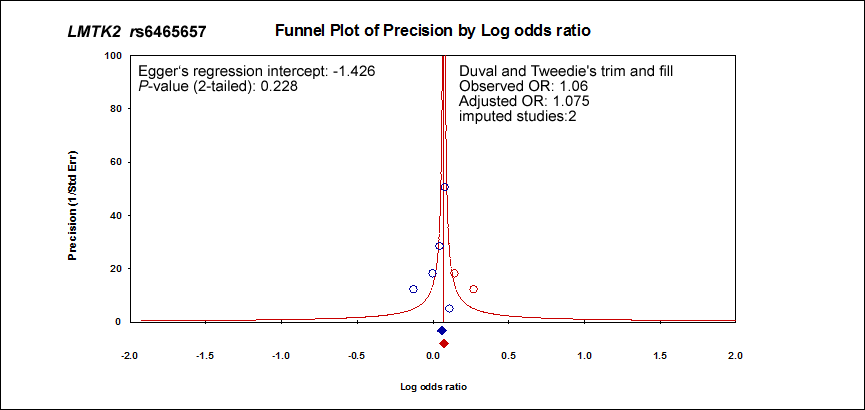


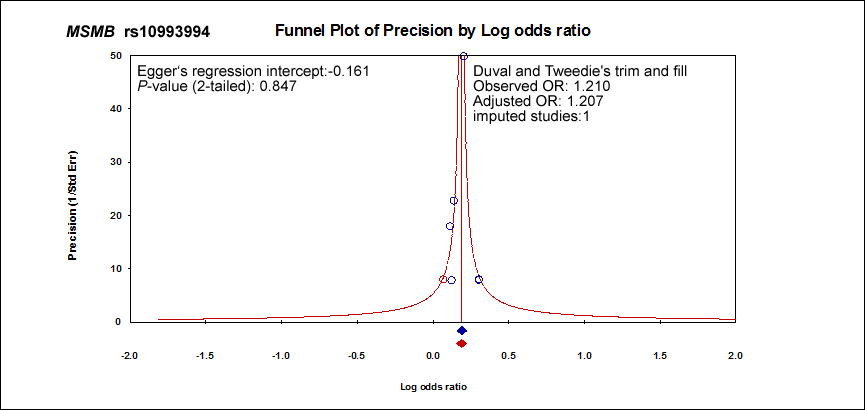

Supplement: Supplementary file 7 [file oncotarget-07-22271-s007.docx]
