# Supplementary material for: Systematic meta-analyses of gene-specific genetic association studies in prostate cancer
Source: Oncotarget. 2016 Mar 5;7(16):22271–84. doi: 10.18632/oncotarget.7926 (PMC5008361; doi:10.18632/oncotarget.7926)

**Supplementary Figure 8** The summary of stability of the twenty positive meta-analyses in all ethnic groups using one study removed procedure. Author's name followed by (a) or (b) or (c) et al. represented the same author performed different studies. The summary OR and 95% c.i. were re-computed when a study is removed from the meta analysis.


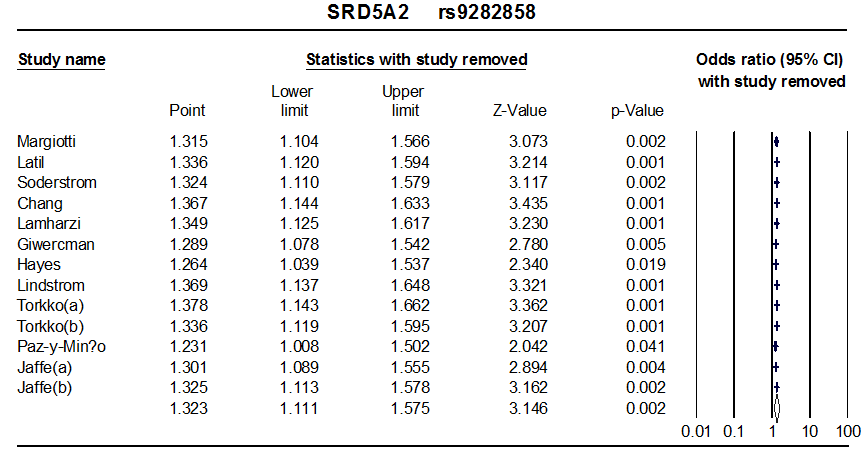


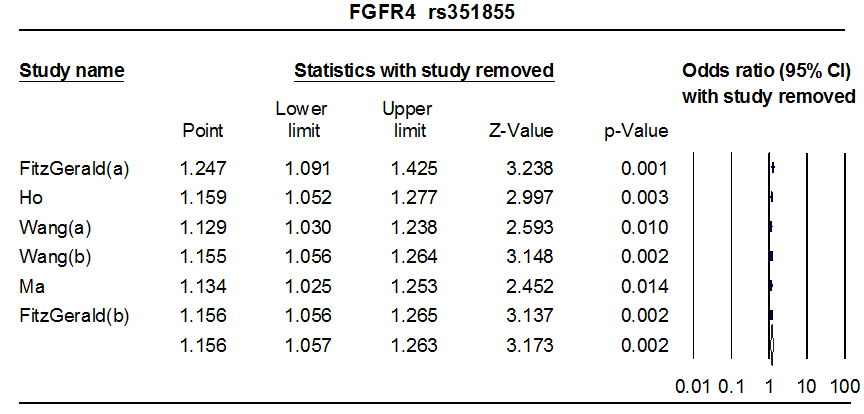


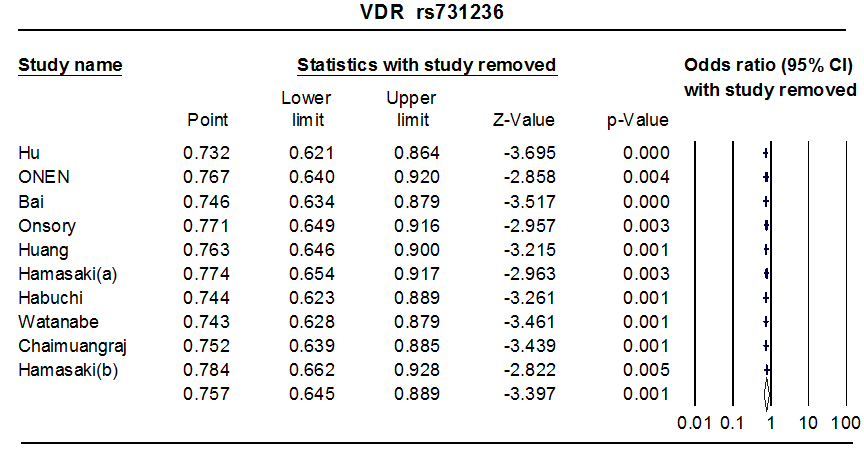


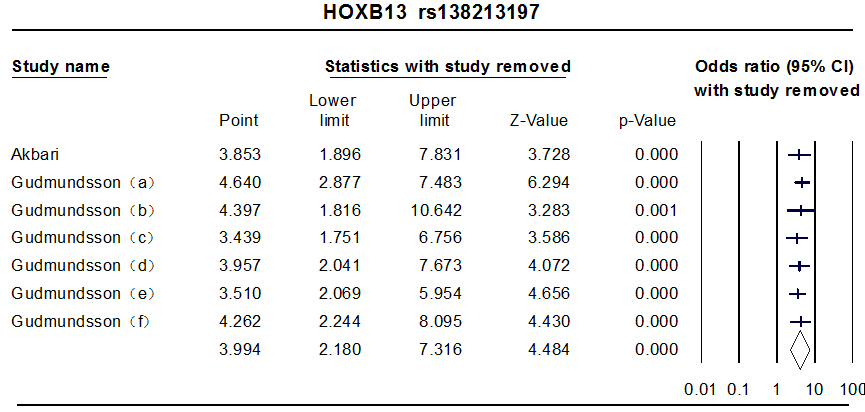


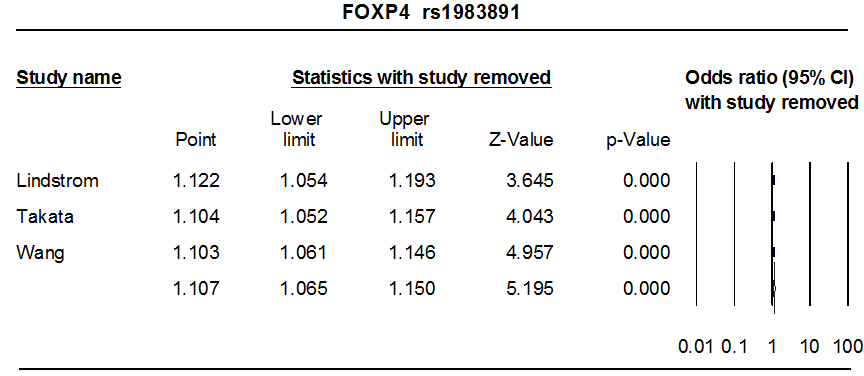


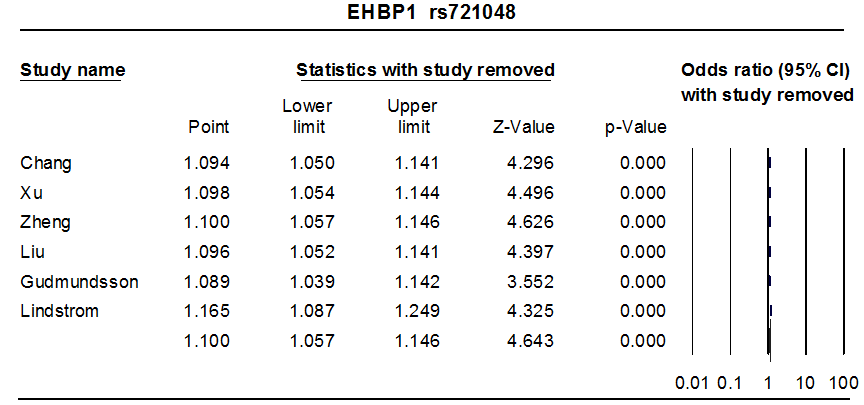


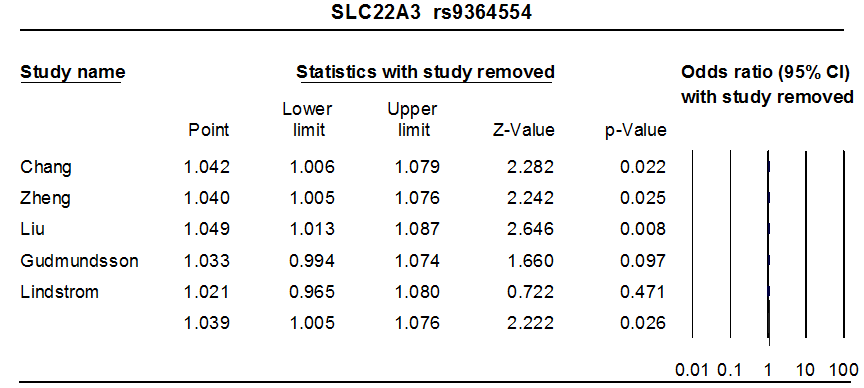


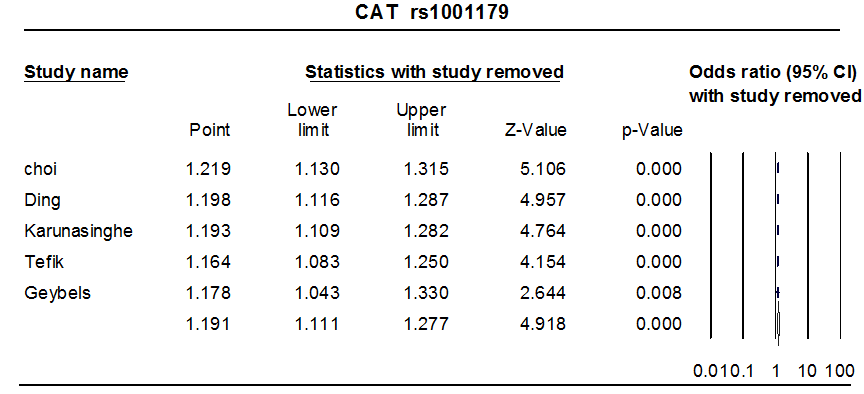


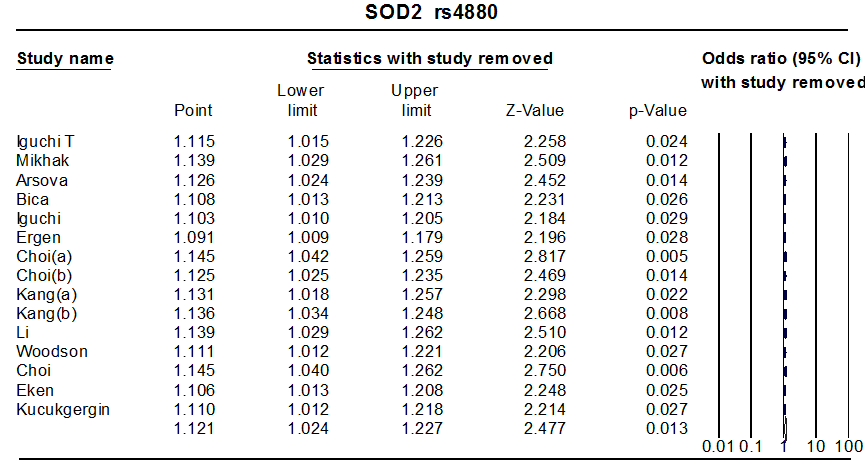


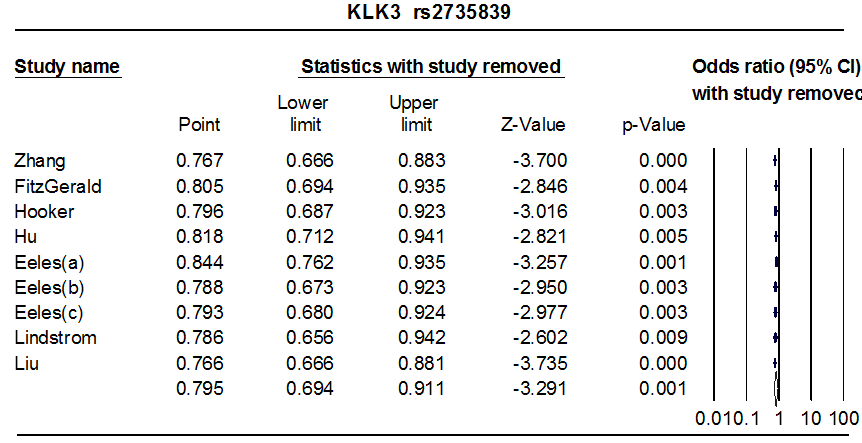


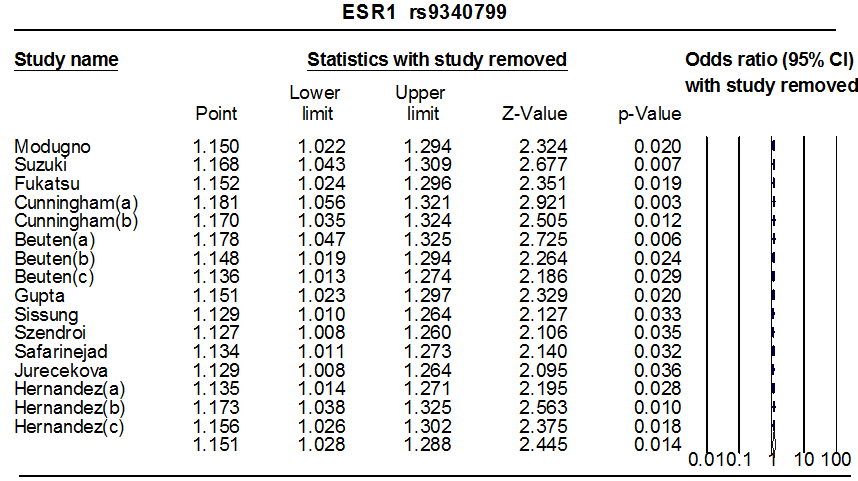


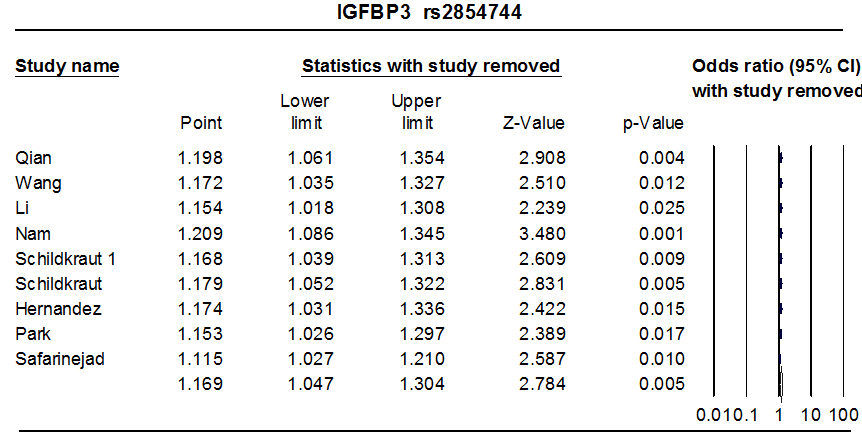


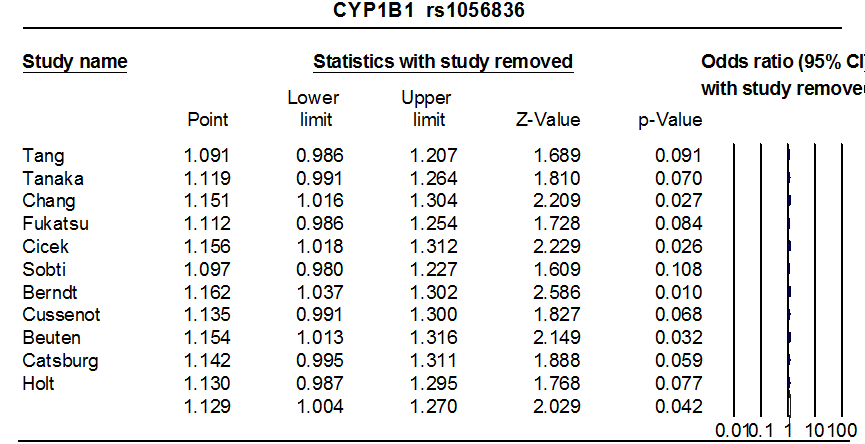


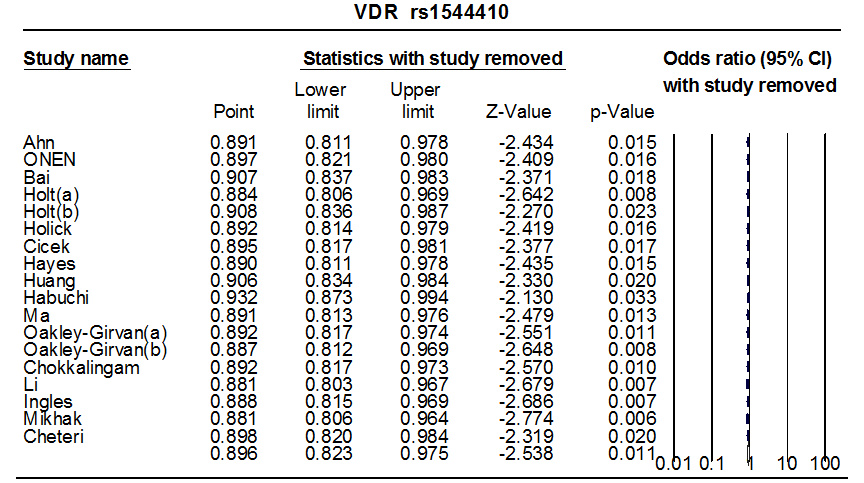


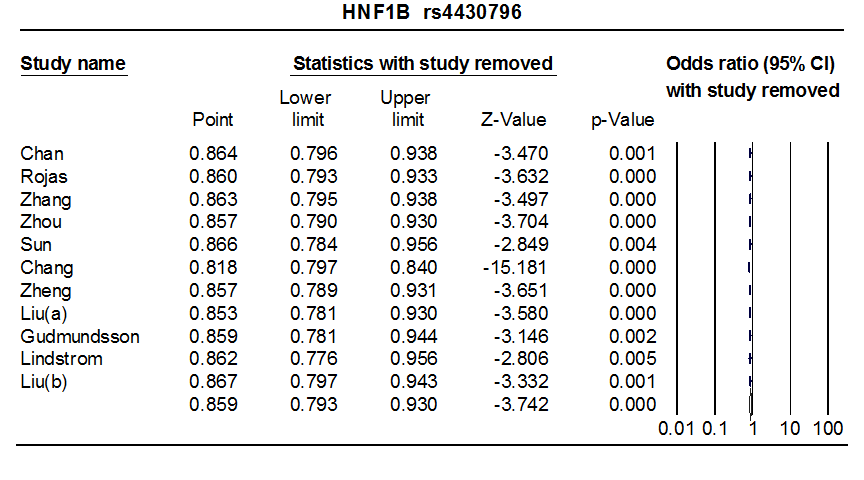


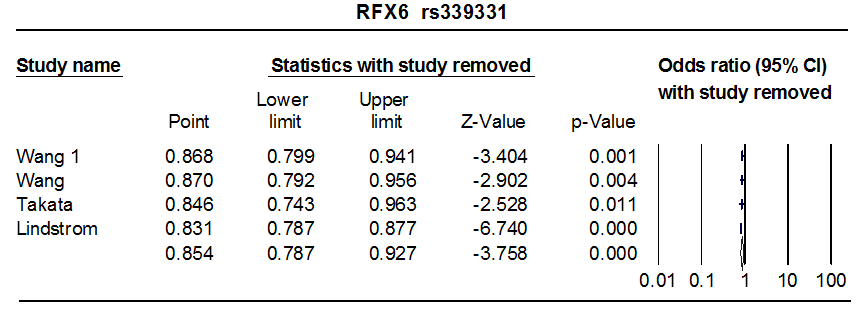


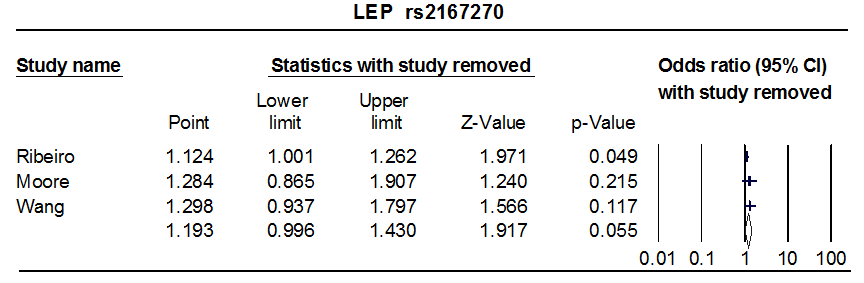


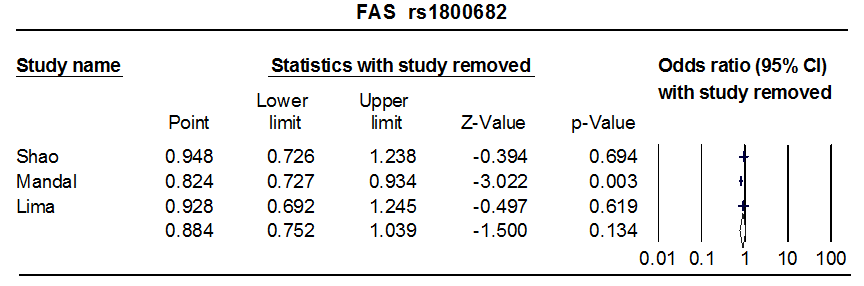


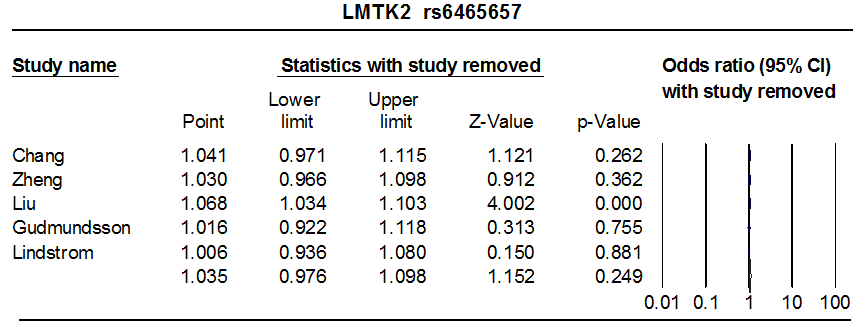


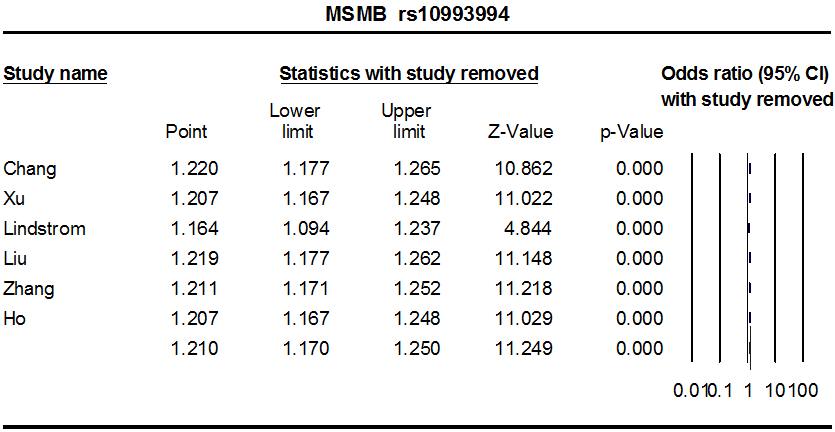

Supplement: Supplementary file 8 [file oncotarget-07-22271-s008.docx]
