# Supplementary material for: Systematic meta-analyses of gene-specific genetic association studies in prostate cancer
Source: Oncotarget. 2016 Mar 5;7(16):22271–84. doi: 10.18632/oncotarget.7926 (PMC5008361; doi:10.18632/oncotarget.7926)

**Supplementary Figure 9** the summary of cumulative analyses for the positive meta-analyses in all ethnic groups. In cumulative analysis, the studies were chronologically sorted, and then the summary OR and 95% c.i. was calculated as a new study was added.


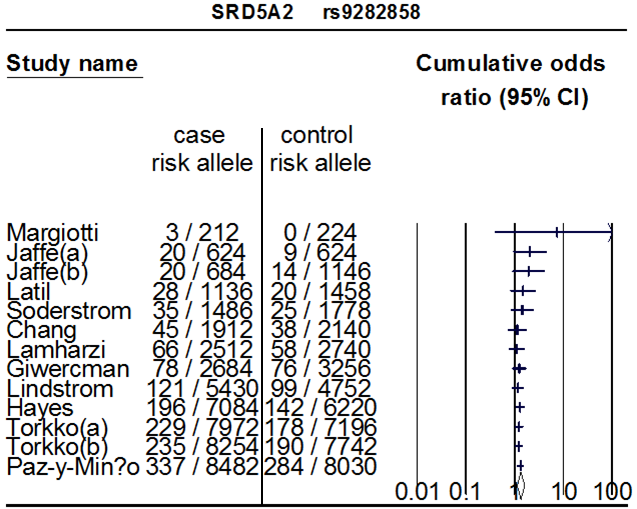


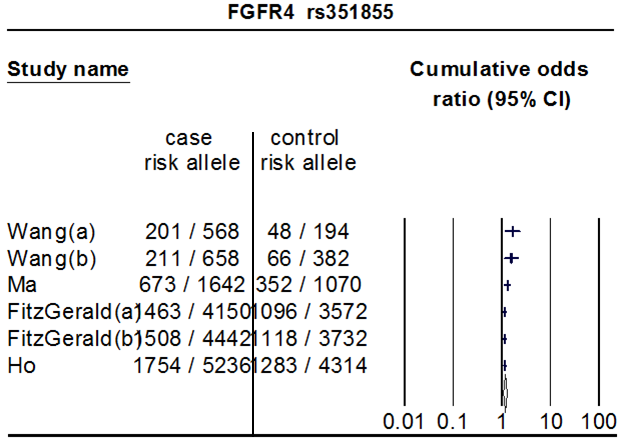


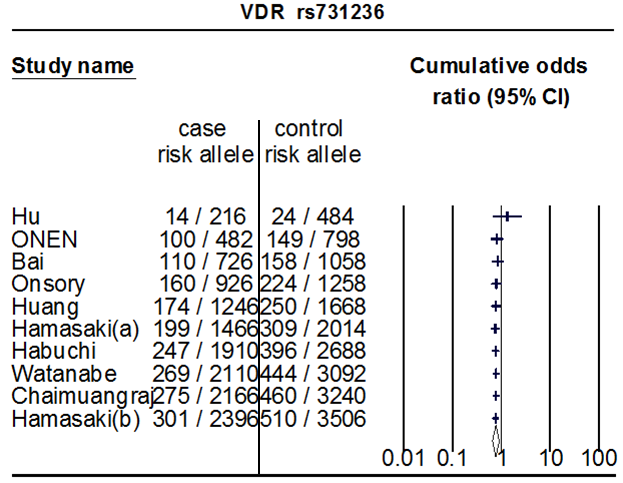


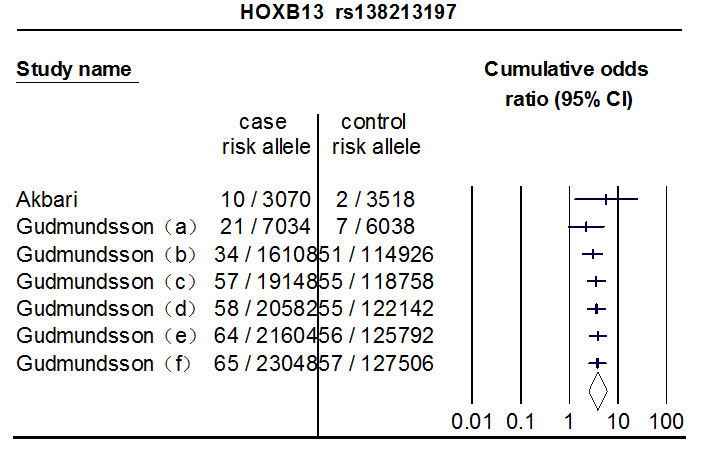


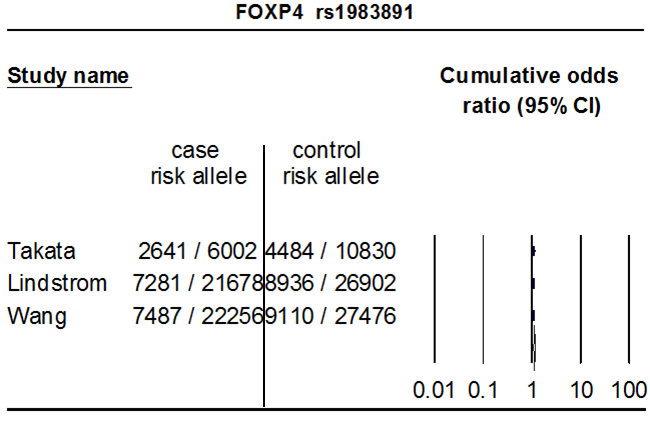


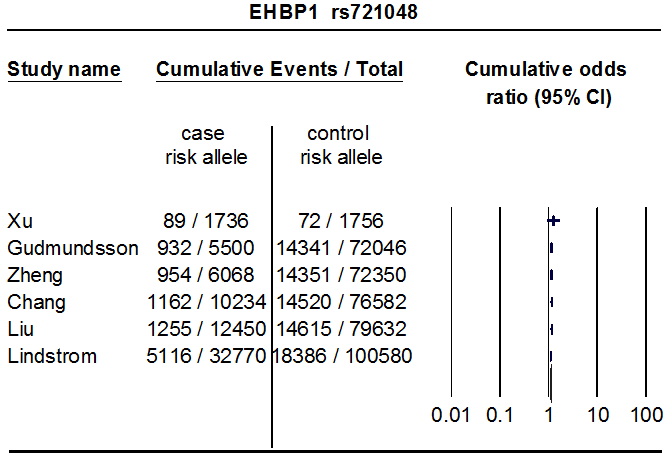


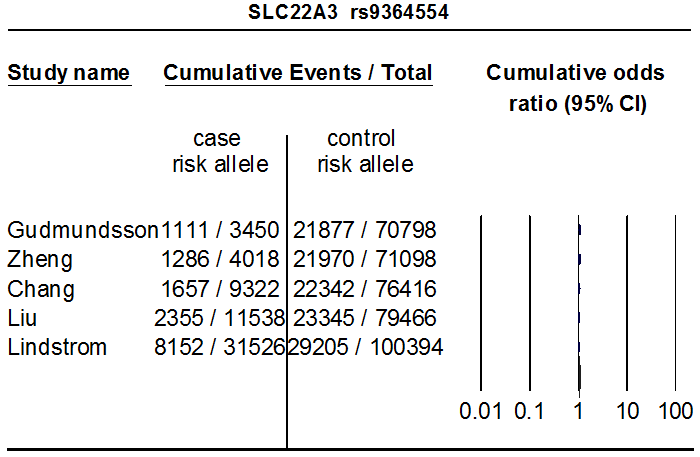


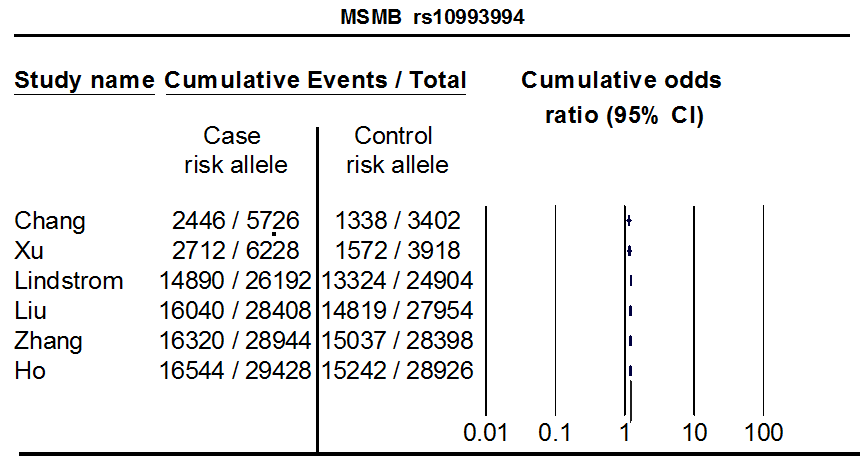


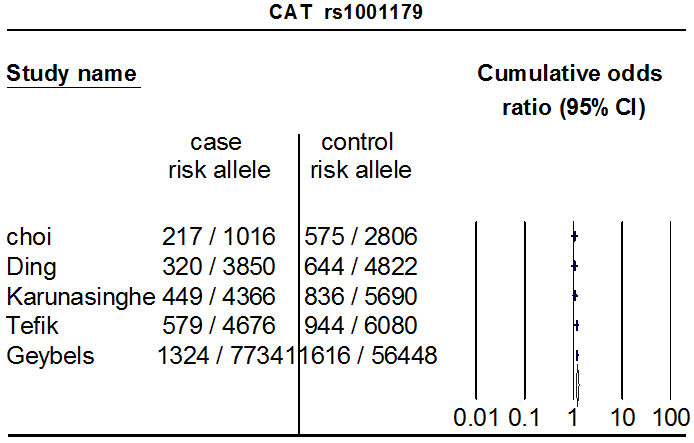


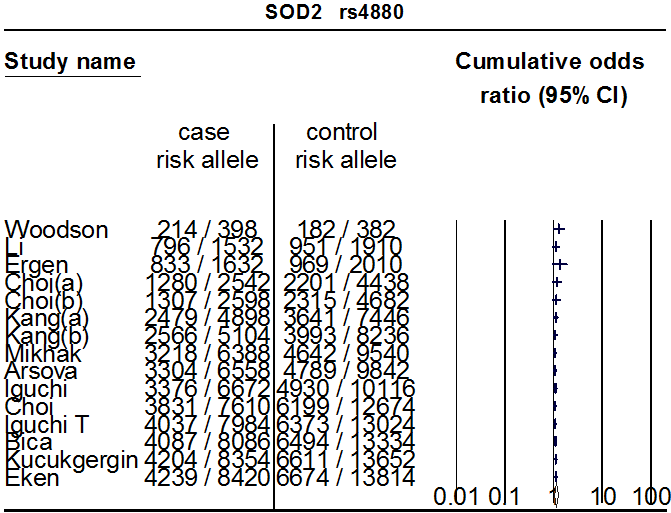


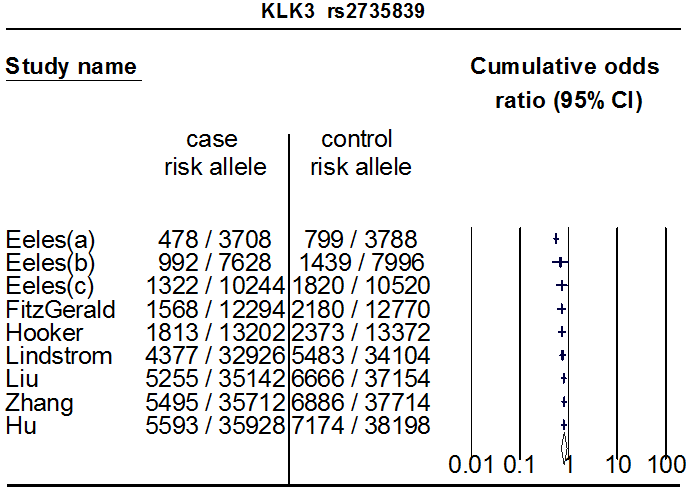


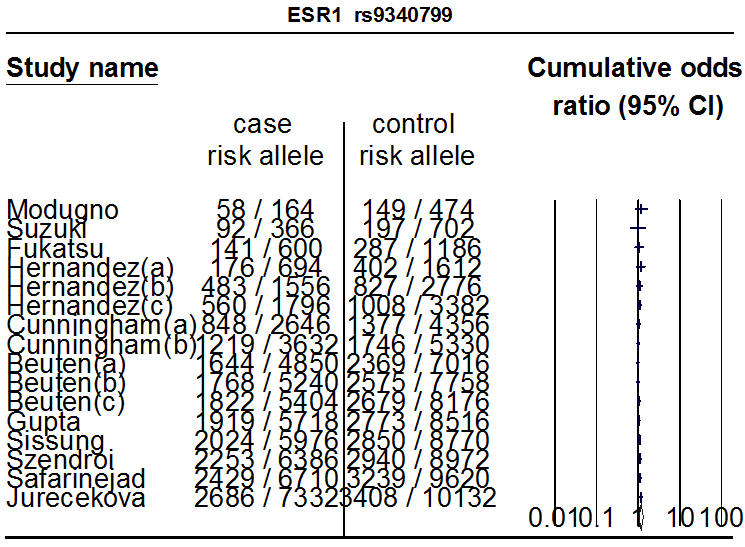


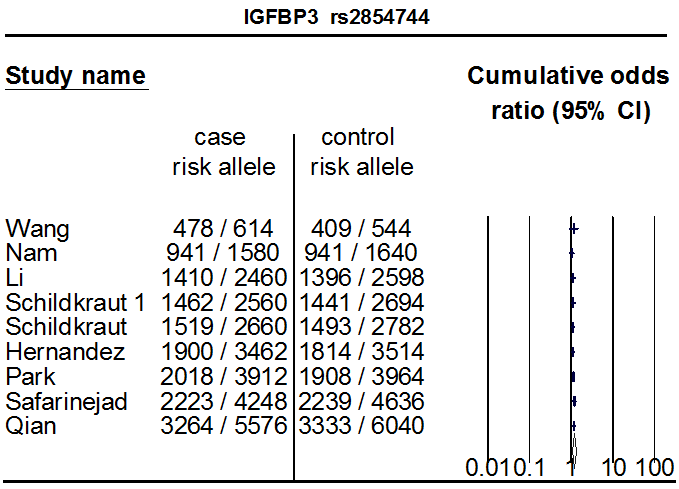


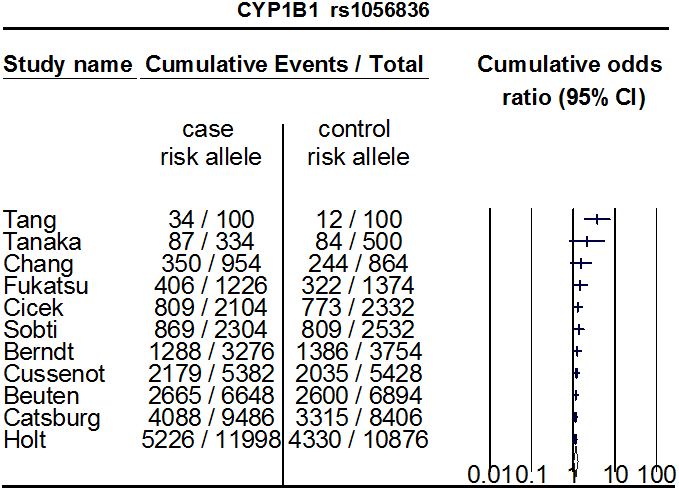


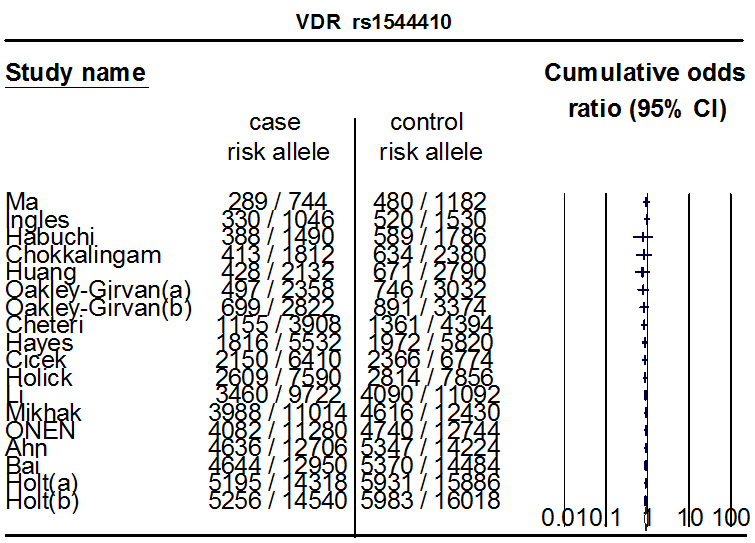


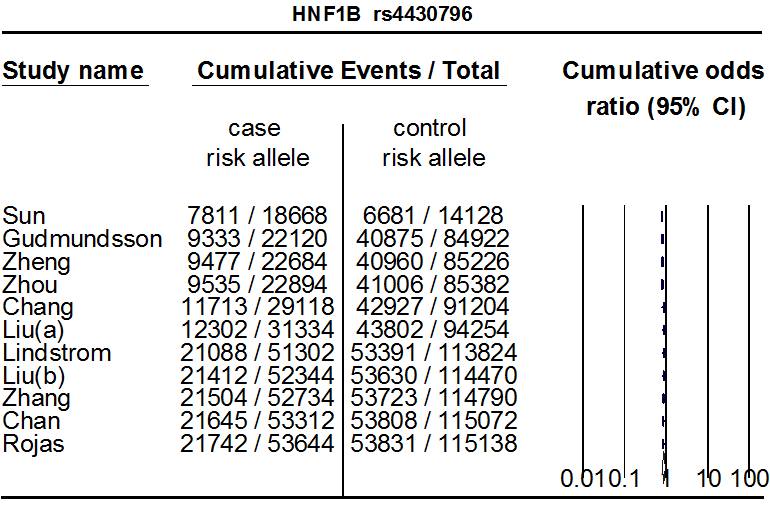


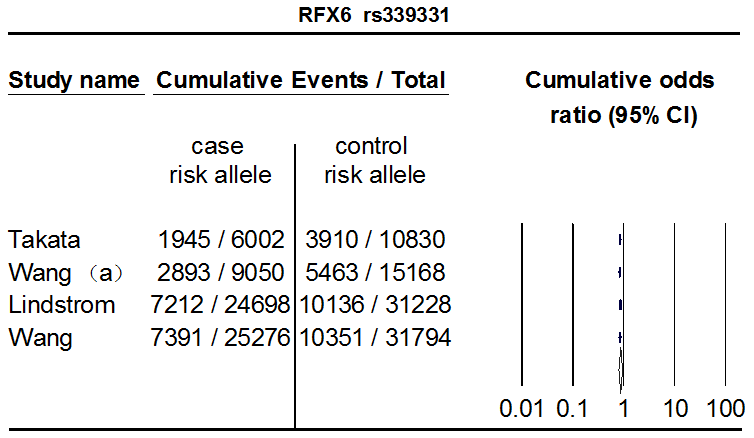


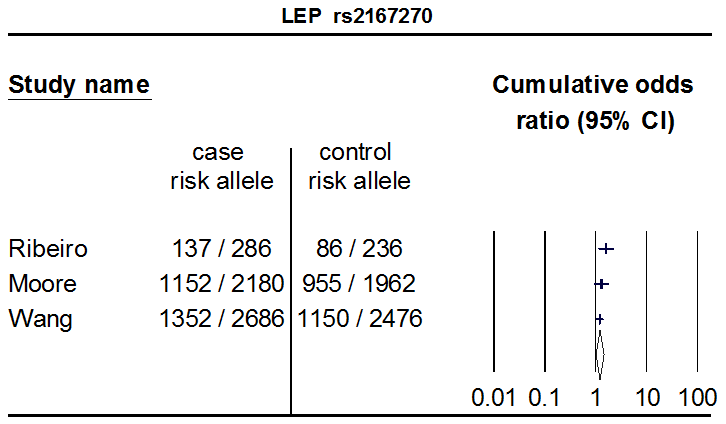


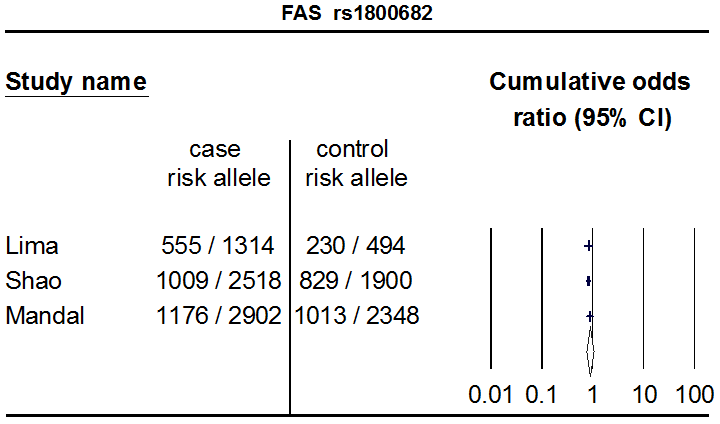


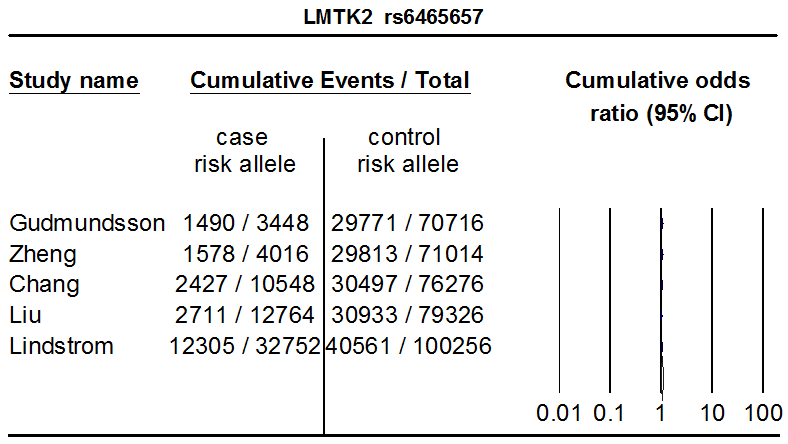

Supplement: Supplementary file 9 [file oncotarget-07-22271-s009.docx]
