# Supplementary material for: Systematic meta-analyses of gene-specific genetic association studies in prostate cancer
Source: Oncotarget. 2016 Mar 5;7(16):22271–84. doi: 10.18632/oncotarget.7926 (PMC5008361; doi:10.18632/oncotarget.7926)
Supplement: Supplementary file 11 [file oncotarget-07-22271-s011.docx]

**Supplementary Table 2 Random-effects meta-analyses using allelic contrasts for SNVs showing non-significant summary ORs (as of August 1, 2015 )**

| Gene | SNVs | Model | OR (95% c.i.) ^a^,  *P*-value | Q-value ^b^ | Heterogeneity ^c^, *P*-value | Cases versus controls (Number of independent samples) |
| --- | --- | --- | --- | --- | --- | --- |
| *MGMT* | rs12917 | T vs. C, all ethnicities | 1.249 (0.805–1.937)  *P* = 0.321 | 7.600 | 0.022 | 1558 vs. 1564 (3) |
|  |  | T vs. C, all excl. initial study | 0.970 (0.831–1.133)  *P* = 0.703 | 0.686 | 0.408 | 1397 vs. 1318 (2) |
| *XRCC1* | rs25487 | A vs. G, all ethnicities | 1.083 (0.989–1.186)  *P* = 0.083 | 28.252 | 0.029 | 4910 vs.4735 (17) |
|  |  | A vs. G, all excl. initial study | 1.096 (1.000–1.201)  *P* = 0.051 | 26.569 | 0.032 | 4834 vs. 4553 (16) |
|  |  | A vs. G, all excl. HWE study | 1.094 (0.991–1.206)  *P* = 0.074 | 26.073 | 0.025 | 4524 vs. 4289 (15) |
| *OGG1* | rs1052133 | G vs. C, all ethnicities | 1.123 (0.943–1.337)  *P* = 0.192 | 41.835 | 0.000 | 3071 vs.3808 (11) |
|  |  | G vs. C, all excl. initial study | 1.170 (0.978–1.401)  *P* = 0.086 | 36.804 | 0.000 | 2773 vs. 3634 (10) |
|  |  | G vs. C, all excl. HWE study | 1.193 (0.970–1.466)  *P* = 0.095 | 28.893 | 0.000 | 1636 vs. 2238 (9) |
| *XRCC1* | rs1799782 | T vs. C, all ethnicities | 1.017 (0.847–1.222)  *P* = 0.856 | 17.977 | 0.021 | 3230 vs.3060 (9) |
|  |  | T vs. C, all excl. initial study | 1.039 (0.862–1.252)  *P* = 0.686 | 16.473 | 0.021 | 3154 vs. 2878 (8) |
|  |  | T vs. C, all excl. HWE study | 1.040 (0.864–1.253)  *P* = 0.676 | 16.330 | 0.022 | 3084 vs. 2977 (8) |
| *ERCC2* | rs1799793 | G vs. A, all ethnicities | 1.186 (0.985–1.427)  *P* = 0.072 | 22.100 | 0.002 | 6450 vs.3225 (8) |
|  |  | G vs. A, all excl. initial study | 1.233 (0.954–1.593)  *P* = 0.110 | 21.763 | 0.001 | 1984 vs. 2745 (6) |
|  |  | G vs. A, all excl. HWE study | 1.140 (0.902–1.440)  *P* = 0.272 | 13.545 | 0.019 | 1926 vs. 2309 (6) |
| *XPC* | rs2228001 | C vs. A, all ethnicities | 1.051 (0.962–1.148)  *P* = 0.269 | 6.847 | 0.232 | 2195 vs.2208 (6) |
|  |  | C vs. A, all excl. initial study | 1.076 (0.982–1.179)  *P* = 0.117 | 3.375 | 0.497 | 2030 vs. 2043 (5) |
|  |  | C vs. A, all excl. HWE study | 1.045 (0.953–1.146)  *P* = 0.346 | 6.679 | 0.354 | 1966 vs. 1970 (5) |
| *MGMT* | rs2308321 | G vs. A, all ethnicities | 1.050 (0.911–1.211)  *P* = 0.502 | 2.335 | 0.506 | 1820 vs.3009 (4) |
|  |  | G vs. A, all excl. initial study | 1.038 (0.899–1.198)  *P* = 0.609 | 0.773 | 0.679 | 1659 vs. 2761 (3) |
| *XRCC1* | rs25489 | A vs. G, all ethnicities | 1.086 (0.966–1.221)  *P* = 0.168 | 2.505 | 0.868 | 2612 vs.2775 (7) |
|  |  | A vs. G, all excl. initial study | 1.081 (0.960–1.217)  *P* = 0.200 | 2.205 | 0.820 | 2536 vs. 2593 (6) |
|  |  | A vs. G, all excl. HWE study | 1.147 (0.929–1.417)  *P* = 0.203 | 1.011 | 0.799 | 1673 vs. 1753 (4) |
| *KLK3* | rs266882 | A vs. G, all ethnicities | 1.053 (0.900–1.232)  *P* = 0.517 | 28.210 | 0.000 | 2839 vs.2761 (9) |
|  |  | A vs. G, all excl. initial study | 1.081 (0.918–1.272)  *P* = 0.349 | 26.238 | 0.000 | 2782 vs. 2605 (8) |
| *SRD5A2* | rs523349 | G vs. C, all ethnicities | 1.076 (0.997–1.161)  *P* = 0.059 | 66.190 | 0.000 | 9555 vs.9566 (30) |
|  |  | G vs. C, all excl. initial study | 1.076 (0.995–1.164)  *P* = 0.065 | 65.220 | 0.000 | 9447 vs. 9410 (28) |
|  |  | G vs. C, all excl. HWE study | 1.039 (0.975–1.106)  *P* = 0.235 | 43.402 | 0.032 | 9441 vs. 9422 (29) |
| *CYP17A1* | rs743572 | C vs. T, all ethnicities | 0.989 (0.930–1.051)  *P* = 0.715 | 60.314 | 0.002 | 16103 vs.16635 (33) |
|  |  | C vs. T, all excl. initial study | 0.988 (0.929–1.050)  *P* = 0.696 | 59.839 | 0.001 | 16091 vs. 16627 (31) |
|  |  | C vs. T, all excl. HWE study | 0.970 (0.912–1.031)  *P* = 0.330 | 49.474 | 0.007 | 15626 vs. 15822 (29) |
| *ESR1* | rs2234693 | C vs. T, all ethnicities | 1.091 (0.998–1.193)  *P* = 0.056 | 51.233 | 0.000 | 4725 vs.10453 (22) |
|  |  | C vs. T, all excl. initial study | 1.085 (0.990–1.189)  *P* = 0.080 | 50.194 | 0.000 | 4644 vs. 10216 (21) |
|  |  | C vs. T, all excl. HWE study | 1.095 (0.998–1.202)  *P* = 0.056 | 51.208 | 0.000 | 4545 vs. 10276 (21) |
| *CYP3A4* | rs2740574 | G vs. A, all ethnicities | 1.165 (0.955–1.422)  *P* = 0.132 | 26.068 | 0.004 | 1653 vs.1760 (11) |
|  |  | G vs. A, all excl. initial study | 1.129 (0.912–1.397)  *P* = 0.264 | 23.434 | 0.005 | 1479 vs. 1644 (10) |
|  |  | G vs. A, all excl. HWE study | 1.131 (0.917–1.396)  *P* = 0.251 | 24.060 | 0.004 | 1569 vs. 1624 (10) |
| *CXCL8* | rs4073 | T vs. A, all ethnicities | 0.993 (0.908–1.086)  *P* = 0.877 | 5.635 | 0.228 | 1942 vs.1964 (5) |
|  |  | T vs. A, all excl. initial study | 1.021 (0.928–1.123)  *P* = 0.674 | 3.018 | 0.389 | 1704 vs. 1729 (4) |
| *IL18* | rs187238 | G vs. C, all ethnicities | 0.883 (0.452–1.726)  *P* = 0.716 | 33.801 | 0.000 | 931 vs.971 (3) |
|  |  | G vs. C, all excl. initial study | 0.639 (0.443–0.924)  *P* = 0.017 | 3.424 | 0.064 | 666 vs. 691 (2) |
| *IL6* | rs1800795 | C vs. G, all ethnicities | 1.028 (0.914–1.156)  *P* = 0.650 | 31.022 | 0.000 | 10941 vs.13235 (10) |
|  |  | C vs. G, all excl. initial study | 1.009 (0.885–1.151)  *P* = 0.890 | 28.399 | 0.000 | 10457 vs. 12622 (9) |
|  |  | C vs. G, all excl. HWE study | 1.062 (0.982–1.149)  *P* = 0.130 | 5.395 | 0.494 | 2745 vs. 2891 (7) |
| *IL10* | rs1800871 | T vs. C, all ethnicities | 0.951 (0.849–1.066)  *P* = 0.389 | 13.656 | 0.058 | 3182 vs.4095 (8) |
|  |  | T vs. C, all excl. initial study | 0.962 (0.835–1.108)  *P* = 0.592 | 12.557 | 0.051 | 1936 vs. 2333 (7) |
|  |  | T vs. C, all excl. HWE study | 0.952 (0.835–1.085)  *P* = 0.461 | 13.655 | 0.034 | 2991 vs. 3460 (7) |
| *IL10* | rs1800872 | A vs. C, all ethnicities | 0.993 (0.908–1.085)  *P* = 0.872 | 9.597 | 0.143 | 2346 vs.2672 (7) |
|  |  | A vs. C, all excl. initial study | 0.975 (0.847–1.124)  *P* = 0.730 | 9.426 | 0.093 | 1799 vs. 2127 (6) |
| *IL10* | rs1800896 | G vs. A, all ethnicities | 0.973 (0.867–1.093)  *P* = 0.646 | 14.172 | 0.048 | 3155 vs.4080 (8) |
|  |  | G vs. A, all excl. initial study | 1.021 (0.949–1.097)  *P* = 0.583 | 9.387 | 0.153 | 2908 vs. 3857 (7) |
|  |  | G vs. A, all excl. HWE study | 0.972 (0.854–1.105)  *P* = 0.662 | 9.784 | 0.044 | 2668 vs. 3422 (5) |
| *IL18* | rs1946518 | T vs. A, all ethnicities | 0.866 (0.646–1.161)  *P* = 0.337 | 10.356 | 0.006 | 931 vs.971 (3) |
|  |  | T vs. A, all excl. initial study | 0.835 (0.513–1.097)  *P* = 0.583 | 10.100 | 0.001 | 666 vs. 691 (2) |
| *MPO* | rs2333227 | A vs. G, all ethnicities | 0.790 (0.606–1.029)  *P* = 0.080 | 4.819 | 0.090 | 762 vs.1678 (3) |
|  |  | A vs. G, all excl. initial study | 0.687 (0.517–0.914)  *P* = 0.010 | 1.222 | 0.269 | 269 vs. 346 (2) |
| *COMT* | rs4680 | T vs. C, all ethnicities | 0.969 (0.890–1.056)  *P* = 0.476 | 8.367 | 0.212 | 2292 vs.2158 (7) |
|  |  | T vs. C, all excl. initial study | 0.961 (0.881–1.050)  *P* = 0.379 | 7.630 | 0.178 | 2191 vs. 2044 (6) |
|  |  | T vs. C, all excl. HWE study | 0.968 (0.888–1.056)  *P* = 0.467 | 8.345 | 0.138 | 2251 vs. 2051 (6) |
| *ADIPOQ* | rs266729 | G vs. C, all ethnicities | 0.975 (0.814–1.166)  *P* = 0.780 | 10.548 | 0.014 | 2697 vs.2974 (4) |
|  |  | G vs. C, all excl. initial study | 0.966 (0.786–1.187)  *P* = 0.740 | 10.540 | 0.005 | 2566 vs. 2660 (3) |
|  |  | G vs. C, all excl. HWE study | 1.070 (0.975–1.174)  *P* = 0.155 | 1.657 | 0.437 | 2240 vs. 2533 (3) |
| *RNASEL* | rs486907 | A vs. G, all ethnicities | 1.078 (0.954–1.219)  *P* = 0.227 | 20.579 | 0.008 | 4075 vs.4277 (9) |
|  |  | A vs. G, all excl. initial study | 1.078 (0.942–1.234)  *P* = 0.273 | 20.181 | 0.005 | 3842 vs. 4101 (8) |
|  |  | A vs. G, all excl. HWE study | 1.049 (0.936–1.174)  *P* = 0.412 | 13.100 | 0.041 | 3824 vs. 3933 (7) |
| *RNASEL* | rs627928 | G vs. T, all ethnicities | 1.066 (0.947–1.199) *P* = 0.289 | 14.264 | 0.047 | 3089 vs.3154 (8) |
|  |  | G vs. T, all excl. initial study | 1.058 (0.928–1.208) *P* = 0.393 | 14.013 | 0.029 | 2856 vs. 2978 (7) |
|  |  | G vs. T, all excl. HWE study | 1.081 (0.928–1.258) *P* = 0.316 | 13.489 | 0.036 | 1874 vs. 1960 (7) |
| *TP53* | rs1042522 | G vs. C, all ethnicities | 0.811 (0.605–1.087) *P* = 0.161 | 58.807 | 0.000 | 1274 vs.1543 (10) |
|  |  | G vs. C, all excl. initial study | 0.806 (0.584–1.112) *P* = 0.189 | 58.807 | 0.000 | 1165 vs. 1397 (9) |
|  |  | G vs. C, all excl. HWE study | 0.832 (0.599–1.155) *P* = 0.272 | 24.266 | 0.000 | 718 vs. 1000 (6) |
| *GPX1* | rs1050450 | T vs. C, all ethnicities | 1.165 (0.875–1.552) *P* = 0.296 | 30.429 | 0.000 | 1258 vs.2686 (6) |
|  |  | T vs. C, all excl. initial study | 1.204 (0.815–1.778) *P* = 0.352 | 27.690 | 0.000 | 758 vs. 1295 (5) |
| *CASC8* | rs1447295 | A vs. C, all ethnicities | 1.204 (0.981–1.478) *P* = 0.075 | 100.558 | 0.000 | 6942 vs.5622 (14) |
|  |  | A vs. C, all excl. initial study | 1.166 (0.942–1.445) *P* = 0.159 | 91.583 | 0.000 | 5396 vs. 5051 (13) |
|  |  | A vs. C, all excl. HWE study | 1.324 (1.135–1.543) *P* = 0.000 | 49.426 | 0.000 | 6792 vs. 5522 (13) |
| *VEGFA* | rs1570360 | A vs. G, all ethnicities | 0.787 (0.554–1.120) *P* = 0.138 | 11.312 | 0.003 | 1497 vs.1535 (3) |
|  |  | A vs. G, all excl. initial study | 0.739 (0.367–1.488) *P* = 0.138 | 9.899 | 0.002 | 1259 vs. 1272 (2) |
| *NQO1* | rs1800566 | T vs. C, all ethnicities | 1.171 (0.921–1.488)  *P* = 0.198 | 10.643 | 0.059 | 717 vs.1794 (6) |
|  |  | T vs. C, all excl. initial study | 1.193 (0.909–1.565)  *P* = 0.204 | 10.430 | 0.034 | 663 vs. 1694 (5) |
|  |  | T vs. C, all excl. HWE study | 1.179 (0.897–1.550)  *P* = 0.237 | 10.618 | 0.031 | 672 vs. 1744 (5) |
| *MTHFR* | rs1801131 | C vs. A, all ethnicities | 0.989 (0.832–1.175)  *P* = 0.896 | 2.845 | 0.416 | 713 vs.1114 (4) |
|  |  | C vs. A, all excl. initial study | 1.031 (0.845–1.258)  *P* = 0.762 | 2.132 | 0.344 | 496 vs. 894 (3) |
|  |  | C vs. A, all excl. HWE study | 0.977 (0.821–1.162)  *P* = 0.791 | 1.644 | 0.440 | 609 vs. 1004 (3) |
| *MTHFR* | rs1801133 | T vs. C, all ethnicities | 0.855 (0.676–1.082)  *P* = 0.193 | 23.698 | 0.000 | 3485 vs.2692 (6) |
|  |  | T vs. C, all excl. initial study | 0.881 (0.685–1.133)  *P* = 0.324 | 22.034 | 0.000 | 3390 vs. 2655 (5) |
|  |  | T vs. C, all excl. HWE study | 0.798 (0.618–1.032)  *P* = 0.085 | 16.572 | 0.001 | 3286 vs. 2545 (4) |
| *MTR* | rs1805087 | G vs. A, all ethnicities | 1.056 (0.845–1.321)  *P* = 0.630 | 3.255 | 0.196 | 691 vs.615 (3) |
|  |  | G vs. A, all excl. initial study | 1.359 (0.593–3.118)  *P* = 0.468 | 3.246 | 0.072 | 474 vs. 395 (2) |
|  |  | G vs. A, all excl. HWE study | 1.004 (0.797–1.264)  *P* = 0.972 | 0.109 | 0.741 | 587 vs. 505 (2) |
| *MDM2* | rs2279744 | G vs. T, all ethnicities | 0.841 (0.680–1.040)  *P* = 0.109 | 6.411 | 0.093 | 732 vs. 836 (4) |
|  |  | G vs. T, all excl. initial study | 0.857 (0.650–1.129)  *P* = 0.273 | 6.092 | 0.048 | 587 vs. 712 (3) |
| *CYP1A1* | rs4646903 | C vs. T, all ethnicities | 1.082 (0.922–1.269)  *P* = 0.336 | 19.640 | 0.020 | 2604 vs.2675 (10) |
|  |  | C vs. T, all excl. initial study | 1.063 (0.891–1.267)  *P* = 0.497 | 19.323 | 0.013 | 2489 vs. 2475 (9) |
|  |  | C vs. T, all excl. HWE study | 1.082 (0.901–1.301)  *P* = 0.398 | 19.367 | 0.013 | 2396 vs. 2445 (9) |
| *CASC8* | rs6983267 | T vs. G, all ethnicities | 0.988 (0.866–1.128)  *P* = 0.857 | 42.206 | 0.000 | 5837 vs.4612 (12) |
|  |  | T vs. G, all excl. initial study | 1.016 (0.883–1.168)  *P* = 0.826 | 37.708 | 0.000 | 4286 vs.4039 (11) |
|  |  | T vs. G, all excl. HWE study | 0.993 (0.867–1.137)  *P* = 0.919 | 46.061 | 0.000 | 5748 vs. 4523 (11) |
| *CDH1* | rs16260 | A vs. C, all ethnicities | 1.085(0.867–1.358)  *P* = 0.477 | 20.128 | 0.001 | 2378 vs.1973 (6) |
|  |  | A vs. C, all excl. initial study | 1.050(0.813–1.356)  *P* = 0.710 | 18.562 | 0.001 | 2159 vs.1754 (5) |
|  |  | A vs. C, all excl. HWE study | 1.005(0.751–1.344)  *P* = 0.974 | 11.068 | 0.011 | 1432 vs. 1184 (4) |
| *PTGS2* | rs20417 | C vs. G, all ethnicities | 1.005(0.893–1.132)  *P* = 0.929 | 12.938 | 0.074 | 10600 vs.13021 (8) |
|  |  | C vs. G, all excl. initial study | 1.005(0.883–1.143)  *P* = 0.941 | 11.696 | 0.039 | 10240 vs.12660 (6) |
|  |  | C vs. G, all excl. HWE study | 0.945(0.739–1.208)  *P* = 0.650 | 11.606 | 0.021 | 2265 vs. 4094 (5) |
| *XRCC3* | rs861539 | T vs. C, all ethnicities | 0.845(0.675–1.057)  *P* = 0.141 | 0.913 | 0.634 | 499 vs.571 (3) |
|  |  | T vs. C, all excl. initial study | 0.813(0.636–1.040)  *P* = 0.099 | 0.350 | 0.554 | 340 vs.324 (2) |
|  |  | T vs. C, all excl. HWE study | 0.823(0.625–1.085)  *P* = 0.650 | 0.813 | 0.367 | 383 vs. 439 (2) |
| *THADA* | rs1465618 | A vs. G, all ethnicities | 0.921(0.726–1.168)  *P* = 0.498 | 22.730 | 0.000 | 11151 vs.11429 (3) |
|  |  | A vs. G, all excl. initial study | 0.938 (0.636–1.385)  *P* = 0.749 | 9.588 | 0.002 | 10043 vs.9904 (2) |
| *NOS3* | rs1799983 | T vs. G, all ethnicities | 1.010 (0.914–1.116)  *P* = 0.843 | 3.092 | 0.797 | 1792 vs.2411 (7) |
|  |  | T vs. G, all excl. initial study | 0.998 (0.899–1.107)  *P* = 0.966 | 2.429 | 0.787 | 1667 vs.2258 (6) |
| *NKX3-1* | rs2228013 | T vs. C, all ethnicities | 1.057 (0.907–1.232)  *P* = 0.477 | 3.017 | 0.221 | 4597 vs.3626 (3) |
|  |  | T vs. C, all excl. initial study | 1.018 (0.860–1.205)  *P* = 0.835 | 1.934 | 0.164 | 4039 vs.2931 (2) |
| *PTGS2* | rs2745557 | T vs. C, all ethnicities | 0.923 (0.804–1.060)  *P* = 0.256 | 8.813 | 0.066 | 10020 vs.10234 (5) |
|  |  | T vs. C, all excl. initial study | 0.856 (0.707–1.038)  *P* = 0.113 | 7.041 | 0.071 | 8665 vs.9469 (4) |
| *JAZF1* | rs10486567 | A vs. G, all ethnicities | 0.951 (0.828–1.093)  *P* = 0.477 | 21.972 | 0.000 | 14800 vs.13433 (5) |
|  |  | A vs. G, all excl. initial study | 0.908 (0.803–1.028)  *P* = 0.126 | 14.566 | 0.002 | 14521 vs.13280 (4) |
| *C2orf43* | rs13385191 | G vs. A, all ethnicities | 1.003 (0.849–1.183)  *P* = 0.976 | 24.114 | 0.000 | 11097 vs.13719 (3) |
|  |  | G vs. A, all excl. initial study | 1.074 (1.022–1.129)  *P* = 0.005 | 0.126 | 0.722 | 8096 vs.8304 (2) |
| *PTGS2* | rs5275 | C vs. T, all ethnicities | 1.011(0.974–1.050)  *P* = 0.555 | 5.433 | 0.246 | 12220 vs.12496 (5) |
|  |  | C vs. T, all excl. initial study | 1.016 (0.977–1.056)  *P* = 0.427 | 4.781 | 0.189 | 10873 vs.11739 (4) |
|  |  | C vs. T, all excl. HWE study | 1.044(0.978–1.113)  *P* = 0.196 | 4.075 | 0.253 | 4330 vs. 4031 (4) |

Note: ‘All ethnicities’ represented that all studies, regardless of ethnicity were meta-analyzed; ‘all excl. initial study’ represented that the first publication reporting on statistic association for any locus were excluded; ' all excl. HWE study' represented that the studies deviating from HWE were excluded. a, the summary OR and 95% c.i. values. b, Q statistic across crude ORs was calculated for each included study. c, *P* > 0.1 is usually considered as an evidence for no between-study heterogeneity; *P* < 0.1 as an evidence for between-study heterogeneity.
