# Supplementary material for: Systematic meta-analyses of gene-specific genetic association studies in prostate cancer
Source: Oncotarget. 2016 Mar 5;7(16):22271–84. doi: 10.18632/oncotarget.7926 (PMC5008361; doi:10.18632/oncotarget.7926)
Supplement: Supplementary file 12 [file oncotarget-07-22271-s012.docx]

**Supplementary Table 3 Meta-analyses based on sorted ethnic subgroups using allelic contrasts for negative SNVs (as of August 1, 2015 )**

| Gene | SNVs | Ancestry | OR (95% c.i.),  *P*-value ^b^ | Q-value ^c^ | Heterogeneity ^d^ *P*-value | Cases versus controls (Number of independent samples) |
| --- | --- | --- | --- | --- | --- | --- |
| *MGMT ^a^* | rs12917 | Asian | 1.911 (1.182–3.090)  *P* = 0.008 | 0.000 | 1.000 | 161 vs. 246 (1) |
|  |  | Caucasian | 0.951 (0.809–1.119)  *P* = 0.548 | 0.000 | 1.000 | 1250 vs. 1237(1) |
|  |  | African | 1.211 (0.701–2.093)  *P* = 0.493 | 0.000 | 1.000 | 147 vs. 81 (1) |
| *XRCC1^a^* | rs25487 | Asian | 1.226 (1.106–1.358)  *P* = 0.000 | 4.995 | 0.544 | 1615 vs. 1815 (7) |
|  |  | Caucasian | 1.058 (0.898–1.246)  *P* = 0.503 | 8.116 | 0.087 | 2124 vs. 1865 (5) |
|  |  | African | 0.885 (0.619–1.266)  *P* = 0.505 | 0.358 | 0.550 | 267 vs. 197 (2) |
|  |  | Mixed | 0.926 (0.798–1.437)  *P* = 0.311 | 1.140 | 0.566 | 904 vs.858 (3) |
| *OGG1^a^* | rs1052133 | Asian | 1.249 (1.077–1.449)  *P* = 0.003 | 2.141 | 0.544 | 753 vs. 840 (4) |
|  |  | Caucasian | 1.397 (0.702–2.780)  *P* = 0.342 | 20.848 | 0.000 | 498 vs. 556 (3) |
|  |  | African | 1.027 (0.759–1.390)  *P* = 0.864 | 0.000 | 1.000 | 194 vs. 646 (1) |
|  |  | Mixed | 0.875 (0.781–0.981)  *P* = 0.021 | 1.218 | 0.544 | 1626 vs.1766 (3) |
| *XRCC1* | rs1799782 | Asian | 1.139 (0.903–1.436)  *P* = 0.271 | 10.509 | 0.033 | 753 vs. 840 (4) |
|  |  | Caucasian | 0.889 (0.722–1.094)  *P* = 0.267 | 0.009 | 0.925 | 1698 vs. 1373 (2) |
|  |  | African | 0.637 (0.296–1.374)  *P* = 0.251 | 0.000 | 1.000 | 146 vs. 83 (1) |
|  |  | Mixed | 0.653 (0.304–1.403)  *P* = 0.275 | 0.000 | 1.000 | 76 vs.182 (1) |
| *ERCC2^a^* | rs1799793 | Asian | 1.539 (1.241–1.907)  *P* = 0.000 | 0.103 | 0.749 | 294 vs. 679 (2) |
|  |  | Caucasian | 0.977 (0.842–1.133)  *P* = 0.755 | 3.386 | 0.184 | 1927 vs. 1790 (3) |
|  |  | African | 1.295 (0.979–1.712)  *P* = 0.070 | 0.701 | 0.704 | 399 vs. 756 (3) |
| *XPC* | rs2228001 | Asian | 1.103 (0.949–1.282)  *P* = 0.201 | 5.877 | 0.118 | 791 vs. 874 (4) |
|  |  | Caucasian | 1.034 (0.924–1.158)  *P* = 0.559 | 0.000 | 1.000 | 1257 vs. 1251 (1) |
|  |  | African | 0.909 (0.606–1.362)  *P* = 0.644 | 0.000 | 1.000 | 147 vs. 83 (1) |
| *MGMT* | rs2308321 | Asian | 2.182 (0.687–6.936)  *P* = 0.186 | 0.000 | 1.000 | 161 vs. 248 (1) |
|  |  | Caucasian | 1.044 (0.903–1.207)  *P* = 0.562 | 0.523 | 0.469 | 1519 vs. 2681 (2) |
|  |  | African | 0.810 (0.302–2.170)  *P* = 0.674 | 0.000 | 1.000 | 140 vs. 80 (1) |
| *XRCC1* | rs25489 | Asian | 1.065 (0.932–1.218)  *P* = 0.354 | 1.180 | 0.758 | 1146 vs. 1257 (4) |
|  |  | Caucasian | 1.172 (0.902–1.522)  *P* = 0.235 | 0.000 | 1.000 | 1244 vs. 1253 (1) |
|  |  | African | 0.722 (0.264–1.977)  *P* = 0.527 | 0.000 | 1.000 | 146 vs. 83 (1) |
|  |  | Mixed | 1.354 (0.610–3.005)  *P* = 0.457 | 0.000 | 1.000 | 76 vs.182 (1) |
| *KLK3* | rs266882 | Asian | 0.786 (0.341–1.812)  *P* = 0.572 | 8.036 | 0.005 | 422 vs. 350 (2) |
|  |  | Caucasian | 1.149 (0.882–1.497)  *P* = 0.302 | 19.084 | 0.001 | 1421 vs. 1409 (5) |
|  |  | African | 0.997 (0.842–1.180)  *P* = 0.971 | 0.000 | 1.000 | 557 vs. 523 (1) |
|  |  | Mixed | 1.002 (0.834–1.203)  *P* = 0.984 | 0.000 | 1.000 | 439 vs.479 (1) |
| *SRD5A2* | rs523349 | Asian | 1.057 (0.807–1.384)  *P* = 0.687 | 14.343 | 0.006 | 767 vs. 945 (5) |
|  |  | Caucasian | 1.077 (0.985–1.177)  *P* = 0.102 | 47.363 | 0.000 | 1421 vs. 1409 (5) |
|  |  | African | 1.122 (0.957–1.317)  *P* = 0.157 | 3.014 | 0.555 | 784 vs. 961 (5) |
| *CYP17A1* | rs743572 | Asian | 0.983 (0.828–1.167)  *P* = 0.845 | 31.655 | 0.000 | 2242 vs. 2272 (10) |
|  |  | Caucasian | 0.972 (0.923–1.023)  *P* = 0.275 | 19.121 | 0.262 | 13532 vs. 13626 (17) |
|  |  | African | 1.211 (0.993–1.477)  *P* = 0.058 | 5.086 | 0.405 | 329 vs. 737 (6) |
| *ESR1* | rs2234693 | Asian | 1.096 (0.852–1.408)  *P* = 0.476 | 24.402 | 0.000 | 931 vs. 1323 (7) |
|  |  | Caucasian | 1.058 (0.966–1.158)  *P* = 0.224 | 23.546 | 0.023 | 3665 vs. 8708 (13) |
|  |  | African | 1.240 (0.935–1.644)  *P* = 0.135 | 0.224 | 0.636 | 129 vs. 422 (2) |
| *CYP3A4* | rs2740574 | African | 1.180 (0.937–1.486)  *P* = 0.160 | 25.146 | 0.001 | 1072 vs. 1278 (9) |
|  |  | Caucasian | 1.105 (0.770–1.587)  *P* = 0.588 | 0.819 | 0.366 | 581 vs. 482 (2) |
| *IL6* | rs1800795 | Asian | 1.065 (0.780–1.453)  *P* = 0.692 | 0.000 | 1.000 | 200 vs. 200 (1) |
|  |  | Caucasian | 0.821 (0.516–1.306)  *P* = 0.405 | 22.808 | 0.000 | 1216 vs. 2683 (3) |
|  |  | African | 1.361 (0.800–2.315)  *P* = 0.256 | 0.816 | 0.366 | 120 vs. 322 (2) |
|  |  | Mixed | 1.019 (0.930–1.116)  *P* = 0.683 | 5.285 | 0.152 | 9405 vs.10030 (4) |
| *IL10* | rs1800871 | Asian | 0.919 (0.794–1.064)  *P* = 0.259 | 1.629 | 0.443 | 712 vs. 820 (3) |
|  |  | Caucasian | 1.101 (0.728–1.665)  *P* = 0.650 | 6.763 | 0.009 | 969 vs. 759 (2) |
|  |  | African | 0.899 (0.730–1.107)  *P* = 0.317 | 0.689 | 0.406 | 255 vs.754 (2) |
|  |  | Mixed | 0.901 (0.801–1.013)  *P* = 0.082 | 0.000 | 1.000 | 1246 vs.1762 (1) |
| *IL10* | rs1800872 | Asian | 0.903 (0.760–1.074)  *P* = 0.249 | 0.089 | 0.766 | 553 vs. 561 (2) |
|  |  | Caucasian | 1.056 (0.936–1.192)  *P* = 0.373 | 6.764 | 0.034 | 1537 vs. 1332 (3) |
|  |  | African | 0.946 (0.771–1.164)  *P* = 0.606 | 0.385 | 0.535 | 256 vs.779 (2) |
| *IL10* | rs1800896 | Asian | 1.027 (0.588–1.792)  *P* = 0.926 | 4.095 | 0.043 | 421 vs. 529 (2) |
|  |  | Caucasian | 0.927 (0.729–1.180)  *P* = 0.540 | 7.916 | 0.019 | 1231 vs. 999 (3) |
|  |  | African | 0.972 (0.790–1.196)  *P* = 0.786 | 0.083 | 0.773 | 258 vs.789 (2) |
|  |  | Mixed | 1.054 (0.951–1.358)  *P* = 0.467 | 0.000 | 1.000 | 1245 vs.1763 (1) |
| *MPO ^a^* | rs2333227 | Caucasian | 0.687 (0.517–0.914)  *P* = 0.010 | 1.222 | 0.269 | 269vs. 346 (2) |
|  |  | Mixed | 0.925 (0.775–1.105)  *P* = 0.389 | 0.000 | 1.000 | 493 vs.1332 (1) |
| *COMT* | rs4680 | Asian | 1.103 (0.934–1.304)  *P* = 0.248 | 4.068 | 0.131 | 698 vs. 587 (3) |
|  |  | Caucasian | 0.897 (0.796–1.010)  *P* = 0.073 | 0.249 | 0.883 | 1155 vs. 1092 (3) |
|  |  | Mixed | 0.997 (0.830–1.198)  *P* = 0.977 | 0.000 | 1.000 | 439 vs.479 (1) |
| *ADIPOQ* | rs266729 | Asian | 1.004 (0.874–1.152)  *P* = 0.959 | 0.000 | 1.000 | 917 vs. 1036 (1) |
|  |  | Caucasian | 0.957 (0.705–1.300)  *P* = 0.780 | 10.536 | 0.005 | 1780 vs. 1938 (3) |
| *RNASEL* | rs486907 | Asian | 0.753 (0.454–1.250)  *P* = 0.273 | 0.000 | 1.000 | 101 vs. 105 (1) |
|  |  | Caucasian | 1.040 (0.935–1.157)  *P* = 0.467 | 10.484 | 0.063 | 3751 vs. 3731 (6) |
|  |  | African | 1.553 (0.829–2.908)  *P* = 0.169 | 3.677 | 0.055 | 223 vs. 441 (2) |
| *RNASEL ^a^* | rs627928 | Asian | 0.600 (0.389–0.925)  *P* = 0.021 | 0.000 | 1.000 | 101 vs. 105 (1) |
|  |  | Caucasian | 1.039 (0.963–1.122)  *P* = 0.324 | 2.149 | 0.708 | 2765 vs. 2607 (5) |
|  |  | African | 1.369 (1.077–1.741)  *P* = 0.010 | 0.924 | 0.337 | 223 vs. 442 (2) |
| *TP53* | rs1042522 | Asian | 0.701 (0.443–1.111)  *P* = 0.130 | 40.070 | 0.000 | 761 vs. 853 (5) |
|  |  | Caucasian | 0.957 (0.689–1.331)  *P* = 0.795 | 12.319 | 0.015 | 513 vs. 690 (5) |
| *CASC8 ^a^* | rs1447295 | African | 0.867 (0.718–1.047)  *P* = 0.138 | 0.320 | 0.571 | 443 vs. 527 (2) |
|  |  | Asian | 1.426 (1.221–1.667)  *P* = 0.000 | 5.692 | 0.223 | 1640 vs. 1432 (5) |
|  |  | Caucasian | 1.130 (0.787–1.622)  *P* = 0.509 | 76.231 | 0.000 | 4859 vs. 3663 (7) |
| *NQO1* | rs1800566 | Asian | 1.377 (0.952–1.991)  *P* = 0.089 | 2.214 | 0.037 | 251 vs. 890 (2) |
|  |  | Caucasian | 1.042 (0.806–1.347)  *P* = 0.752 | 4.166 | 0.244 | 466 vs. 904 (4) |
| *MTHFR* | rs1801131 | Asian | 1.016 (0.814–1.269)  *P* = 0.886 | 1.325 | 0.250 | 435 vs. 656 (2) |
|  |  | Caucasian | 0.948 (0.720–1.247)  *P* = 0.700 | 1.368 | 0.242 | 278 vs. 458 (2) |
| *MTHFR ^a^* | rs1801133 | Asian | 0.684 (0.565–0.828)  *P* = 0.000 | 0.513 | 0.474 | 435 vs. 656 (2) |
|  |  | Caucasian | 0.972 (0.545–1.267)  *P* = 0.833 | 9.826 | 0.020 | 3050 vs. 2036 (4) |
| *MTR* | rs1805087 | Asian | 1.078 (0.666–1.747)  *P* = 0.759 | 0.000 | 1.000 | 217 vs. 220 (1) |
|  |  | Caucasian | 1.051 (0.816–1.352)  *P* = 0.702 | 3.246 | 0.072 | 474 vs. 395 (2) |
| *MDM2 ^a^* | rs2279744 | Asian | 0.906 (0.597–1.374)  *P* = 0.641 | 4.959 | 0.026 | 401 vs. 492 (2) |
|  |  | Caucasian | 0.766 (0.612–0.959)  *P* = 0.020 | 0.011 | 0.918 | 331 vs. 344 (2) |
| *CYP1A1* | rs4646903 | African | 0.889 (0.514–1.540)  *P* = 0.675 | 0.000 | 1.000 | 138 vs.138 (1) |
|  |  | Asian | 1.124 (0.971–1.300)  *P* = 0.118 | 1.412 | 0.842 | 748 vs. 891 (5) |
|  |  | Caucasian | 0.820 (0.369–1.822)  *P* = 0.626 | 11.315 | 0.001 | 1491 vs. 1416 (2) |
|  |  | Mixed | 1.185 (0.558–2.518)  *P* = 0.658 | 5.489 | 0.019 | 227 vs. 230 (2) |
| *CASC8 ^a^* | rs6983267 | African | 0.856 (0.594–1.232)  *P* = 0.402 | 0.013 | 0.910 | 432 vs. 515 (2) |
|  |  | Asian | 1.148 (1.006–1.311)  *P* = 0.041 | 6.242 | 0.182 | 1640 vs. 1428 (5) |
|  |  | Caucasian | 0.878 (0.728–1.059)  *P* = 0.175 | 22.495 | 0.000 | 3765 vs. 2669 (5) |
| *CDH1 ^a^* | rs16260 | African | 0.424 (0.221–0.814)  *P* = 0.010 | 0.000 | 1.000 | 49 vs.117 (1) |
|  |  | Asian | 1.278 (0.915–1.785)  *P* = 0.150 | 0.000 | 1.000 | 219 vs. 219 (1) |
|  |  | Caucasian | 1.122 (0.849–1.483)  *P* = 0.417 | 9.334 | 0.009 | 1683 vs. 1300 (3) |
|  |  | Mixed | 1.242 (0.961–1.605)  *P* = 0.098 | 0.000 | 1.000 | 427 vs. 337 (1) |
| *PTGS2 ^a^* | rs20417 | African | 0.929 (0.674–1.279)  *P* = 0.650 | 0.002 | 0.964 | 359 vs.359 (2) |
|  |  | Asian | 0.542 (0.326–0.903)  *P* = 0.019 | 0.000 | 1.000 | 218 vs. 436 (1) |
|  |  | Caucasian | 1.080 (0.851–1.372)  *P* = 0.526 | 5.896 | 0.117 | 2048 vs. 3660 (4) |
|  |  | Mixed | 1.015 (0.958–1.076)  *P* = 0.607 | 0.000 | 1.000 | 7975 vs. 8566 (1) |
| *XRCC3* | rs861539 | Asian | 0.764 (0.555–1.053)  *P* = 0.100 | 0.000 | 1.000 | 224 vs. 192 (1) |
|  |  | Caucasian | 0.931 (0.680–1.274)  *P* = 0.654 | 0.170 | 0.680 | 275 vs. 379 (2) |
| *THADA ^a^* | rs1465618 | Asian | 0.843 (0.755–0.942)  *P* = 0.003 | 0.979 | 0.322 | 1393 vs. 1802 (2) |
|  |  | Caucasian | 1.124 (1.071–1.179)  *P* = 0.000 | 0.000 | 1.000 | 9758 vs. 9627 (1) |
| *NOS3* | rs1799983 | African | 0.760 (0.457–1.265)  *P* = 0.291 | 0.000 | 1.000 | 97 vs.373 (1) |
|  |  | Caucasian | 1.022 (0.923–1.131)  *P* = 0.680 | 1.845 | 0.870 | 1695 vs. 2038 (6) |
| *PTGS2 ^a^* | rs2745557 | African | 0.528 (0.294–0.946)  *P* = 0.032 | 0.000 | 1.000 | 89 vs.89 (1) |
|  |  | Asian | 0.899 (0.642–1.259)  *P* = 0.534 | 0.000 | 1.000 | 218 vs. 436 (1) |
|  |  | Caucasian | 0.929 (0.676–1.278)  *P* = 0.652 | 4.451 | 0.035 | 1772 vs. 1182 (2) |
|  |  | Mixed | 0.973 (0.918–1.032)  *P* = 0.361 | 0.000 | 1.000 | 7941 vs. 8527 (1) |
| *JAZF1 ^a^* | rs10486567 | African | 0.855 (0.787–0.929)  *P* = 0.000 | 0.394 | 0.530 | 3127 vs.2620 (2) |
|  |  | Asian | 1.237 (0.976–1.567)  *P* = 0.078 | 1.577 | 0.209 | 1387 vs. 1678 (2) |
|  |  | Caucasian | 0.847 (0.808–0.889)  *P* = 0.000 | 0.000 | 1.000 | 10286 vs. 9175 (1) |
| *C2orf43 ^a^* | rs13385191 | Asian | 0.965 (0.764–1.218)  *P* = 0.764 | 3.933 | 0.047 | 3289 vs. 5702 (2) |
|  |  | Caucasian | 1.072 (1.019–1.128)  *P* = 0.007 | 0.000 | 1.000 | 7808 vs. 8017 (1) |
| *PTGS2* | rs5275 | Asian | 1.297 (0.988–1.702)  *P* = 0.062 | 0.000 | 1.000 | 195 vs. 250 (1) |
|  |  | Caucasian | 1.005 (0.967–1.044)  *P* = 0.810 | 1.985 | 0.371 | 1520 vs. 11740 (3) |
|  |  | Mixed | 1.047 (0.874–1.253)  *P* = 0.621 | 0.000 | 1.000 | 505 vs. 506 (1) |

Note: a, the SNVs which identified as negative variants in initial meta-analyses combining all ethnical groups showed positive significance in one or two subgroups. b, the summary OR and 95% c.i. values. c, Q statistic across crude ORs was calculated across all included study. d, *P* > 0.1 is usually considered as an evidence for no between-study heterogeneity; *P* < 0.1 as an evidence for significant between-study heterogeneity. 'Q statistic=0 and *P=*1' indicated that a SNV was only investigated once in one ethnic group.
