# Supplementary material for: Systematic meta-analyses of gene-specific genetic association studies in prostate cancer
Source: Oncotarget. 2016 Mar 5;7(16):22271–84. doi: 10.18632/oncotarget.7926 (PMC5008361; doi:10.18632/oncotarget.7926)
Supplement: Supplementary file 13 [file oncotarget-07-22271-s013.docx]

**Supplementary Table 4** **heterogeneity correction analyses.** Summary ORs and 95% c.i. were calculated using random effects with allelic contrasts. A *P-*value of <0.1 in Q-statistic was considered as presence of significant between-study heterogeneity. Name followed by (a) or (b) or (c) represented the same author performed different studies. SNV, single nucleotide variant.

^a^ For these variants, heterogeneity cannot be corrected until 2 studies left.

The following studies were iteratively excluded to reach homogeneity in all ethnicities:

| *IGFBP3* | rs2854744 | Schildkraut (a)  Schildkraut (b)  Park  Safarinejad | 2005  2005  2010  2011 |
| --- | --- | --- | --- |
| *ESR1* | rs9340799 | Suzuki  Sissung  Hernandez (a)  Beuten(c)  Szendroi  Modugno  Gupta  Fukatsu  Hernandez(c)  Safarinejad  Beuten(b)  Jurecekova | 2003  2011  2006  2009  2011  2001  2010  2004  2006  2012  2009  2013 |
| *SOD2* | rs4880 | Ergen | 2007 |
| *CAT* | rs1001179 | Tefik | 2013 |
| *CYP1B1* | rs1056836 | Tang  Sobti  Chang  Tanaka  Fukatsu  Cicek  Berndt | 2000  2006  2003  2002  2004  2005  2007 |
| *VDR* | rs1544410 | Holt(b)  Oakley-Girvan(a)  Ingles  Bai  Habuchi  ONEN | 2009  2004  1998  2009  2000  2008 |
| *HNF1B* | rs4430796 | Chan  Rojas  Zhang  Zhou  Chang  Zheng  Liu(a)  Liu(b) | 2013  2014  2012  2011  2011  2010  2011  2011 |
| *RFX6* | rs339331 ^a^ | Wang(a)  Wang(b) | 2011  2013 |
| *KLK3* | rs2735839 ^a^ | Hu  Zhang  Hooker  FitzGerald  Eeles(c)  Liu  Eeles(a) | 2014  2012  2010  2009  2008  2011  2008 |

| Gene | SNV | Model | OR (95% c.i.)  *P*-value | *Q*-value | Heterogeneity  *P*-value | Cases versus controls (Number of independent samples) |
| --- | --- | --- | --- | --- | --- | --- |
| *KLK3* | rs2735839 | A vs. G, all ethnicities | 0.795 (0.694–0.911)  *P* = 0.001 | 71.124 | 0.000 | 17964 vs. 19099 (9) |
|  |  | A vs. G,  heterogeneity corrected | 0.846 (0.803–0.890)  *P* = 0.000 | 0.008 | 0.928 | 11822 vs. 12470 (2) |
| *IGFBP3* | rs2854744 | C vs. A, all ethnicities | 1.169 (1.047–1.304)  *P* = 0.005 | 13.721 | 0.089 | 2788 vs. 3020 (9) |
|  |  | C vs. A,  heterogeneity corrected | 1.100 (1.008–1.200)  *P* = 0.032 | 4.923 | 0.295 | 2295 vs. 2367 (5) |
| *ESR1* | rs9340799 | G vs. A , all ethnicities | 1.151 (1.028–1.288)  *P* = 0.014 | 39.394 | 0.001 | 3666 vs. 5066 (16) |
|  |  | G vs. A,  heterogeneity corrected | 0.926 (0.848–1.011)  *P* = 0.085 | 1.673 | 0.643 | 1958 vs. 2399 (4) |
| *SOD2* | rs4880 | C vs. T, all ethnicities | 1.121 (1.024–1.227)  *P* = 0.013 | 27.234 | 0.018 | 4210 vs. 6907 (15) |
|  |  | C vs. T,  heterogeneity corrected | 1.073 (1.014–1.134)  *P* = 0.014 | 19.737 | 0.102 | 4160 vs. 6857 (14) |
| *CAT* | rs1001179 | T vs. C, all ethnicities | 1.211 (1.045–1.404)  *P* = 0.011 | 10.505 | 0.033 | 3867 vs.28224 (5) |
|  |  | T vs. C,  heterogeneity corrected | 1.164 (1.083–1.250)  *P* = 0.000 | 1.994 | 0.574 | 3712 vs. 28029 (4) |
| *CYP1B1* | rs1056836 | G vs. C, all ethnicities | 1.129 (1.004–1.270)  *P* = 0.042 | 36.379 | 0.000 | 5999 vs. 5438 (11) |
|  |  | G vs. C,  heterogeneity corrected | 1.128 (1.058–1.202)  *P* = 0.000 | 3.553 | 0.314 | 4361 vs. 3561 (4) |
| *VDR* | rs1544410 | A vs. G, all ethnicities | 0.896 (0.823–0.975)  *P* = 0.011 | 41.674 | 0.001 | 7270 vs. 8009 (18) |
|  |  | A vs. G,  heterogeneity corrected | 0.952 (0.905–1.000)  *P* = 0.051 | 12.577 | 0.322 | 6418 vs. 7233 (12) |
| *RFX6* | rs339331 | T vs. C, all ethnicities | 0.883 (0.852–0.916)  *P* = 0.000 | 10.733 | 0.013 | 12638 vs. 15897 (4) |
|  |  | T vs. C,  heterogeneity corrected | 0.890 (0.815–0.973)  *P* = 0.010 | 4.608 | 0.032 | 10825 vs. 13445 (2) |
| *HNF1B* | rs4430796 | G vs. A, all ethnicities | 0.859 (0.793–0.930)  *P* = 0.000 | 55.853 | 0.000 | 26822 vs. 57569 (11) |
|  |  | G vs. A,  heterogeneity corrected | 0.816 (0.794–0.838)  *P* = 0.000 | 1.542 | 0.463 | 21044 vs. 52246 (3) |
